# Encapsulation of an Electron in a Diborencine Macrocycle: Synthesis, Structure, and Reactivity

**DOI:** 10.1021/jacs.5c12718

**Published:** 2025-09-22

**Authors:** Yuhao Wu, Yi Pan, Jiachen Yao, Gan Xu, Kai-Chung Lau, Zhenpin Lu

**Affiliations:** Department of Chemistry, State Key Laboratory of Marine Pollution, 53025 City University of Hong Kong, Kowloon Tong, Hong Kong SAR, P. R. China

## Abstract

Encapsulation of a single electron within the internal cavity of a host system poses significant challenges, as the electron tends to delocalize over the surface. In this study, we successfully trap a single electron through a B–B one-electron σ-bond in a diborencine macrocycle (compound **4**). The structure of this one-electron σ-bond has been characterized using X-ray single-crystal analysis and EPR studies, indicating that this bond exhibits considerable *s*-character, with the boron atoms adopting *sp* ^3^ hybridization, as supported by DFT computations. Additionally, compound **4** demonstrates rich reactivity: it can facilitate O_2_ cleavage, generating a B–O–B cyclic product; its reaction with PhSSPh produces a B–S–B linked ring-expansion product, while reactions with PhSeSePh and quinone yield ring-contraction products. The resulting products have been fully characterized through X-ray single-crystal analysis, NMR, and HRMS spectroscopy. Finally, DFT computational studies have been performed to elucidate the reaction mechanism of this one-electron B–B bond.

## Graphical abstract

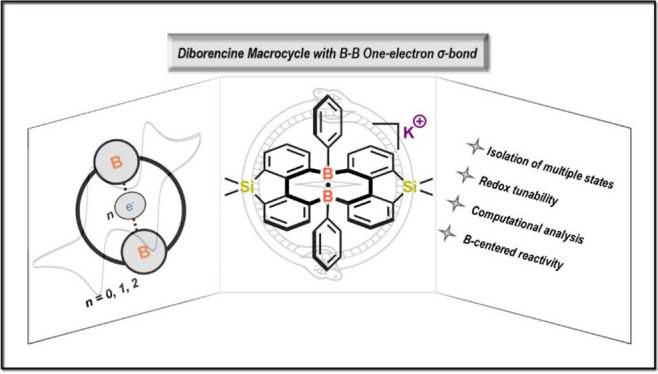

## Introduction

Macrocycles are cyclic molecules distinguished by their unique structural features and properties, making them valuable in various fields such as supramolecular chemistry, medicine, and materials science.^1–4^ One of the most intriguing properties of macrocycles is their ability to encapsulate guest molecules within their internal cavities. To date, guests of varying sizes—from single atoms (such as metal cations and anions) to larger molecules (like fullerenes)—have been successfully encapsulated by different macrocycles.^5,6^ However, the simplest and smallest guest, the electron, has yet to be precisely encapsulated in a macrocyclic system. This challenge may be attributed to the difficulty of localizing electrons internally, as they tend to delocalize on the molecular surfaces, particularly in π-conjugated systems.^7–12^

Recently, Nozaki, Okazoe, and colleagues reported the synthesis of a perfluorocubane, which exhibits an electron-accepting character within its cage, enabling the encapsulation of an electron in its internal cubic cavity (Scheme 1a).^13^ Inspired by this finding, we propose that boron-based macrocycles could impart a similar electron-accepting character to the cyclized framework, as boron atoms, with their empty *p* orbitals, are ideal candidates for spin carriers.^14^ Over the past few decades, many stable boron-centered radicals have been successfully synthesized.^15–33^ Furthermore, a single electron can be trapped through a one-electron σ-bonding,^34,35^ and diboron-based systems have been employed in the construction of B–B one-electron σ-bonds.^36–41^ For example, the Gabbaï,^42^ Wagner,^43,44^ and Légaré^45^ groups have demonstrated that diborane compounds can facilitate the formation of B–B one-electron σ-bonds (Scheme 1b). In this study, we report the synthesis of a diborencine macrocycle that successfully encapsulates an electron, forming a B–B one-electron σ-bond in compound **4** (Scheme 1c). We further explore the structural property and reactivity of compound **4.**

**Scheme 1:**
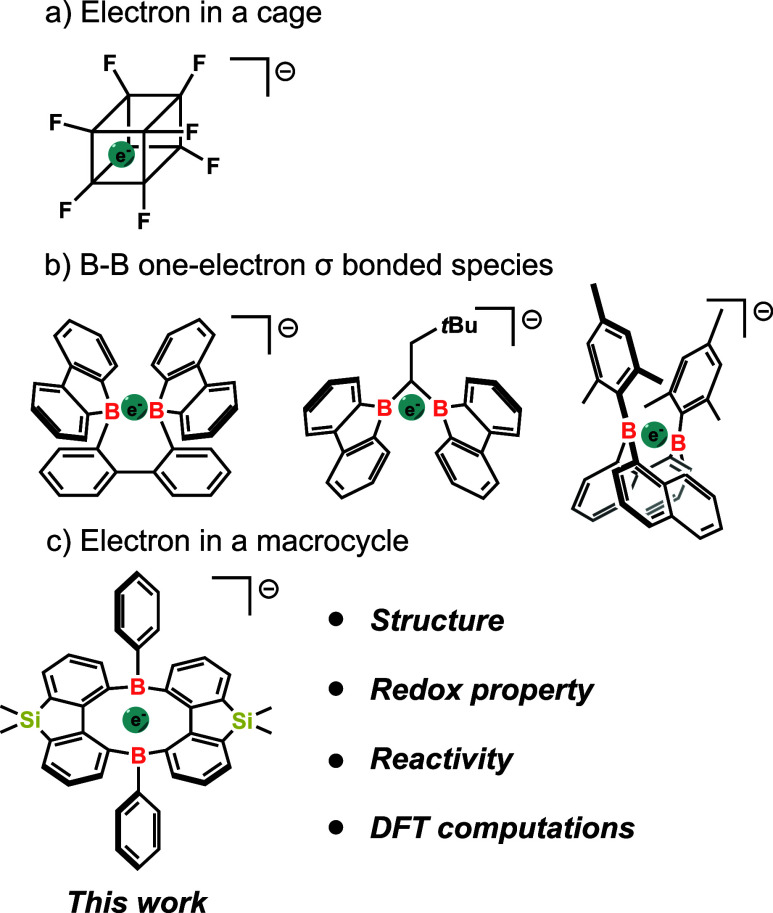
Trapping a Single Electron in Various Systems

## Results and Discussion

The key precursor, diborencine **3**, was synthesized from 1,9-dibromo-5,5-dimethyl-5H-dibenzo­[b,d]­silole (compound **1**) (Scheme 2). Compound **1** was converted to the Si, Sn-bridged diphenyl **2** through lithiation, followed by a reaction with MeSnCl_2_. Subsequently, a Sn–B exchange reaction yielded a new species, compound **3**. The ^11^B NMR spectra of **3** exhibited a signal at 69.8 ppm, indicating the formation of three-coordinated boron centers. The structure of **3** was unambiguously characterized by X-ray single-crystal analysis, revealing the formation of a ten-membered diboron macrocycle (Figure 1). Although a similar ring-expansion reaction has been reported previously,^46–50^ it is noteworthy that this method is typically used for synthesizing 9-borafluorene compounds.^51^ We inferred that the presence of ring strain in the silole structure **2** favors the formation of the ring-expansion product.

**Scheme 2:**
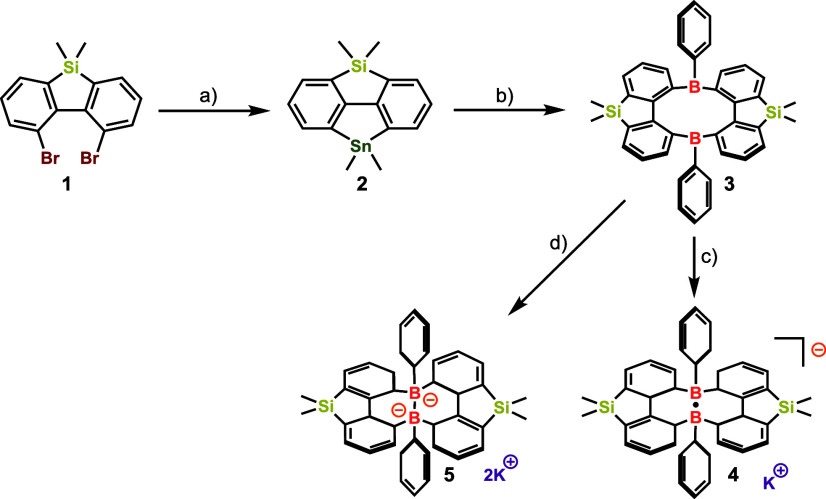
Synthesis of Compounds **4** and **5** ^a^

a a) *n*BuLi, THF, −78 °C-rt; SnMe_2_Cl_2_. b) PhBCl_2_, toluene, rt. c) 1 eq. K, THF, rt. d) 2 eq. K, THF, rt

**Figure 1:**
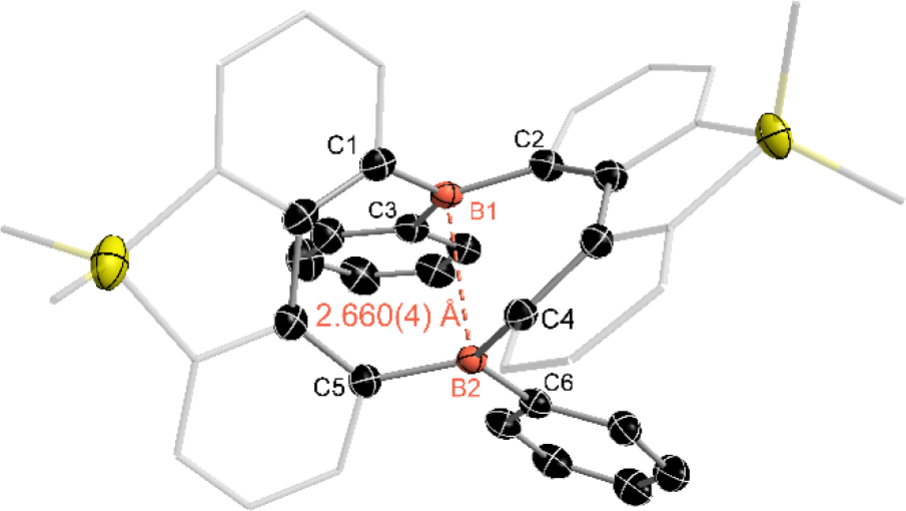
Molecular structure of compound **3**. (Thermal ellipsoids are set at the 30% probability level, and all hydrogen atoms, and solvent molecules are omitted for clarity).

The cyclic voltammogram (CV) of compound **3** reveals two reversible reduction potentials at 0.014 V vs Fc/Fc^+^ and −0.384 V vs Fc/Fc^+^ (see SI, Figure S57), indicating the formation of the stable reduction species [**3·**]^−^ and [**3**]^2^–^^ under electrochemical conditions. Encouraged by these results, a one-electron reduction of compound **3** was performed by adding 1 equiv of potassium in THF solutions. After workup, a brown solid, designated as compound **4**, was isolated as the final product. As anticipated, compound **4** is NMR silent. In contrast, the reaction of compound **3** with 2 equiv of potassium in THF produced a brown product, compound **5**. Compound **5** exhibits a signal at −8.8 ppm in the ^11^B NMR, suggesting the formation of a symmetrical structure with tetra-coordinated boron centers.

Both compounds **4** and **5** were fully characterized using X-ray single-crystal analyses (Figure 2). The B–B distance in compound **4** is 2.114(5) Å, which is significantly shorter than that of compound **3** (2.660(4) Å) but longer than the B–B distance in compound **5** (1.821(4) Å). The B–B distance in compound **4** is close to the reported values (2.265(4) and 2.166(4) Å) from the Wagner group.^43,44^ Compound **5** represents a rare example of a hexaryl-substituted diboron(6) dianion, and its B–B bond distance closely resembles that of our recently reported 9-borafluorene-based diboron(6) compound (1.890(3) Å)^52^ but is shorter than the example reported by the Légaré group (1.991(2) Å).^45^ Additionally, the sums of the C–B–C angles for B1 and B2 in compounds **3**, **4**, and **5** were compared. In compound **3**, the values are 357.62° and 358.30°. In contrast, the corresponding sums of angles in compound **4** are 346.20° and 343.86°, indicating a noticeable pyramidalization at the boron centers compared to compound **3**. Conversely, the reported B–B one-electron σ-bonded examples from the Wagner group exhibit a low degree of boron pyramidalization.^43,44^ For compound **5**, the degree of pyramidalization at the boron centers is further increased, with sums of angles measuring 333.30° and 331.23°.

**Figure 2:**
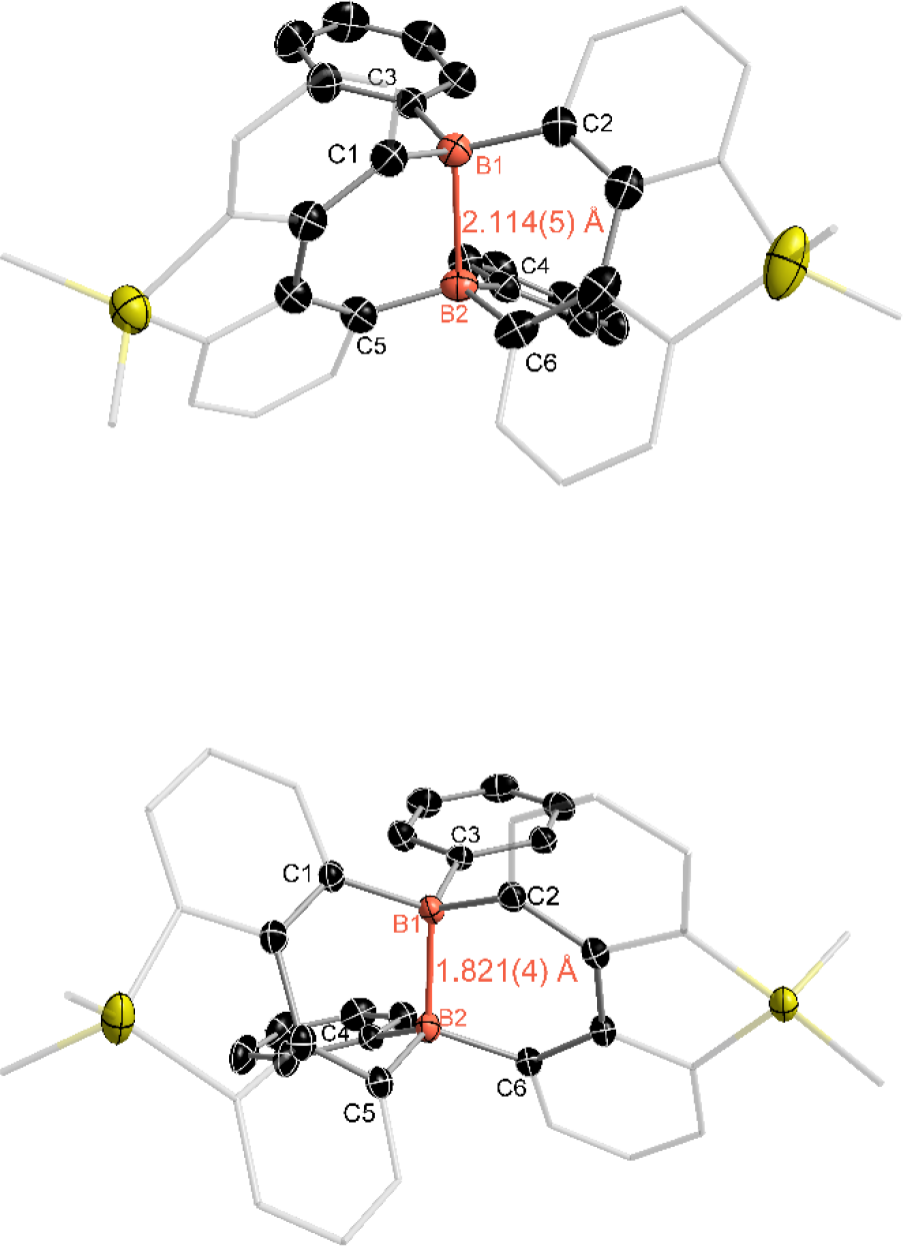
Molecular structure of compounds **4** (up) and **5** (down). (Thermal ellipsoids are set at the 30% probability level, and all hydrogen atoms, potassium cations ([K­(THF)_6_]^+^ for **4**, [K­(18-crown-6)]_2_^2+^ for **5**), and solvent molecules are omitted for clarity).

An EPR spectrum of compound **4** was recorded in THF at room temperature to characterize this radical species (Figure 3). The spectrum showed a well-resolved seven-line signal (g = 2.071), which was effectively simulated to account for the hyperfine coupling of the two boron atoms. The magnitude of the boron hyperfine coupling constant (a­(^11^B) = 24.2 G) is significantly greater than the values reported by the Wagner group (4.8 and 5.1 G),^43,44^ the Gabbaï group (5.9 G),^42^ and other related studies.^36,40^ A small value of a­(^11^B) typically indicates the preponderant participation of the boron *p*-orbitals.^42^ In contrast, a large a­(^11^B) value observed for compound **4** suggests the presence of a σ bond between borons with considerable *s* character, which aligns with the boron pyramidalization noted in the solid-state structure.

**Figure 3:**
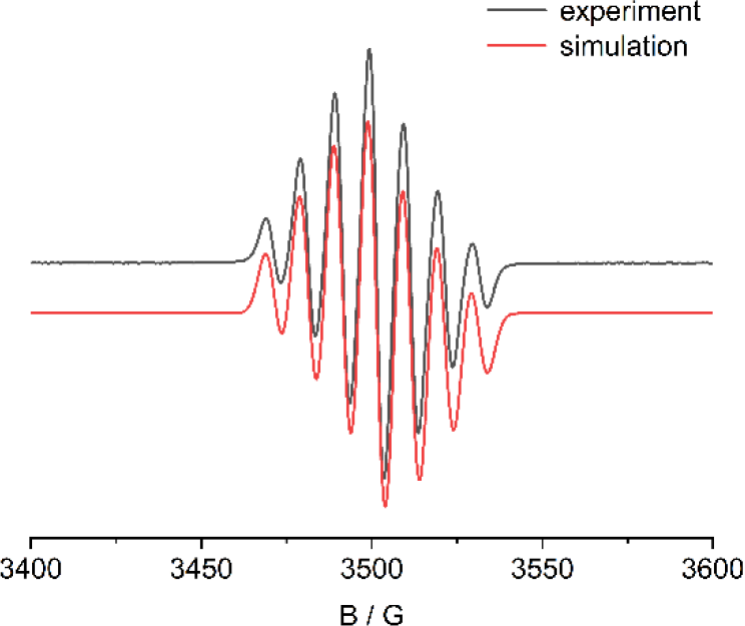
EPR spectra of compound **4.**

We conducted a detailed quantum chemical investigation using the (U)­B3LYP-D3­(BJ)/ma-TZVP//(U)­B3LYP-B3­(DJ)/ma-SVP method to better understand the B–B bond nature of compounds **4** and **5**. The optimized B–B distances and angles at the boron centers are consistent with the X-ray measured values (see SI, Table S2).

The LUMO of compound **3** (Figure 4) shows that the two B­(*p_z_*) orbitals are not aligned in a perfectly head-to-head orientation. Instead, they are slightly offset by approximately 6.5° from the B­(*p_z_*)–B­(*p_z_*) axis due to steric constraints imposed by the surrounding coordination environment of the boron atom. Upon one-electron reduction of **3**, stronger overlap between the two *p*_*z*_ orbitals occurs, forming a singly occupied σ-orbital in compound **4** (Figure 4). This is also accompanied by a decrease in the predicted B–B distance from 2.661 Å to 2.147 Å and an anticipated increase in the bond strength.

**Figure 4:**
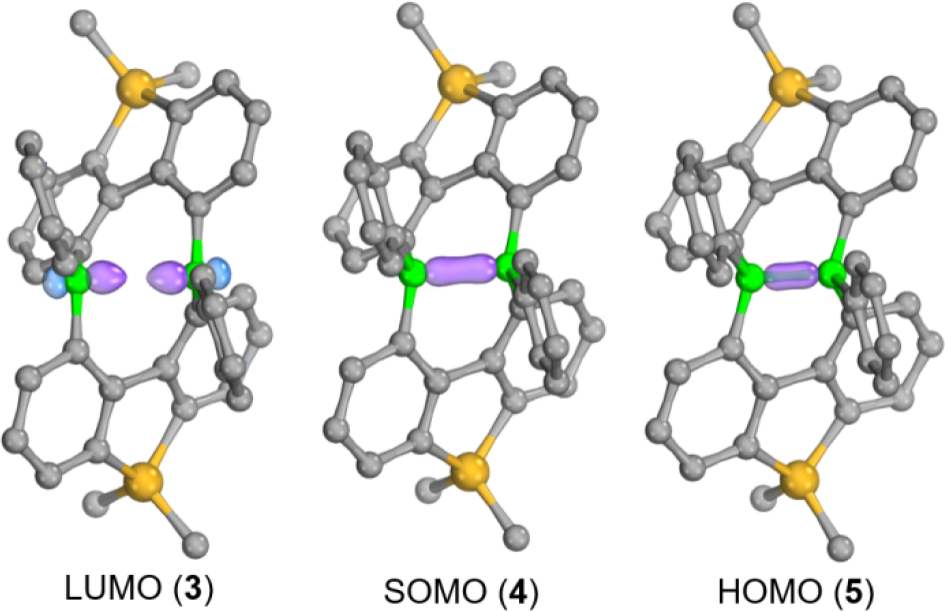
Selected molecular orbital (MO) of compound **3**, **4**, **5** at the (U)­B3LYP-D3­(BJ)/ma-TZVP//(U)­B3LYP-D3­(BJ)/ma-SVP (THF, PCM) level.

The spin density analysis at the (U)­B3LYP-D3­(BJ)/ma-SVP level indicated that *ca.* 0.37 electron is localized on each boron in the radical anion **4**. Natural bond orbital (NBO) analysis (see SI, Table S3) revealed the formation of the one-electron σ-type bond, described as σ­(B–B)=0.707­(*sp* ^5.9^)­B+0.707­(*sp* ^5.9^)­B, with significant s-character (∼14.6%). The presence of this *s*-character supports the nonplanar pyramid-like geometry around the boron centers in the compound **4.**

Likewise, the doubly occupied molecular orbital (Figure 4) of compound **5** and shorter B–B distance (1.828 Å) confirm the presence of a distinct σ bond between the two boron atoms. From the NBO analysis, the bonding hybrid at the boron is nearly pure *sp* ^3^, consistent with the observed bonding geometry.

Atoms in Molecules (AIM) analysis further supports the presence of a σ-bond in compounds **4** and **5**. No bond critical points (BCPs) were identified between the B–B interactions in compound **3**. In contrast, for compounds **4** and **5**, well-defined BCPs were observed with non-negligible electron densities (ρ­(r) = 0.053 and 0.119 au, respectively), negative energy densities (H­(r) = –0.020 and –0.070 a.u.), and negative Laplacians of electron density (∇^2^ρ­(r) = –0.036 and –0.220 a.u.). These electronic properties are consistent with covalent bonding character, albeit significantly depleted, between the boron atoms. These findings are further corroborated by noncovalent interaction (NCI) analysis, which indicates strong attractive B–B interactions in compound **4** (see SI, Figure S58).

Based on the analysis above, along with EPR spectrum information, the B–B bonding interactions in compounds **4** and **5** can be classified as *1e2c* and *2e2c* σ-bonds, respectively. The stepwise two-electron reduction of **3** occurs exclusively in the B­(*p*_*z*_) orbitals, leading to the sequential formation of the σ­(B–B) bond. Additionally, the Mayer bond order calculations qualitatively support the increasing strength of the *sp* ^3^-type σ bond upon two-electron reduction (see SI, Table S3).

To investigate the reactivity of compound **4**, its reaction with 1 atm of O_2_ was conducted in THF at room temperature (Scheme 3). After the workup, a new product was isolated, exhibiting a singlet peak at 5 ppm in the ^11^B NMR, suggesting the formation of a symmetrical structure. Crystals suitable for single-crystal X-ray analysis were obtained from an Et_2_O solution at −30 °C. The molecular structure of product **6** revealed that one oxygen atom is coordinated to two boron atoms, forming a B–O–B-linked bicyclic framework (Figure 5). Additionally, the oxygen center is three-coordinate, bonded to two boron atoms and one phenyl group, which may originate from another molecule of compound **4**. This explains why compound **6** was isolated in a low yield of 21%. Unfortunately, efforts to isolate other side products from this reaction were unsuccessful. In the literature, additional boron-based systems have also been developed for the O_2_ cleavage reaction.^53–56^

**Scheme 3:**
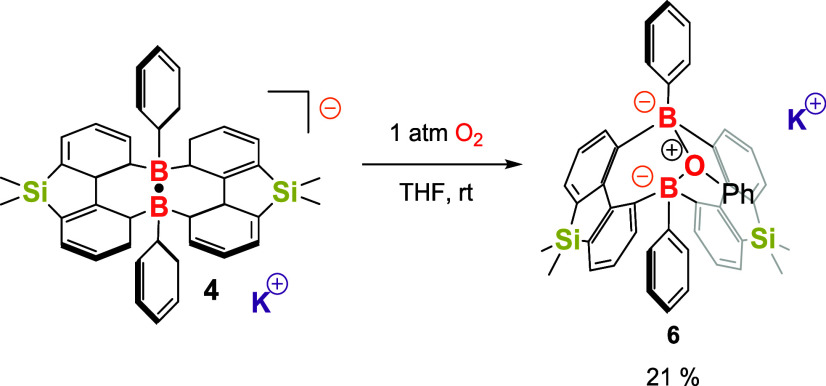
Reaction of Compound **4** with O_2_

**Figure 5:**
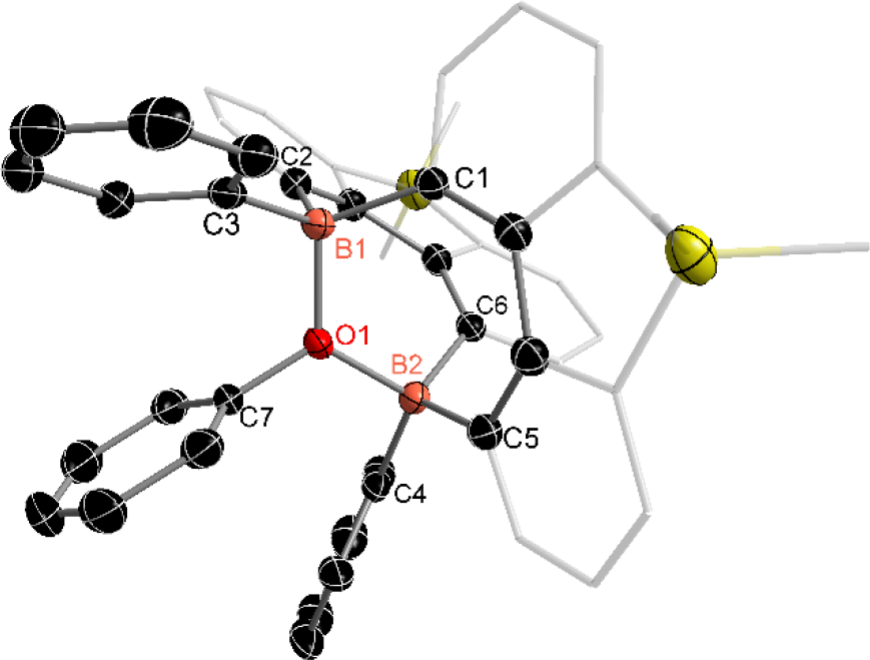
Molecular structure of compound **6**. (Thermal ellipsoids are set at the 30% probability level, and all hydrogen atoms, potassium cation ([K­(Et_2_O)_2_]^+^), and solvent molecules are omitted for clarity).

The reaction of compound **4** with PhSSPh was also conducted in THF at room temperature (Scheme 4). The final product, **7**, was isolated as a colorless solid with a 78% yield. The ^11^B NMR of **7** shows a broad signal at −3.1 ppm. In the ^29^Si NMR, two signals at 6.5 and −2.3 ppm were observed, indicating the formation of an asymmetric structure. Single-crystal X-ray analysis revealed that one PhS unit is bonded to both boron atoms, forming a B–S–B-linked seven-membered ring (Figure 6). Additionally, one phenyl group from a boron atom migrated to another boron atom, resulting in a structural rearrangement. One boron atom is incorporated into a five-membered ring system, generating a B, Si-doped fluorene. Due to the presence of two five-membered rings, the B, Si-doped fluorene framework is slightly twisted and not planar. In comparison, compound **7** can also be obtained from the reaction of compound **5** and PhSSPh (See SI, Figure S47).

**Scheme 4:**
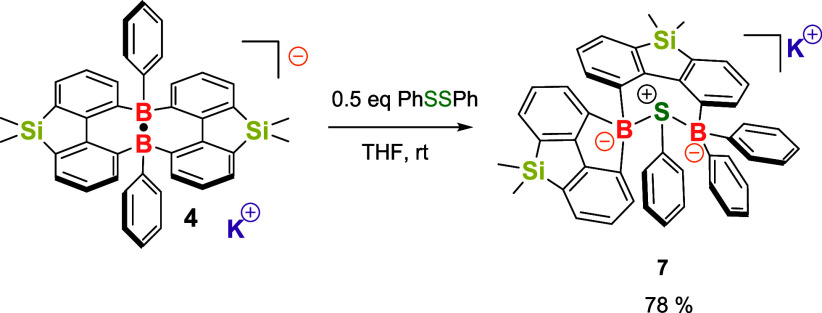
Reaction of Compound **4** and PhSSPh

**Figure 6:**
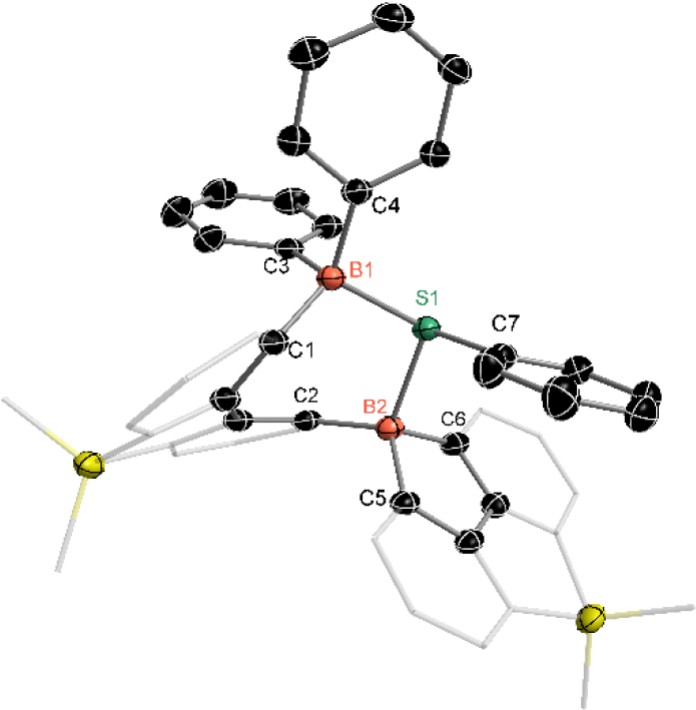
Molecular structure of compound **7**. (Thermal ellipsoids are set at the 30% probability level, and all hydrogen atoms, potassium cation, and solvent molecules are omitted for clarity).

Interestingly, the reaction of compound **4** with PhSeSePh yielded a different result compared to that of PhSSPh (Scheme 5). The final product, compound **8**, was isolated with a 38% yield and showed a signal at −2.1 ppm in the ^11^B NMR spectrum. The ^29^Si NMR spectrum of compound **8** displayed a single peak at 7.6 ppm. The molecular structure of compound **8** was confirmed through single-crystal X-ray analysis, revealing the formation of a boron- and silicon-functionalized fluorene species, featuring a tetra-coordinated boron center with a B–Se bond (Figure 7). Recently, Gilliard and colleagues reported that borafluorene radical species can react with PhSeSePh to produce boryl chalcogenides,^57^ similar to compound **8**. Additionally, the reaction of compound **5** with PhSeSePh at 55 °C in THF also yielded compound **8** as the final product (see SI, Figure S48). Similarly, the reaction of **4** with quinone produced a phenyl boryl ether with a 41% yield, compound **9**, which has been fully characterized through single-crystal X-ray analysis, NMR, and HRMS spectra. In the formation of compounds **8** and **9**, the reactivity of compound **4** can be viewed as a borafluorene-centered radical species **10** for further transformations. Additionally, other substrates, including alkenes, alkynes, nitriles, aldehydes, TEMPO radicals, and various other species, have been examined for their reactions with compound **4**. However, these reactions either resulted in undetermined products or did not yield any new products (see SI, Table S1).

**Scheme 5:**
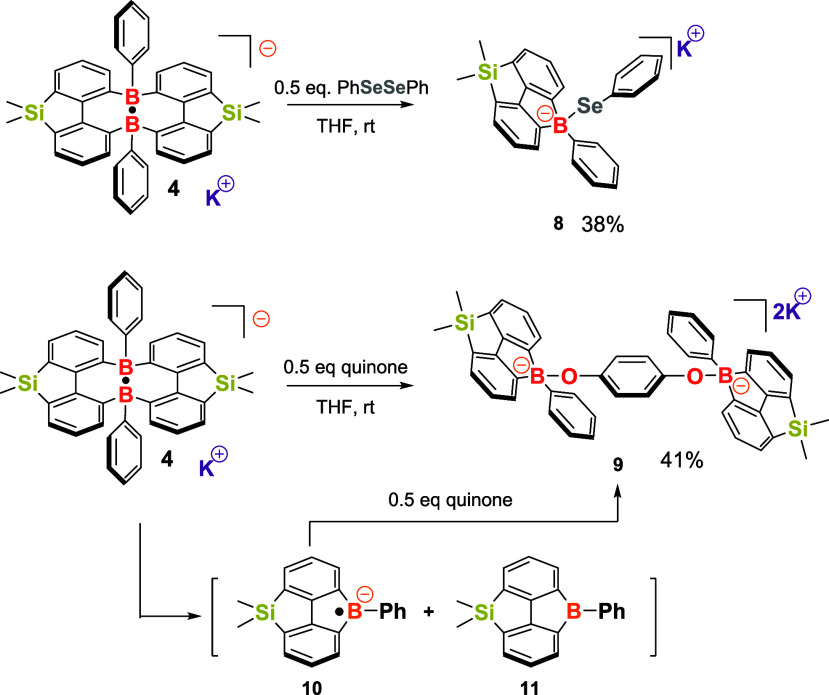
Reaction of Compound **4** with PhSeSePh/Quinone

**Figure 7:**
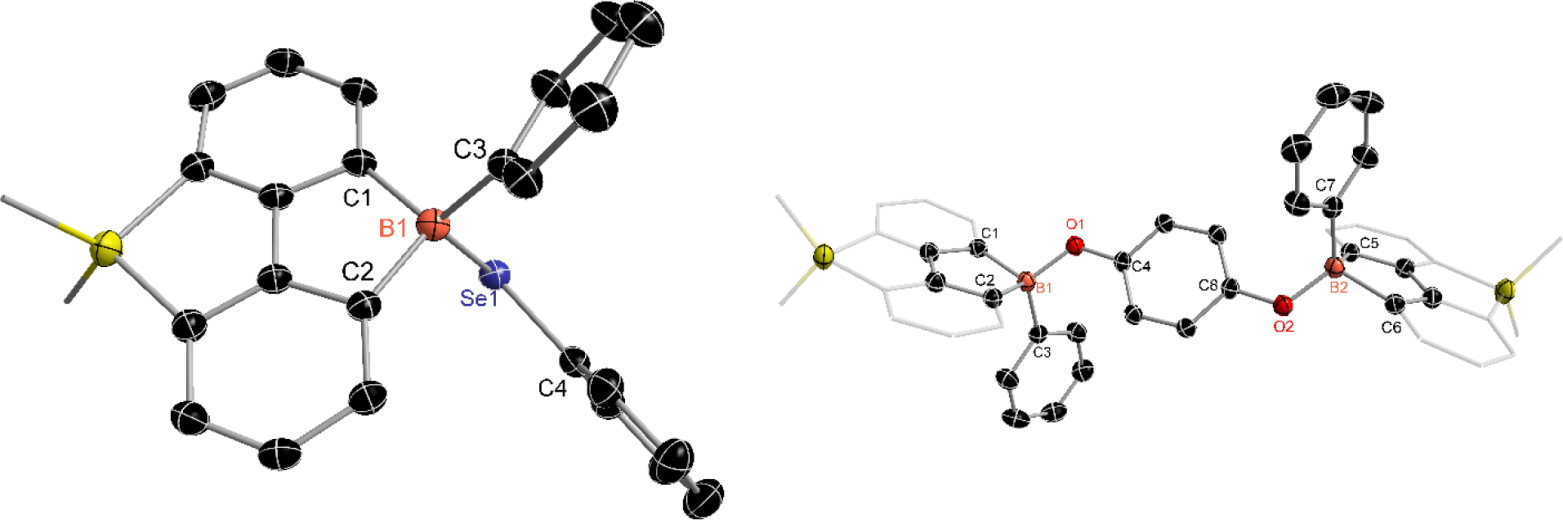
Molecular structures of compounds **8** (Left) and **9** (Right). (Thermal ellipsoids are set at the 30% probability level, and all hydrogen atoms, potassium cations­([K­(THF)_3_]^+^ for **8**, [K­(2,2,2-cryptand)]_2_^2+^ for **9**), and solvent molecules are omitted for clarity).

The potential energy surface (PES, Scheme 6) for the reaction between compound **4** and PhSSPh was studied at the (U)­B3LYP-D3­(BJ)/ma-TZVP//(U)­B3LYP-D3­(BJ)/ma-SVP (solvent is THF, PCM) level. The presence of the PhS unit in the product compound **7** suggests that the reaction involves the cleavage of the S–S bond in PhSSPh.

**Scheme 6:**
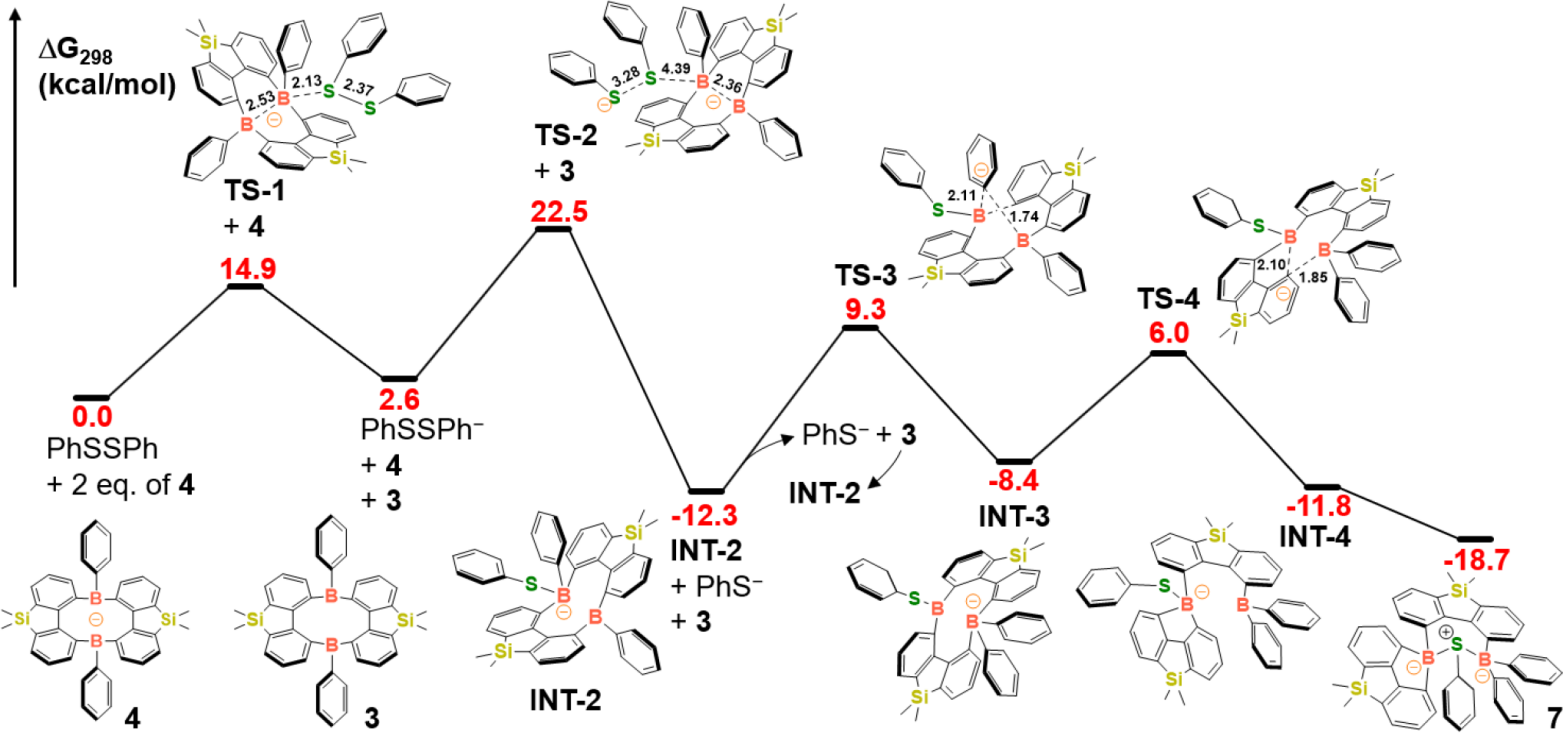
Potential Energy Surface for the Reaction between Compound **4** and PhSSPh, Calculated at the (U)­B3LYP-D3­(BJ)/ma-TZVP//(U)­B3LYP-D3­(BJ)/ma-SVP (THF, PCM) level

The mechanism initiates with a single electron transfer from one equivalent (eq.) of compound **4** to PhSSPh via transition state **TS-1** (ΔG_298_^‡^ = 14.9 kcal/mol). This step generates one eq of compound **3** and the radical anion PhSSPh^–^. Subsequently, another portion of **4** reacts with PhSSPh^–^ through an open-shell singlet transition state, **TS-2** (ΔG_298_^‡^ = 22.5 kcal/mol), yielding a key intermediate **INT-2**. The progressive elongation of the S–S bond length from 2.13 Å (in PhSSPh) to 2.37 Å (in **TS-1**) to 2.97 Å (in PhSSPh^–^) to 3.28 Å (in **TS-2**), clearly illustrates the stepwise cleavage of the S–S bond facilitated by compound **4**. This cleavage ultimately produces a thiyl radical (PhS•) and a thiophenolate anion (PhS^–^). Within the open-shell singlet configuration of **TS-2**, the lone electron (alpha spin) on the PhS• radical pairs up with the only electron (beta spin) in the σ-bond of the (second eq.) compound **4**. This spin-coupling forms the B–S bond in **INT-2**, from which the final product **7** evolves via a series of molecular rearrangements and internal rotations. Following **INT-2** formation, the phenyl group (as an anion) attached to the boron atom involved in the B–S bond migrates to another boron atom within the structure, via **TS-3**, generating intermediate **INT-3**. The diphenyl-substituted boron moiety in **INT-3** then undergoes B–C bond cleavage (via **TS-4**) and an intramolecular rearrangement, leading to **INT-4** and the final product **7.**

It is noteworthy that the PhS^–^ anion and compound **3**, generated after the S–S bond cleavage step, can also combine barrierlessly to form another eq of **INT-2**. This transformation has been further confirmed by our experimental results, which show that the reaction of PhSK with compound **3** produced the desired product **7** (Figure S47). To summarize, starting from 2 eq. of compound **4** and 1 eq. of PhSSPh, the reaction stoichiometrically affords 2 eq. of **INT-2** and thus 2 eq. of compound **7**. This suggested mechanism aligns with the experimentally observed high yield (78%) for the conversion of **4** to **7.**

## Conclusions

In summary, we report the synthesis of a diborencine macrocycle (**3**), which enables the precise encapsulation of a single electron through the formation of a B–B one-electron σ-bond. Both the one- and two-electron reduction products (**4** and **5**) have been fully characterized using X-ray single-crystal analysis. EPR and computational studies were conducted to understand the one-electron σ-bonding in compound **4**, revealing a significant presence of s-character. Furthermore, we examined the reactivity of compound **4**, which can facilitate dioxygen cleavage to form a B–O–B bonded product. Additionally, compound **4** can react with PhSSPh, yielding B–S–B-linked boron cyclic compounds. In contrast, during reactions with PhSeSePh and quinone, compound **4** acts as a borafluorene radical (**10**) for further transformations. DFT computational studies provide insights into the reaction mechanism for the formation of compound **7**, highlighting the crucial role of this one-electron σ-bond in initiating a single-electron transfer process.

## Supplementary Material



## Notes

Supplementary figures, detailed experimental procedures, characterization data for all new compounds (PDF)
