# Supplementary material for: Encapsulation of an Electron in a Diborencine Macrocycle: Synthesis, Structure, and Reactivity
Source: J Am Chem Soc. 2025 Sep 22;147(39):35895–902. doi: 10.1021/jacs.5c12718 (PMC12498403; doi:10.1021/jacs.5c12718)
Supplement: Supplementary file 1 [file ja5c12718_si_001.pdf]

Supporting Information
for

Encapsulation of An Electron in A Diborencine Macrocycle: Synthesis, Structure, and Reactivity

Yuhao Wu,[1‡] Yi Pan,[1‡] Jiachen Yao,[1] Gan Xu,[1] Kai-Chung Lau*[1] and Zhenpin Lu*[1]

[1] Department of Chemistry, State Key Laboratory of Marine Pollution, City University of Hong Kong, Kowloon Tong, Hong Kong SAR, P. R. China

KEYWORDS. Boron • Reactivity • One-electron σ-bond • DFT calculations.

Table of contents

1. Experimental Section 3

1.1. General Considerations 3

1.2. Synthetic Procedures 4

2. NMR Spectra 14

3. X-Ray Crystallography Details 39

3.1. X-ray Crystallography Collection and Refinement Details 39

3.2. X-ray Data 40

4. Cyclic Voltammetry Spectrum of 3 50

5. Computational Section 51

6. Reference 78

1. Experimental Section

1.1. General Considerations

All air- and moisture-sensitive manipulations were carried out using vacuum line, Schlenk, and cannula techniques, or in a Vigor inert atmosphere (argon) glove box equipped with a -37 °C freezer. All glassware was stored in a pre-heated (115 °C) oven or flame-dried prior to use. Diethyl ether (Et_2O), tetrahydrofuran (THF), toluene, and *n*-hexane were purified by distillation over sodium and benzophenone and stored over 4 Å molecular sieves in PTFE-sealed glass vessels for use in the glovebox. Other anhydrous solvents were distilled and degassed using appropriate drying agents. [1] Benzene-d_6, THF-d_8, and $CDCl_3$ were degassed through three freeze-pump-thaw cycles before being stored in an inert atmosphere glove box. Benzene-d_6 and THF-d_8 were dried over potassium-sodium alloy before use. Unless otherwise noted, other chemicals obtained from commercial suppliers (TCI, Aladdin, Sigma Aldrich, Alfa Aesar, *etc.*) were used without further purification. 2,2',6,6'-tetrabromo-1,1'-biphenyl was prepared *via* Sandmeyer reaction[2] and Ullmann-type homocoupling[3] using 2,6-dibromoaniline as the starting material.

Unless otherwise noted, all NMR spectra were recorded at ambient temperature with the following spectrometers: Bruker 300MHz *AVANCE III HD*, 400MHz *AVANCE III*, or 600MHz *ASCEND AVANCE III HD*. Chemical shifts are referenced to residual solvent signals ($^1H/^{13}C\{^1H\}$, $CDCl_3$: 7.26/77.16 ppm, C_6D_6: 7.16/128.06 ppm, THF-d_8: 3.58/67.57 ppm) and all other NMR spectra externally to $SiMe_4$ (0.00 ppm) or external $BF_3{\cdot}Et_2O$ (^{11}B: δ = 0.00 ppm). ^1H and ^{13}C NMR data are reported as follows: chemical shift, multiplicity (s = singlet, d = doublet, t = triplet, q = quartet, m = multiplet, br = broad, ov = overlapping), assignment of resonances was supplemented by ^1H-^1H COSY and ^1H-$^{13}C\{^1H\}$ HSQC/HMBC experiments. Monitoring of air-moisture sensitive compounds was carried out using *J-Young* NMR tubes assembled in a glove box. High-resolution mass spectral data were obtained on a Sciex X500R Q-TOF mass spectrometer. MALDI-MS measurements were performed on a Bruker AutoFlex MAX in linear mode. Trans-2-[3-(4-tert Butylphenyl)-2-methyl-2-propenylidene]malononitrile (10 mg/mL) used as the matrix was mixed with the samples, and then spotted on the wells of a target plate inside a glove box. UV-Visible absorption spectra were recorded on PE Lambda 1050 Spectrophotometer. Samples were prepared inside the glovebox in 1 cm square quartz cuvettes with Teflon screw caps. EPR measurements were carried out using a Bruker EMXPlus-10/12 EPR spectrometer. The spectral simulations were performed using MATLAB R2024b and the EasySpin toolbox. [4] Electrochemical measurements were conducted on a CHI660 electrochemical workstation. A standard three-electrode cell configuration was employed using a glass carbon working electrode, a Pt wire auxiliary electrode, and a silver reference electrode. All tests were performed using $n$$Bu_4NPF_6$ (0.1 M) as the supporting electrolyte. The potentials are reported relative to the ferrocene/ferrocenium couple.

1.2. Synthetic Procedures

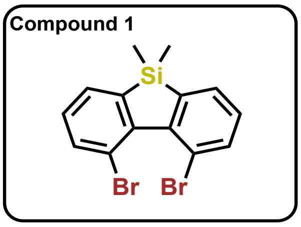

1,9-dibromo-5,5-dimethyl-5*H*-dibenzo[*b,d*]silole, compound 1. To a solution of 2,2',6,6'-tetrabromo-1,1'-biphenyl (0.82 g, 1.76 mmol) in Et_2O (40 mL), *n*-BuLi (1.6 M in hexane, 2.2 mL, 3.52 mmol) was added dropwise at -78°C over a period of 20 min, and the mixture was then stirred at this temperature for 2 h. Me_2SiCl_2 (0.24 g, 1.85 mmol) was subsequently added dropwise to the mixture at -78°C. The mixture was gradually warmed to room temperature and stirred for 8 h. After removing the volatiles under reduced pressure, the resulting solid was subjected to flash column chromatography on silica gel using *n*-hexane as an eluent (Rf =0.50) to afford 0.48 g (1.31 mmol, 74% yield) of **1** as a colorless crystalline solid.

^{1}H NMR (300 MHz, $CDCl_3$) δ 7.67 (dd, J = 7.9, 1.2 Hz, 2H), 7.57 (dd, J = 6.9, 1.1 Hz, 2H), 7.19 (dd, J = 7.9, 6.8 Hz, 2H), 0.40 (s, 6H); ^{13}C NMR (75 MHz, $CDCl_3$) δ 148.0, 144.8, 136.1, 130.8, 129.0, 120.5, -2.4; HRMS (m/z): Calc. For $[M+H]^+$ ($[C_{14}H_{13}Br_2Si]^+$): 368.9127 Found: 368.9094.

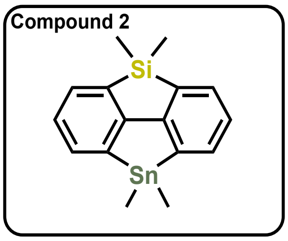

4,4,8,8-tetramethyl-4,8-dihydro-4-sila-8-stannacyclopenta[*def*]fluorene, compound 2. To a solution of **1** (0.76 g, 2.08 mmol) in THF (30 mL), *n*-BuLi (1.6 M in hexane, 2.6 mL, 4.16 mmol) was added dropwise at -78°C over 20 min, and the mixture was then stirred at this temperature for 3 h. A solution of Me_2SnCl_2 (0.46 g, 2.09 mmol) in 10 mL THF was added dropwise to the mixture at -78°C. After being stirred at -78°C for 1 h, the solution was allowed to gradually warm to room temperature and stirred for 16 h, during which time the solution became cloudy. After all volatile components were removed under vacuum, the solid was dispersed in hexane. The dispersion was filtered through a pad of Celite® under an argon atmosphere. The filtrate was concentrated under reduced pressure to yield compound **2** as a colorless solid in 90 % yield (0.67 g, 1.87 mmol) with sufficient purity for the next step. Recrystallisation in *n*-hexane at -30°C gave colorless crystals in several crops, which were also suitable for X-ray diffraction analysis.

^{1}H NMR (400 MHz, C_6D_6) δ 7.61 (dd, J = 7.1, 1.1 Hz, 2H), 7.56 (dd, J = 7.1, 1.2 Hz, 2H), 7.25 (t, J = 7.1 Hz, 2H), 0.36 (s, 6H), 0.33 (s, 6H); ^{13}C NMR (100 MHz, C_6D_6) δ 161.2, 137.6, 136.4, 135.9, 133.3, 128.2, -2.8, -8.3; HRMS (m/z): Calc. For $[M+H]^+$ ($[C_{16}H_{19}SiSn]^+$): 359.0274 Found: 359.0291.

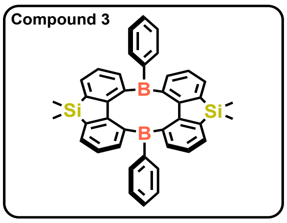

Compound 3. Inside the glovebox, a solution of compound **2** (0.66 g, 1.84 mmol) in toluene (20 mL) was added to $PhBCl_2$ (0.30 g, 1.88 mmol) in toluene (10 mL) dropwise and stirred for 18 h, during which the color of the reaction mixture changed from colorless to bright yellow. All the volatiles were removed in *vacuo,* followed by sublimation (50 °C, 1.8 × 10^{-2} mbar) to remove Me_2SnCl_2 byproduct. The crude product was triturated with hexane (2 × 4 mL). Drying the residue under high vacuum afforded **3** as a bright yellow solid (0.37 g, 0.62 mmol, 68%), and the second crop was obtained from the solution of *n*-hexane at -30 °C (0.06 g, 0.10 mmol, 11%) after several recrystallizations. Suitable single crystals for X-ray diffraction were grown by slow crystallization from toluene at -30 °C.

^{1}H NMR (600 MHz, C_6D_6) *δ* 7.64 (dd, *J* = 7.0, 1.4 Hz, 2H, ArH_1), 7.56 (dd, *J* = 7.4, 1.4 Hz, 2H, ArH_3), 7.37 (d, *J* = 7.4 Hz, 4H, PhH_4), 7.33 (dd, *J* = 6.9, 1.3 Hz, 2H, ArH_7), 7.09 (t, *J* = 7.2 Hz, 2H, ArH_2), 7.07 - 7.03 (m, 2H, PhH_6), 6.92 (t, *J* = 7.5 Hz, 4H, PhH_5), 6.74 (t, *J* = 7.2 Hz, 2H, ArH_8), 6.59 (dd, *J* = 7.5, 1.3 Hz, 2H, ArH_9), 0.47 (s, 6H, $SiCH_3$), 0.37 (s, 6H, $SiCH_3$); ^{13}C NMR (150 MHz, C_6D_6) *δ* 159.0 (ArC_{10}), 155.5 (ArC_{12}), 149.4 (ArC_{15}), 147.7 (ArC_{11}), 141.2 (ArC_{14}), 140.9 (PhC_{16}), 140.6 (PhC_4), 140.0 (ArC_3), 138.5 (ArC_{13}), 134.6 (ArC_1), 132.9 (ArC_7), 132.4 (PhC_6), 131.2 (ArC_9), 127.2 (PhC_5), 126.5 (ArC_8), 125.2 (ArC_2), -2.8 (*SiMe*), -3.0 (*SiMe*); ^{11}B NMR (193 MHz, C_6D_6) *δ* 69.8 (s, br); MALDI-TOF Mass ([$C_{40}H_{34}B_2Si_2$]): Simulated: 592.239 Found: 592.634; HRMS (m/z): Calc. For $[M+Na]^+$ ($[C_{40}H_{34}B_2NaSi_2]^+$): 615.2291 Found: 615.2224.

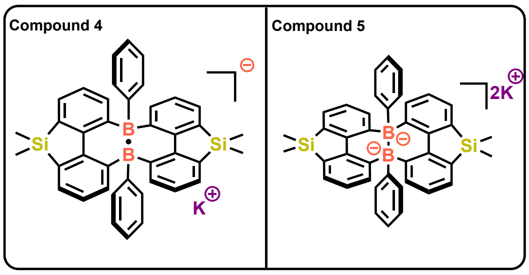

Compound 4. In a vial, recrystallized **3** (34 mg, 57 μmol, 1.01 equiv) was combined with fresh potassium (2.2 mg, 56 μmol, 1.0 equiv) in 5 mL of THF. The mixture was stirred for 10 h, during which it transitioned from yellow to a red hue. The insoluble components were separated by filtration on a glass fiber. After removing all volatiles under vacuum, the resulting foam was washed with a mixture of toluene and *n*-hexane until colorless supernatant was obtained. Drying the residue under reduced pressure yielded 26 mg (73 % yield) of **4** as a brown solid. Crystals suitable for X-ray diffraction analysis were obtained by layering hexane on a concentrated THF solution of **4** at -30 °C.
MALDI-TOF Mass ([$C_{40}H_{34}B_2KSi_2$]): Simulated: 631.203 Found: 631.574; HRMS (m/z): Calc. For [M] ($C_{40}H_{34}B_2KSi_2$): 631.2030 Found: 631.1977.

Compound 5. In a vial, fresh potassium (5.0 mg, 128 μmol, 2.0 equiv) was added to a 5 mL THF solution of **3** (38 mg, 64 μmol, 1.0 equiv). The mixture was allowed to stir at room temperature for 10 h, resulting in a change in color to a red hue. The insoluble components were removed by filtration, and the solvent was removed under reduced pressure. The pasty solid residue was recrystallized by vapor diffusion of Et_2O into a concentrated THF solution, yielding **5**·$(THF)_n$ as black crystals. Upon prolonged storage of the sample under a vacuum, exhaustively dried brown powder was collected (32 mg, 74 % yield). Single crystals suitable for X-ray diffraction analysis were obtained by slow diffusion of Et_2O into an acetonitrile solution of **5** with 2 equiv of 18-crown-6.
^{1}H NMR (600 MHz, THF-d_8) δ 7.06 (dd, J = 7.2, 1.5 Hz, 4H, ArH_1), 7.03 (dd, J = 6.9, 1.5 Hz, 4H, ArH_3), 6.69 (t, J = 7.0 Hz, 4H, ArH_2), 6.64 – 6.58 (m, 8H, PhH_4, PhH_5, ov), 6.47 – 6.44 (m, 2H, PhH_6), 0.37 (s, 6H, $SiCH_3$), 0.27 (s, 6H, $SiCH_3$); ^{13}C NMR (150 MHz, THF-d_8) δ 170.0 (PhC_{10})*, 167.1 (ArC_9)*, 157.1 (ArC_7), 138.8 (ArC_1), 134.8 (ArC_8), 134.7 (PhC_4), 126.6 (ArC_3), 126.0 (ArC_2), 125.4 (PhC_5), 118.9 (PhC_6), -1.4 (*SiMe*), -2.0 (*SiMe*); ^{29}Si NMR (119 MHz, THF-d_8) δ -2.7 (s); ^{11}B NMR (193 MHz, THF-d_8) δ -8.8 (s); HRMS (m/z): Calc. For $[M-K]^-$ ($[C_{40}H_{34}B_2KSi_2]^-$): 631.2030 Found: 631.1975.
*These signals were further confirmed through ^{1}H-^{13}C HMBC NMR spectra.

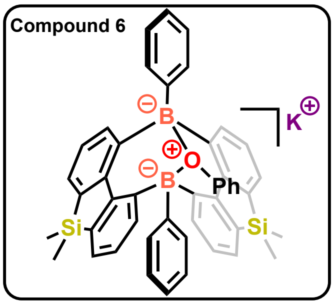

Compound 6. After three freeze-pump-thaw cycles, the THF solution of **4** (41 mg, 0.065 mmol) in a 50 mL Schlenk tube was subjected to 1 atm O_2 at room temperature while stirring. The color of the mixture changed from red to light yellow immediately. The resulting solution was allowed to stir for 6 h, and all volatiles were removed in *vacuo*. The resulting solid was redissolved in Et_2O and slowly added to an Et_2O solution of 18-crown-6. After concentration, **6** was isolated as an adduct of 18-crown-6 in Et_2O solution with several drops of *n*-hexane at -30 °C in 21% yield (10 mg). Crystalline material of **6** slowly grew from the Et_2O solution of the reaction mixture at -30 °C over two weeks.

^{1}H NMR (600 MHz, THF-d_8) δ 7.24 (s, br, 8H, $ArH_{1,2}$), 6.62 (s, br, 4H, $ArH_{1,2}$), 6.51 – 6.46 (m, 3H, $PhH_{3,3'}$), 6.44 – 6.40 (m, 4H, $BPhH_4$), 6.39 – 6.32 (m, 2H, $OPhH_5$), 6.22 – 6.18 (m, 4H, $BPhH_6$), 5.82 – 5.76 (m, 2H, $OPhH_7$), 3.44 (s, 24H, *18-crown-6*), 0.44 (s, 6H, $SiCH_3$), 0.31 (s, 6H, $SiCH_3$); ^{13}C NMR (150 MHz, THF-d_8) δ 159.1 (ArC_{12}), 157.6 ($OPhC_{10}$), 138.8 ($BPhC_2$), 137.4 (ArC_{11}), 129.5 ($OPhC_1$), 128.8 ($ArC_{8/9}$), 126.8 ($OPhC_3$), 125.0 ($BPhC_4$), 123.7 (ArC_7), 123.5 ($BPhC_5$), 122.9 ($OPhC_6$), 71.2 (*18-crown-6*), -1.1 (*SiMe*), -2.1 (*SiMe*); ^{11}B NMR (193 MHz, THF-d_8) δ 5.0 (s); MALDI-TOF Mass $[M+K]^+$ ($[C_{46}H_{39}B_2K_2OSi_2]^+$): Simulated: 763.201 Found: 762.799; HRMS (m/z): Calc. For $[M-K]^-$ ($[C_{46}H_{39}B_2OSi_2]^-$): 685.2735 Found: 685.2675.

*Due to coupling with quadrupolar nuclei, the carbon atoms adjacent to boron could not be resolved but could be observed in the HMBC spectrum.

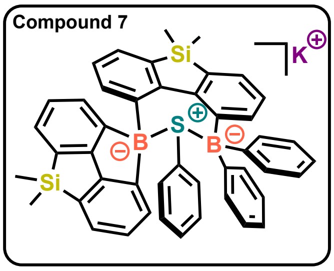

Compound 7. Recrystallized **4** (39.5mg, 0.062 mmol) was dissolved in 5 mL of THF. To this deep red solution, diphenyl disulfide (7.0 mg, 0.032 mmol) was added in one portion, and the reaction was stirred for 8 h, resulting in a gradual transition to a light-yellow solution. The THF was removed in *vacuo*, followed by two washes with 4 mL hexane. The resulting solid was recrystallized from a concentrated Et_2O solution to yield compound **7** (36 mg, 78% yield) as a colorless solid.

^{1}H NMR (600 MHz, THF-d_8) δ 7.54 (dd, J = 7.4, 1.5 Hz, 1H, ArH_1), 7.45 (dd, J = 6.8, 1.4 Hz, 1H, ArH_4), 7.42 (dd, J = 7.4, 1.5 Hz, 1H, ArH_5), 7.17 – 7.13 (m, 4H, $ArH_{13,14}$), 7.05 – 6.99 (m, 2H, ArH_2), 6.92 (d, J = 7.0 Hz, 2H, $BPhH_6$), 6.87 – 6.82 (m, 2H, $SPhH_{10}$), 6.71 – 6.64 (m, 7H, ArH_{12}, $SPhH_{11}$, $BPhH_8$), 6.60 – 6.55 (m, 4H, $BPhH_7$), 6.52 (t, J = 7.6 Hz, 2H, $SPhH_9$), 6.45 (t, J = 7.1 Hz, 1H, ArH_3), 0.29 (s, 6H, $Si(CH_3)_2$), 0.25 (s, 3H, $SiCH_3$), 0.22 (s, 3H, $SiCH_3$); ^{13}C NMR (150 MHz, THF-d_8) δ 163.1 (ArC_{18}), 163.0 ($SPhC_{20}$), 162.3 (ArC_{19}), 141.0 (ArC_1), 140.4 (ArC_{17}), 137.9 (ArC_{16}), 137.5 (ArC_{22}), 137.1 (ArC_3), 136.0 (ArC_5), 135.6 (ArC_{12}), 134.0 ($SPhC_9$), 129.7 (ArC_2), 129.5 (ArC_6), 129.2 (ArC_{21}), 128.9 ($BPhC_8$), 126.9 ($SPhC_{14}$), 126.4 (ArC_4), 126.2 ($BPhC_{10}$,$SPhC_{11}$, *ov*), 124.4 (ArC_7), 124.1 (ArC_{15}), 123.3 ($BPhC_{13}$), -1.4 (*SiMe*), -1.6 (*SiMe*), -1.8 (*SiMe*); ^{29}Si NMR (119 MHz, THF-d_8) δ 6.5, -2.3; ^{11}B NMR (193 MHz, THF-d_8) δ -0.7 (br); MALDI-TOF Mass $[M+Na+Et_2O]^+$ ($[C_{50}H_{49}B_2KNaOSSi_2]^+$): Simulated: 837.277 Found: 838.994; HRMS (m/z): Calc. For $[M-K]^-$ ($[C_{46}H_{39}B_2SSi_2]^-$): 701.2507 Found: 701.2453.

*Due to coupling with quadrupolar nuclei, the carbon atoms adjacent to boron could not be resolved but could be observed in the HMBC spectrum.

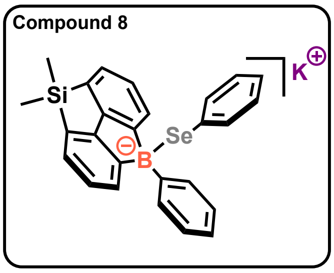

Compound 8. Inside the glovebox, **4** (39.9mg, 0.063mmol) was dissolved in THF (0.6 mL) in a J-Young NMR tube. Diphenyl diselenide (10.0mg, 0.032mmol) was added to the solution. The reaction was then heated at 55 ºC for 12 h (or stirred at room temperature for 3 d). Afterward, all volatile materials were removed under vacuum. The remaining sticky solid was washed with hexane until the wash solution became colorless. The remaining solid was dried under vacuum to afford crude **8** as a light-yellow powder. Recrystallization from the mixed solution of THF and *n*-hexane at -30℃ gave the pure product **8** as colorless crystals (12 mg, 38%), which is also suitable for X-ray diffraction.

^{1}H NMR (600 MHz, THF-d_8) δ 7.84 – 7.80 (m, 2H, *SePhH$_1$*), 7.52 (d, J = 7.0 Hz, 2H, *ArH$_2$*), 7.21 – 7.16 (m, 4H, *ArH$_3$+BPhH$_{3'}$*), 6.97 (t, J = 7.4 Hz, 2H, *SePhH$_4$*), 6.92 – 6.87 (m, 2H, *ArH$_5$*), 6.87 – 6.82 (m, 1H, *SePhH$_6$*), 6.79 – 6.68 (m, 3H, *BPhH$_7$*), 0.42 (s, 3H, *SiCH$_3$*), 0.40 (s, 3H, *SiCH$_3$*); ^{13}C NMR (150 MHz, THF-d_8) δ 162.2 (*ArC$_{11}$*), 139.0 (*SePhC$_{10}$*), 134.9 (*SePhC$_1$*), 134.8 (*BPhC$_4$*), 133.6 (*ArC$_2$*), 130.9 (*ArC$_{12}$*), 129.0 (*ArC$_3$*), 127.8 (*BPhC$_9$*), 126.9 (*SePhC$_5$*), 126.8 (*ArC$_6$*), 124.1 (*SePhC$_7$*), 123.4 (*BPhC$_8$*), -1.4 (*SiMe*); ^{29}Si NMR (119 MHz, THF-d_8) δ 7.6 (s); ^{77}Se NMR (114 MHz, THF-d_8) δ 200.0 (s); ^{11}B NMR (193 MHz, THF-d_8) δ -2.1 (s); HRMS (m/z): Calc. For $[M-K]^-$ ($[C_{26}H_{22}BSeSi]^-$): 453.0749 Found: 453.0716.

*Due to coupling with quadrupolar nuclei, the carbon atoms adjacent to boron could not be resolved but could be observed in the HMBC spectrum.

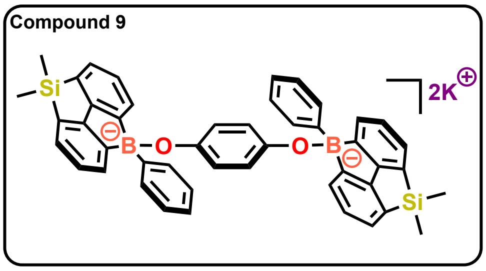

Compound 9. To a THF (0.6 mL) solution of **4** (35.2mg, 0.055 mmol) in a J Young NMR tube, sublimed benzoquinone (3.1 mg, 0.028 mmol) was added in one portion. The reaction was heated at 55 ºC for 12 hours. Afterward, the insoluble precipitate was filtered. Then, all the volatiles were removed under vacuum, and the residue was extracted with benzene. After evaporation of the solvent in vacuo, the residue was washed with *n*-hexane (3 × 4 mL), resulting in an off-white solid corresponding to **9** (18 mg, 41%). Single crystals suitable for X-ray diffraction analysis were obtained by slow diffusion of Et_2O into a THF solution of **9** with 2 equiv of 2,2,2-Cryptand.

^{1}H NMR (600 MHz, C_6D_6) δ 7.86 – 7.77 (m, 4H, *BPhH₁*), 7.74 – 7.65 (m, 4H, *ArH₂*), 7.33 (d, J = 7.0 Hz, 4H, *ArH₃*), 7.19 (t, J = 6.9 Hz, 4H, *ArH₄*), 7.06 (t, J = 7.5 Hz, 4H, *BPhH₅*), 6.93 (t, J = 7.3 Hz, 2H, *BPhH₆*), 6.29 (s, 4H, *OPhH₇*), 0.46 (s, 6H, *SiCH₃*), 0.30 (s, 9H, *SiCH₃*, *ov*); ^{13}C NMR (150 MHz, C_6D_6) δ 161.8 (*ArC₈*), 155.2 (*OPhC₁₀*), 134.1 (*ArC₂*), 133.4 (*BPhC₁*), 131.5 (*ArC₉*), 130.3 (*ArC₃*), 128.3 (*BPhC₅*), 127.5 (*ArC₄*), 126.0 (*BPhC₆*), 122.3 (*OPhC₇*), -1.3 (*SiMe*), -2.3 (*SiMe*); ^{29}Si NMR (119 MHz, C_6D_6) δ 7.7 (s); ^{11}B NMR (193 MHz, C_6D_6) δ 6.8 (s); HRMS (m/z): Calc. For $[M+H]^+$ ($[C_{46}H_{39}B_2K_2O_2Si_2]^+$): 779.1959 Found: 779.1901.

*Due to coupling with quadrupolar nuclei, the carbon atoms adjacent to boron could not be resolved but could be observed in the HMBC spectrum.

Table S1. Substrates tested for reactions with compound **4** and their results.

Substrates	Crystal isolated	Starting material consumed, but no isolable product obtained	No reaction observed	Remarks
O_2	√			*This work*
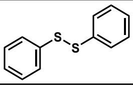	√			*This work*
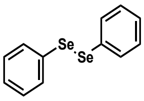	√			*This work*
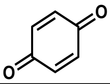	√			*This work*
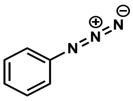		√		Complicated mixture
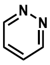			√	
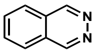			√	
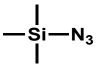			√	
NaN_3			√	
MeCN			√	
$PhNHNH_2$			√	
$PhNH_2$			√	
AIBN			√	
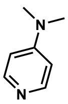			√	
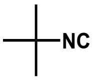			√	
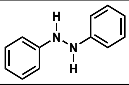			√	
CH_3NCS			√	
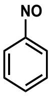		√		
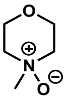		√		

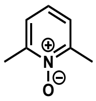			√	
TEMPO			√	
$NOBF_4$		√		
NMe_3·HCl/NEt_3·HCl		√		Compound 3 recovered
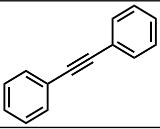			√	
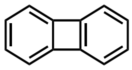			√	
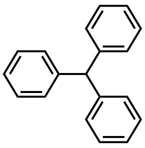			√	
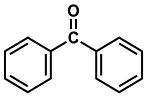			√	
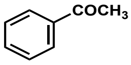			√	
CH_3COCH_3			√	
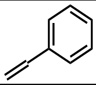			√	
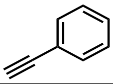			√	
Ph_3CCl			√	
PhCHO		√		
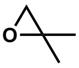		√		Complicated mixture
PhX, X = F, Br, I		√		Complicated mixture
CH_2Cl_2		√		Compound **3** recovered
CH_3I		√		Compound **3** recovered
HOTf		√		Compound **3** recovered
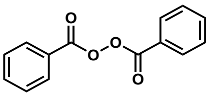		√		Compound **3** recovered
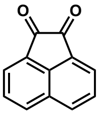			√	

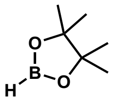			√	
$BH_3{\cdot}THF$		√		Complicated mixture
$Br_4B_2{\cdot}SMe_2$		√		Complicated mixture
CS_2		√		Compound **3** recovered
GaI			√	
CaI_2			√	
N≡N			√	
CO_2		√		Compound **3** recovered
S_8		√		Complicated mixture
Se (red)		√		Complicated mixture
C_{60}			√	
$PdCl_2$			√	
$Ni(COD)_2$			√	
$(PPh_3)AuCl$		√		gold mirror observed
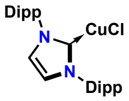		√		Complicated mixture
$W(CO)_6$			√	
Bu_3SnH		√		
$(Et)_3PO$			√	
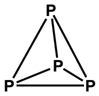			√	
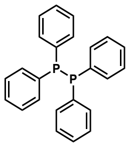			√	
**$(Bu)_3P{=}Te$**			√	

2. NMR Spectra

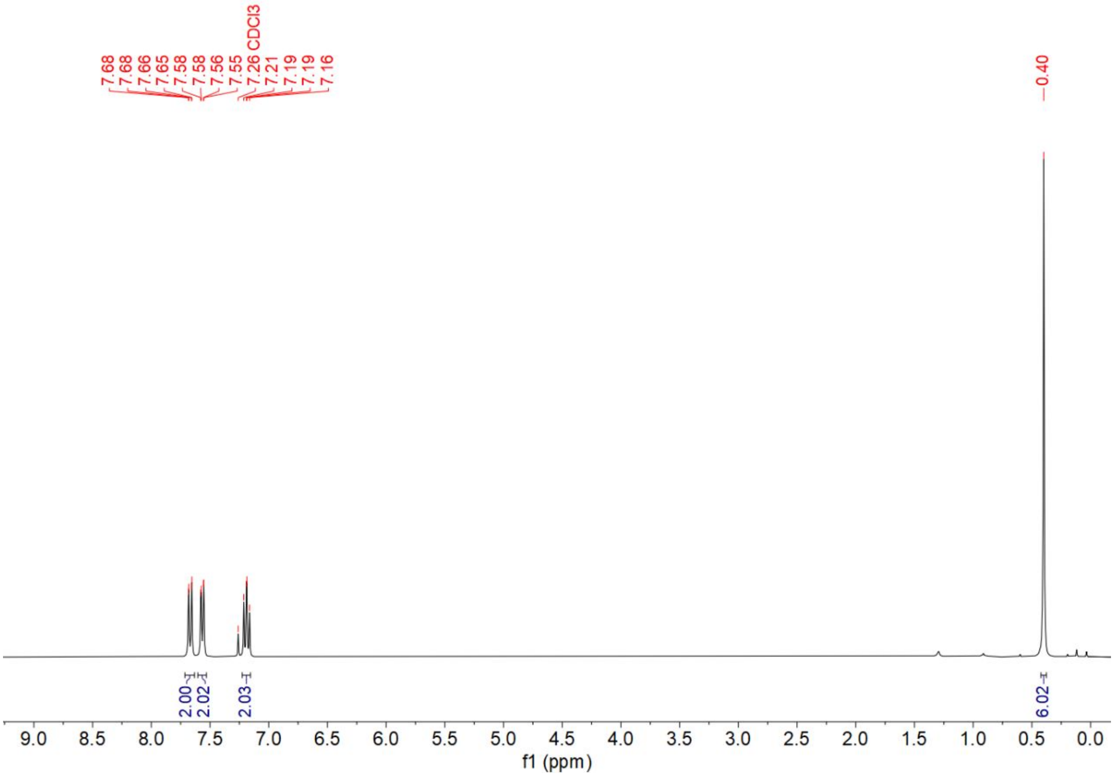

Figure S1. ^{1}H NMR (300.15 MHz) spectrum of compound **1** in $CDCl_3$.

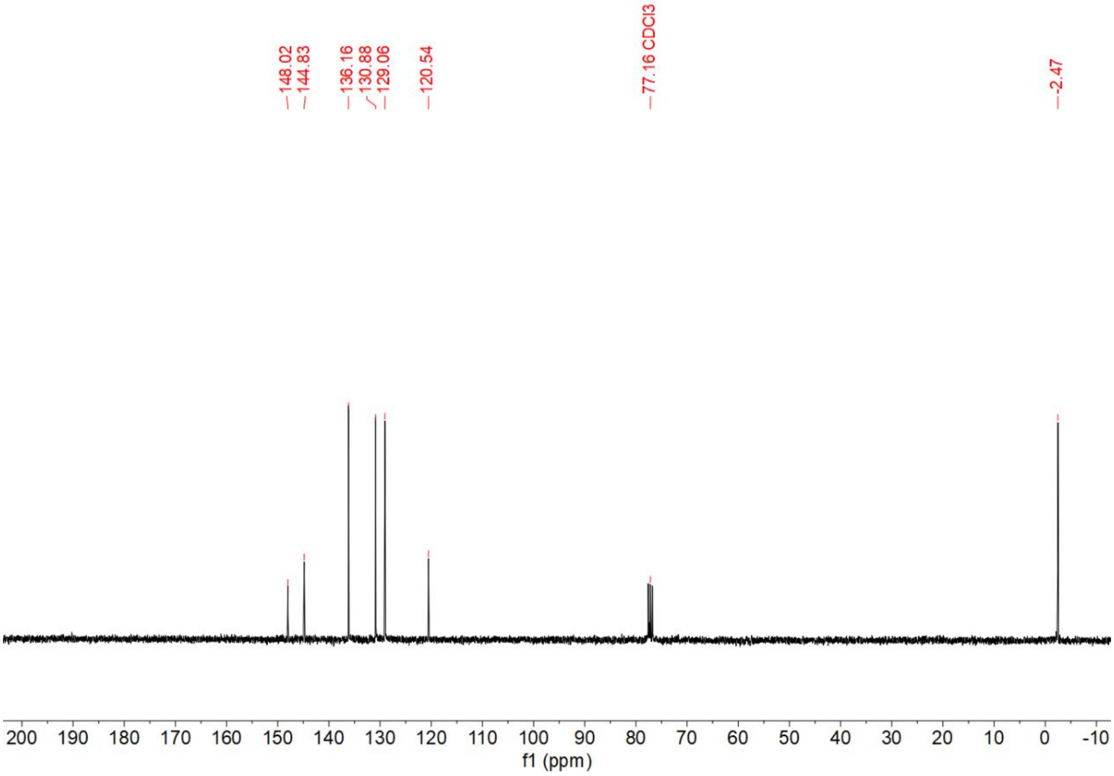

Figure S2. $^{13}C\{^{1}H\}$ NMR (75.48 MHz) spectrum of compound **1** in $CDCl_3$.

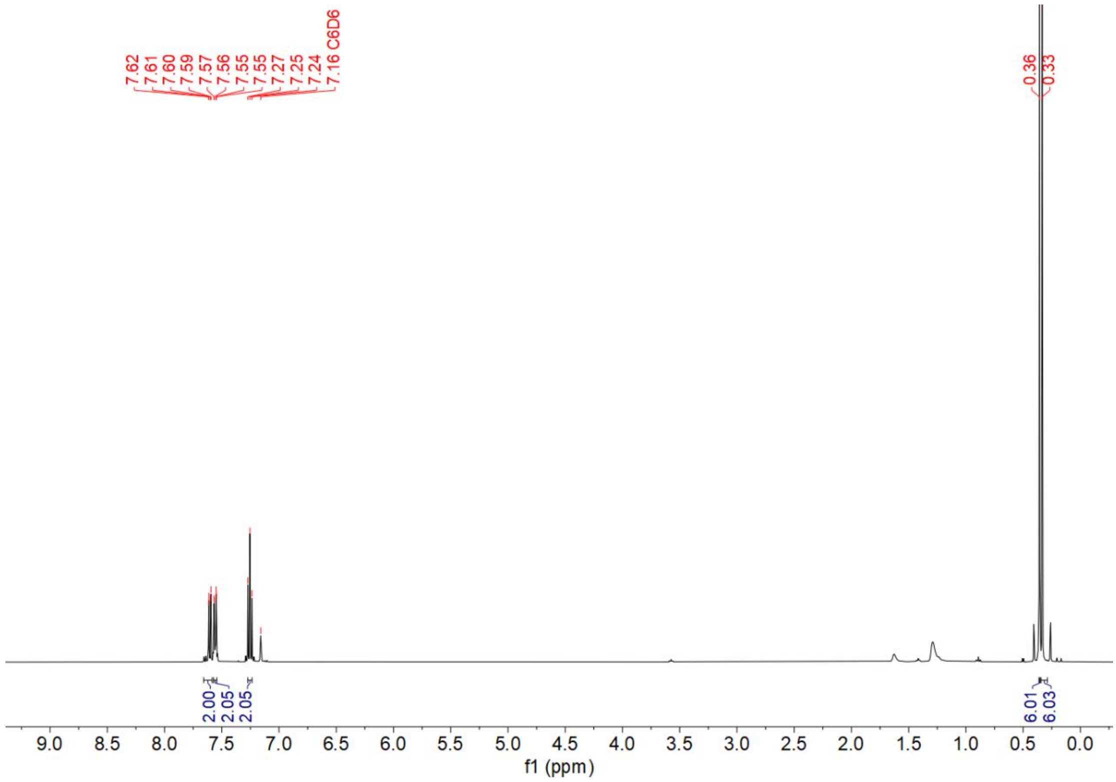

Figure S3. ^{1}H NMR (400.13 MHz) spectrum of compound **2** in C_6D_6.

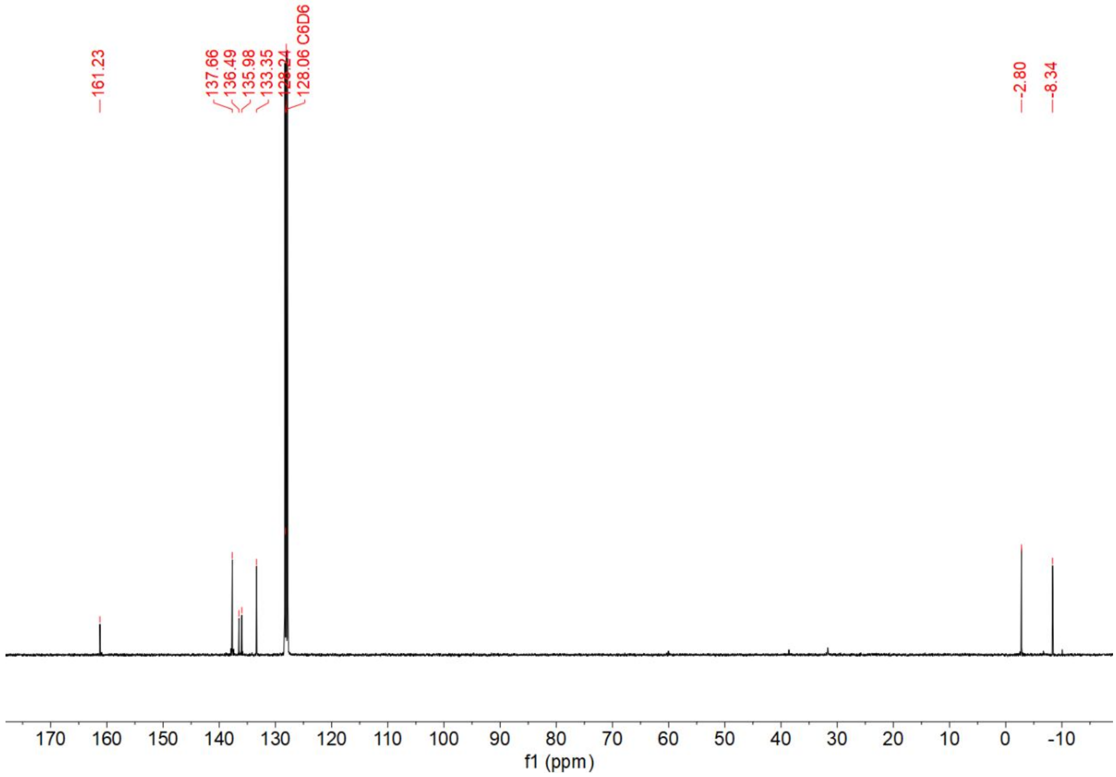

Figure S4. $^{13}C\{^{1}H\}$ NMR (100.62 MHz) spectrum of compound **2** in C_6D_6.

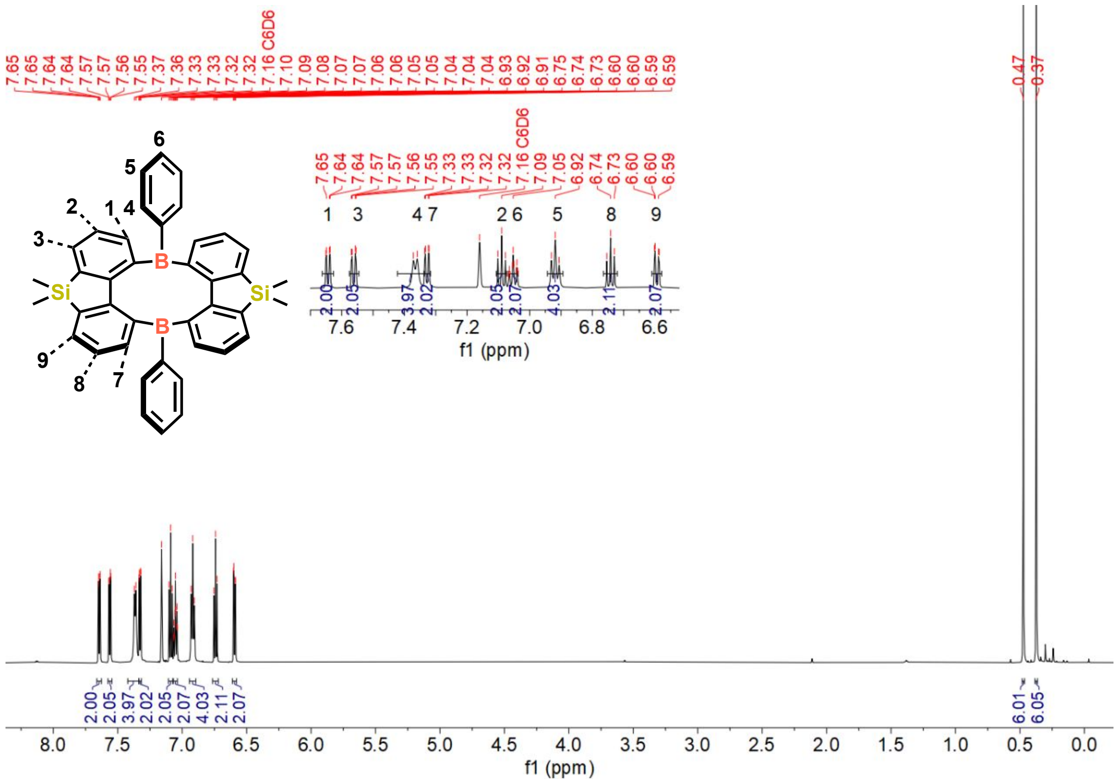

Figure S5. ^{1}H NMR (600.17 MHz) spectrum of compound **3** in C_6D_6.

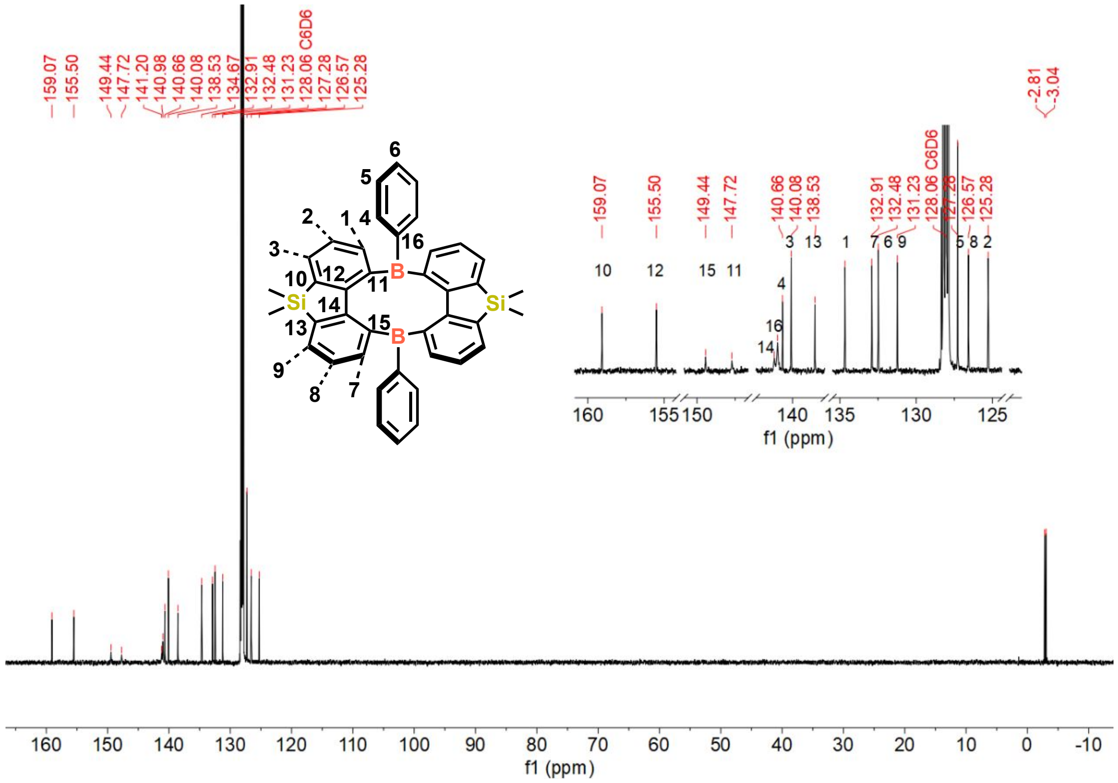

Figure S6. $^{13}C\{^{1}H\}$ NMR (150.93 MHz) spectrum of compound **3** in C_6D_6.

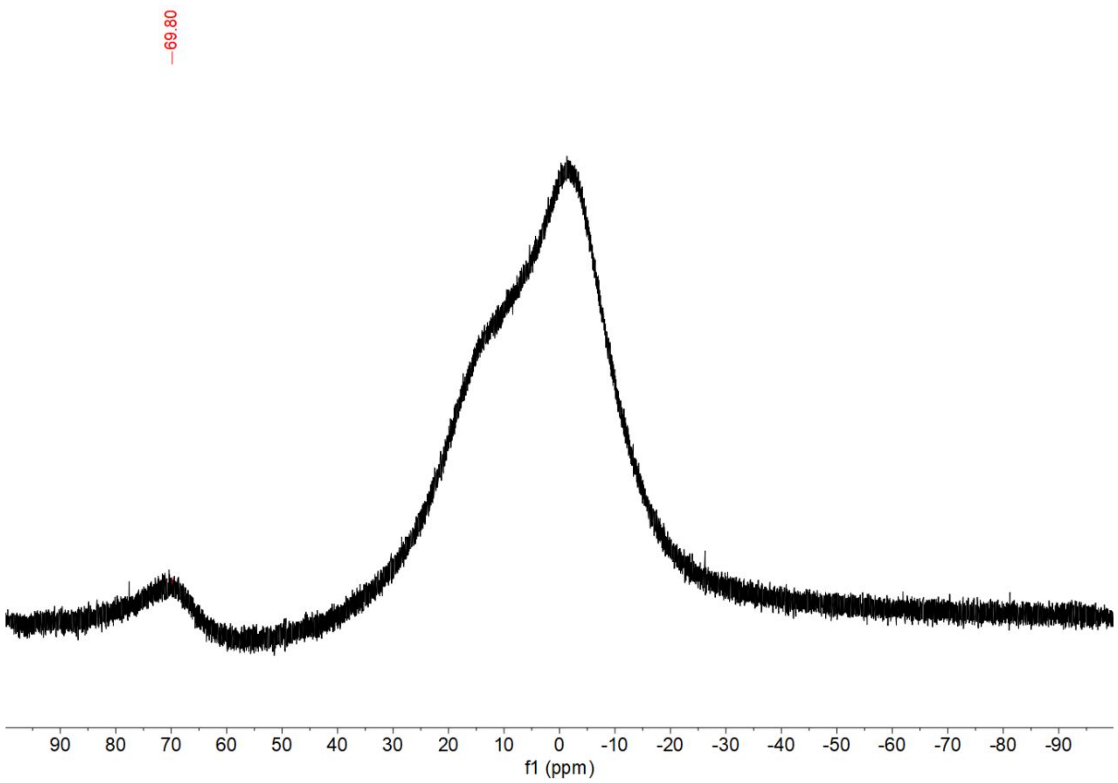

Figure S7. ^{11}B NMR (192.56 MHz) spectrum of compound **3** in C_6D_6.

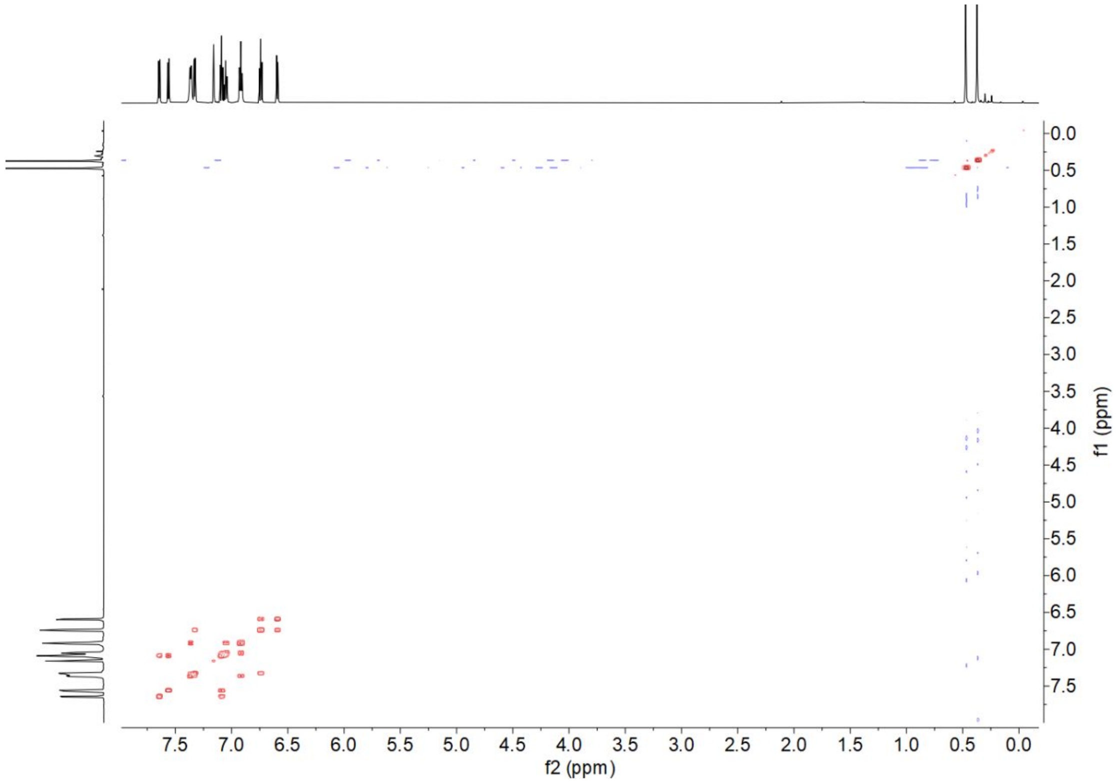

Figure S8. ^1H-^1H COSY NMR (600.17, 600.17 MHz) spectrum of compound **3** in C_6D_6.

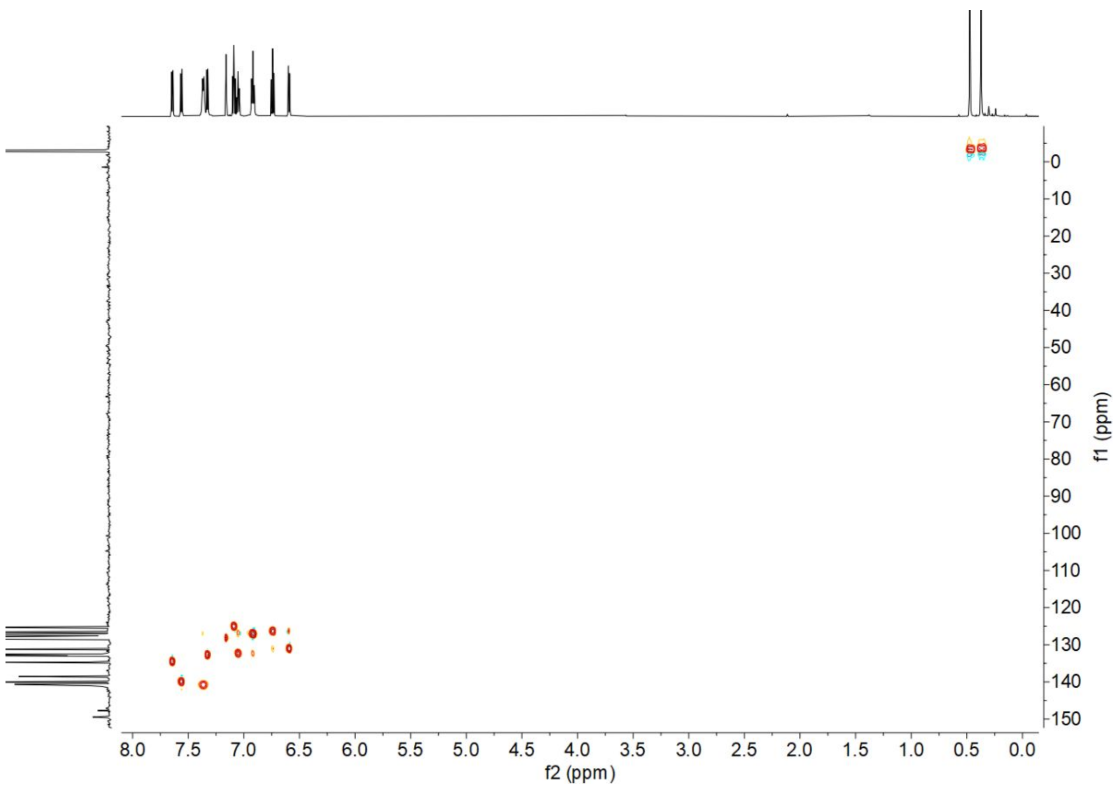

Figure S9. ^{1}H-^{13}C HSQC NMR (600.17, 150.91 MHz) spectrum of compound **3** in C_6D_6.

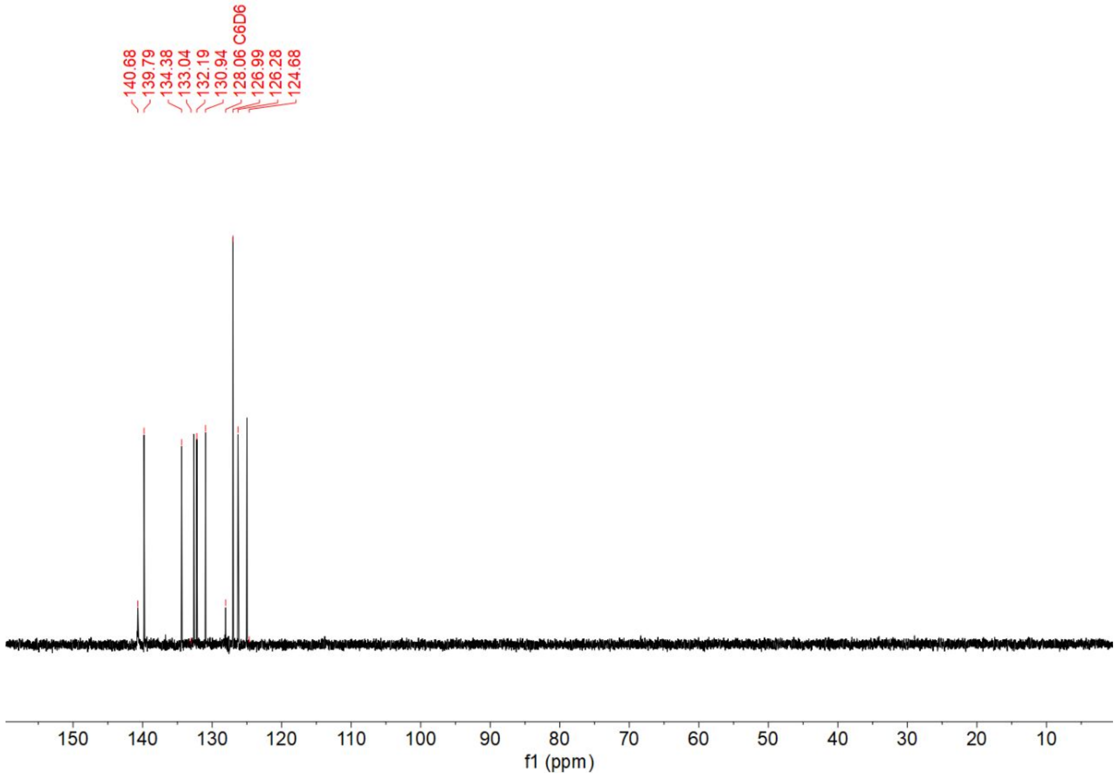

Figure S10. ^{13}C DEPT135 NMR (150.92 MHz) spectrum of compound **3** in C_6D_6.

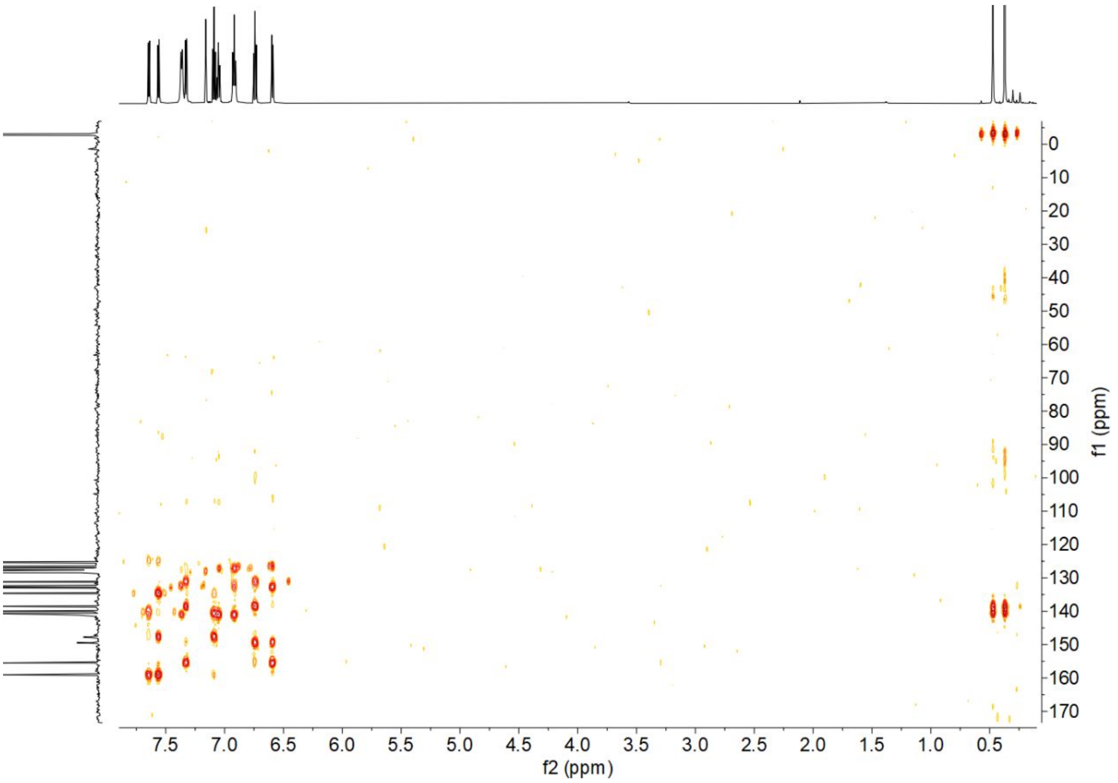

Figure S11. ^{1}H-^{13}C HMBC NMR (600.17, 150.91 MHz) spectrum of compound **3** in C_6D_6.

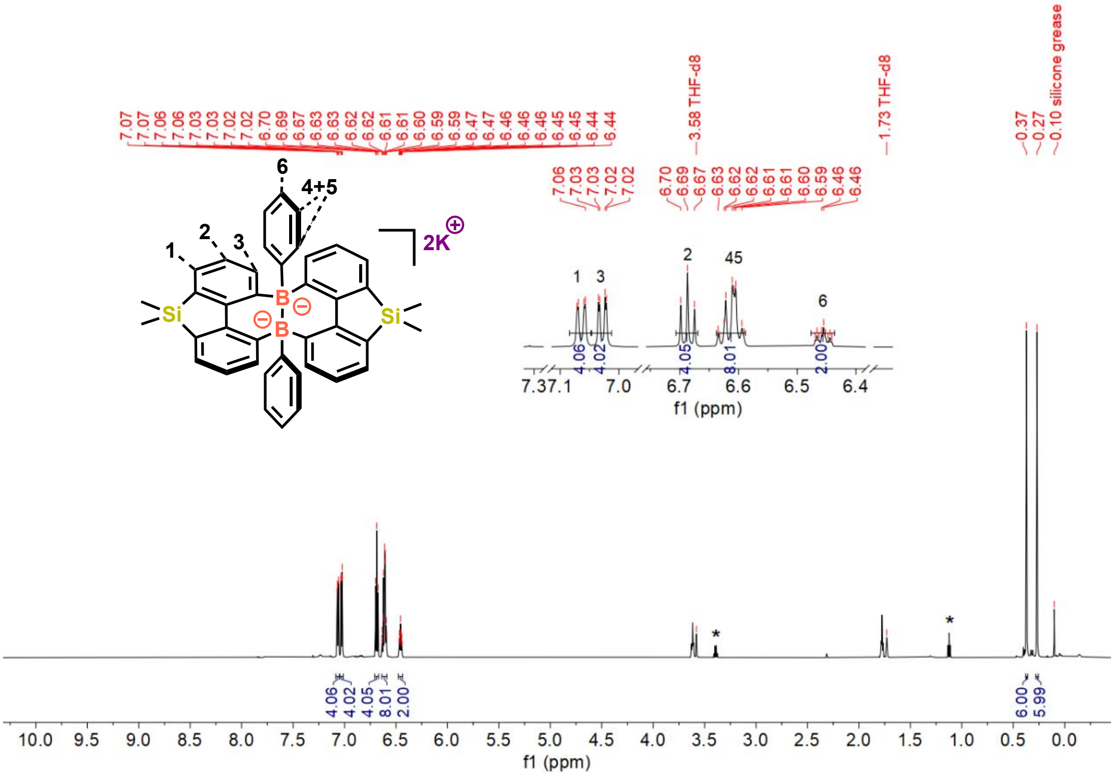

Figure S12. ^{1}H NMR (600.17 MHz) spectrum of compound **5** in THF-d_8.

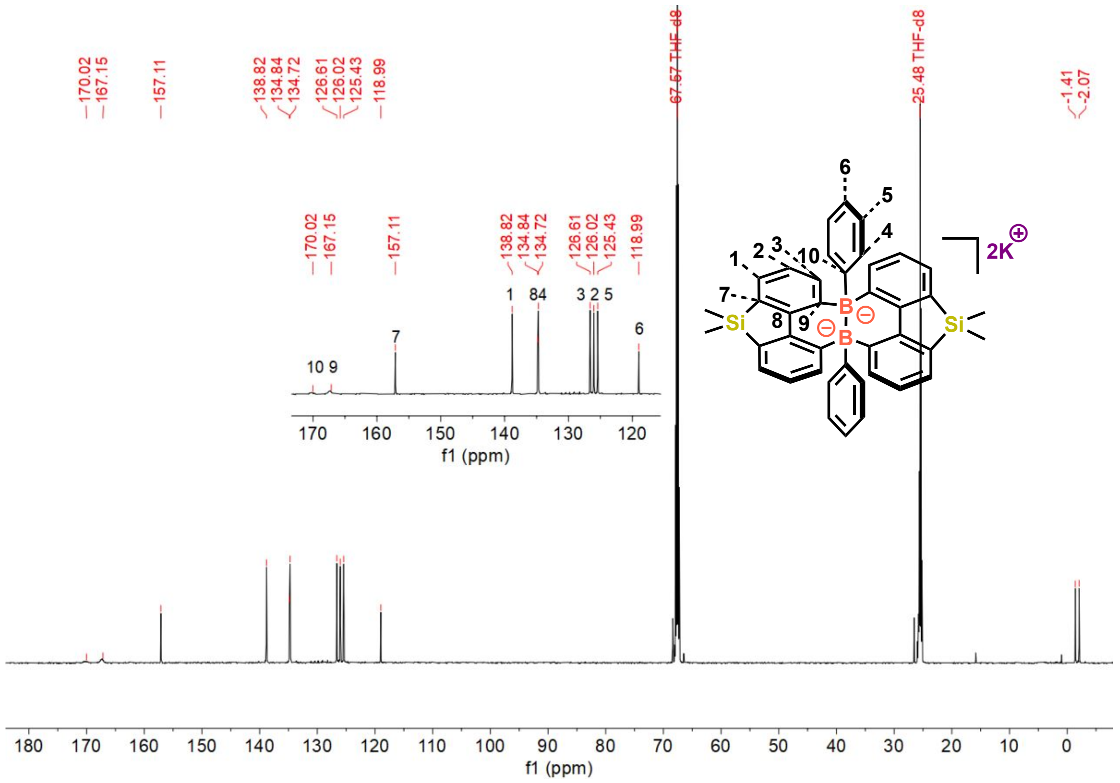

Figure S13. $^{13}C\{^{1}H\}$ NMR (150.93 MHz) spectrum of **5** in THF-d_8.

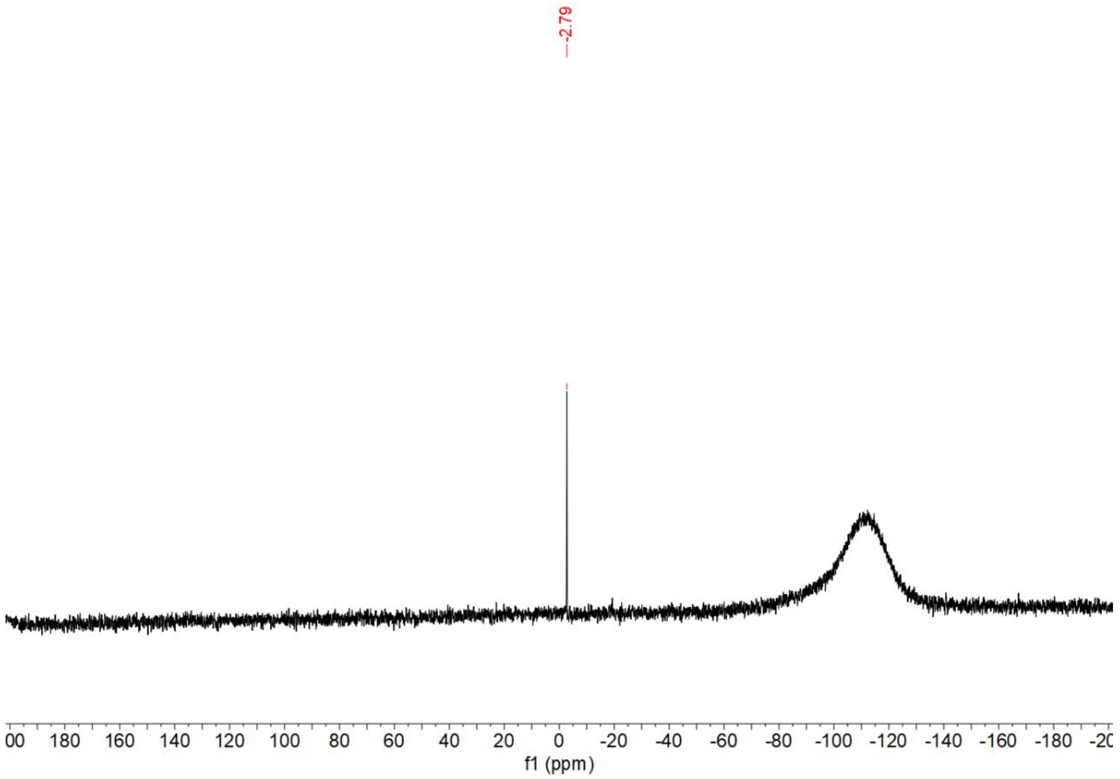

Figure S14. $^{29}Si\{^{1}H\}$ NMR (119.24 MHz) spectrum of **5** in THF-d_8.

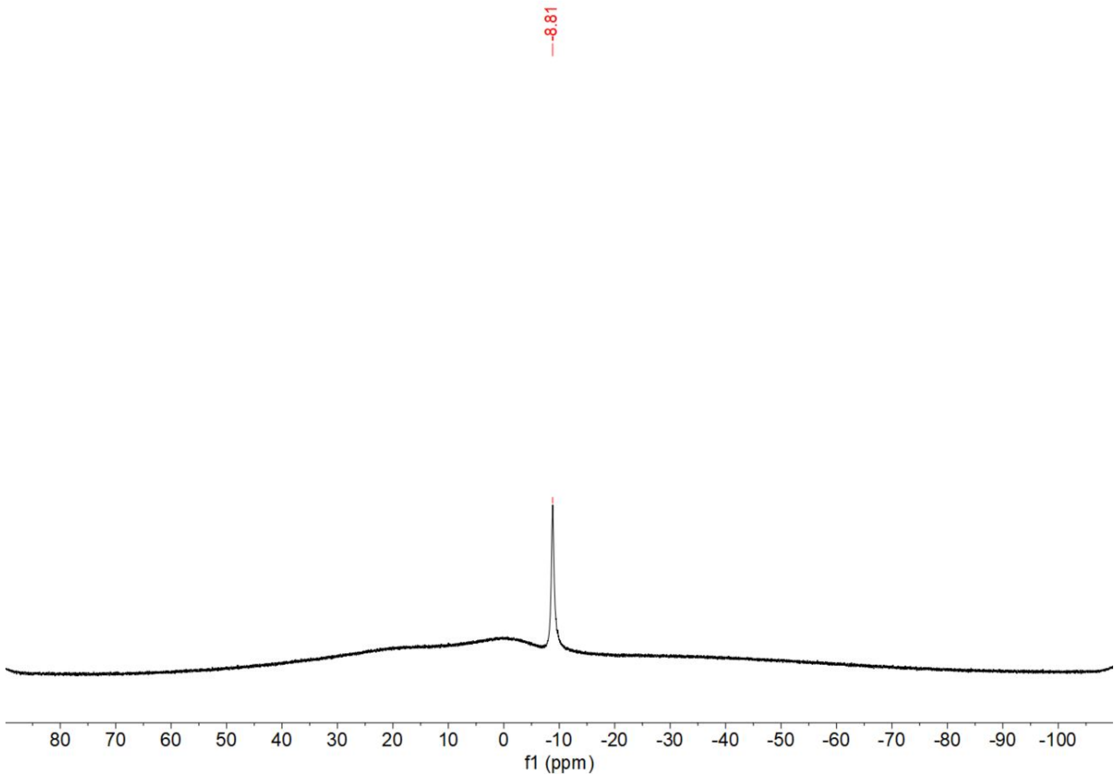

Figure S15. ^{11}B NMR (192.56 MHz) spectrum of **5** in THF-d_8.

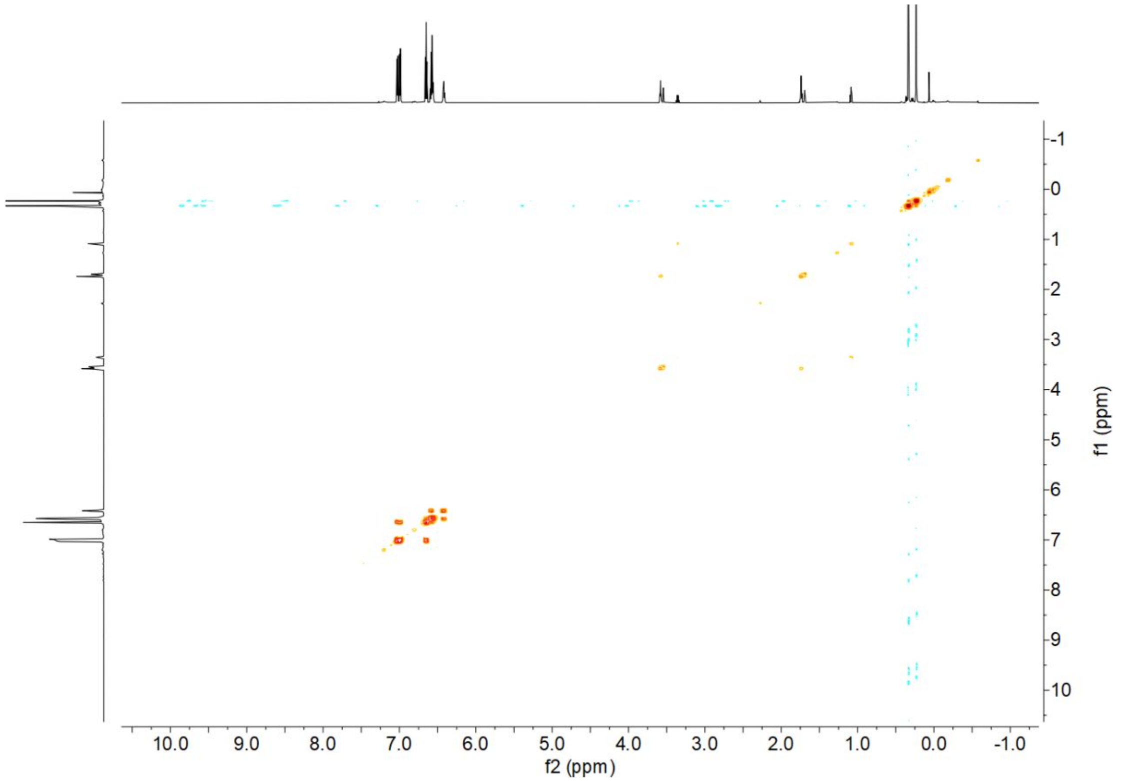

Figure S16. ^{1}H-^{1}H COSY NMR (600.17, 600.17 MHz) spectrum of **5** in THF-d_8.

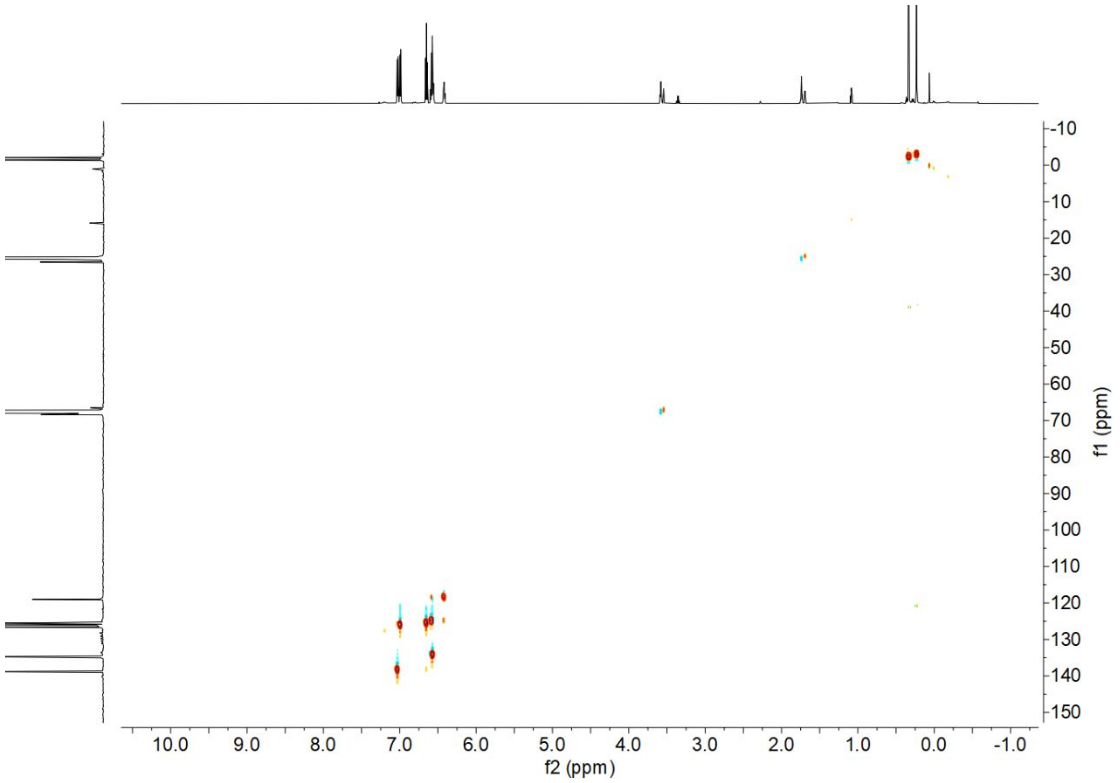

Figure S17. ^{1}H-^{13}C HSQC NMR (600.17, 150.91 MHz) spectrum of **5** in THF-d_8.

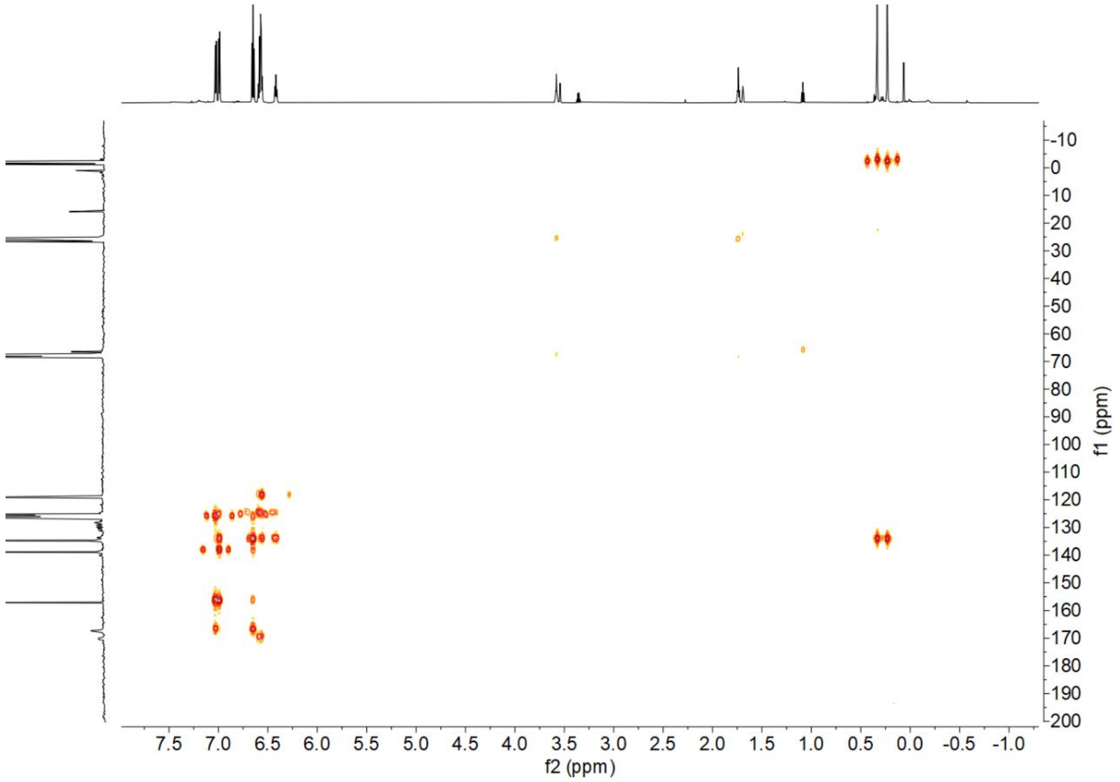

Figure S18. ^{1}H-^{13}C HMBC NMR (600.17, 150.91 MHz) spectrum of **5** in THF-d_8.

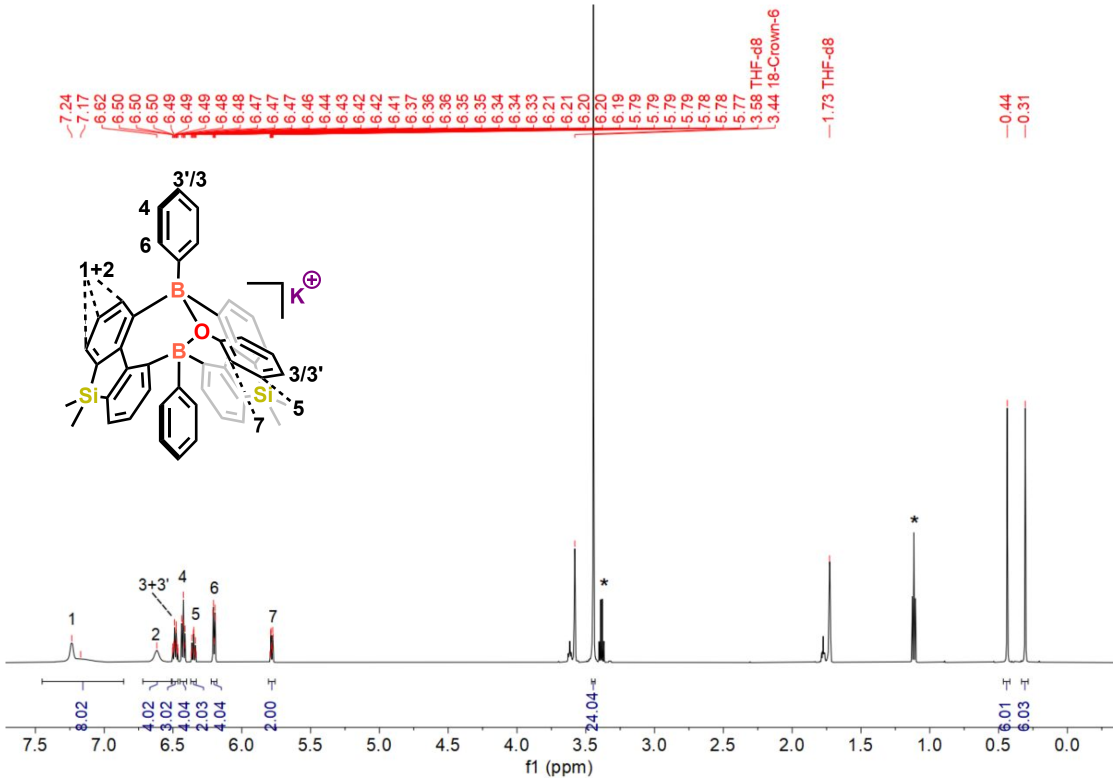

Figure S19. ^{1}H NMR (600.17 MHz) spectrum of compound **6•18-crown-6** in THF-d_8.

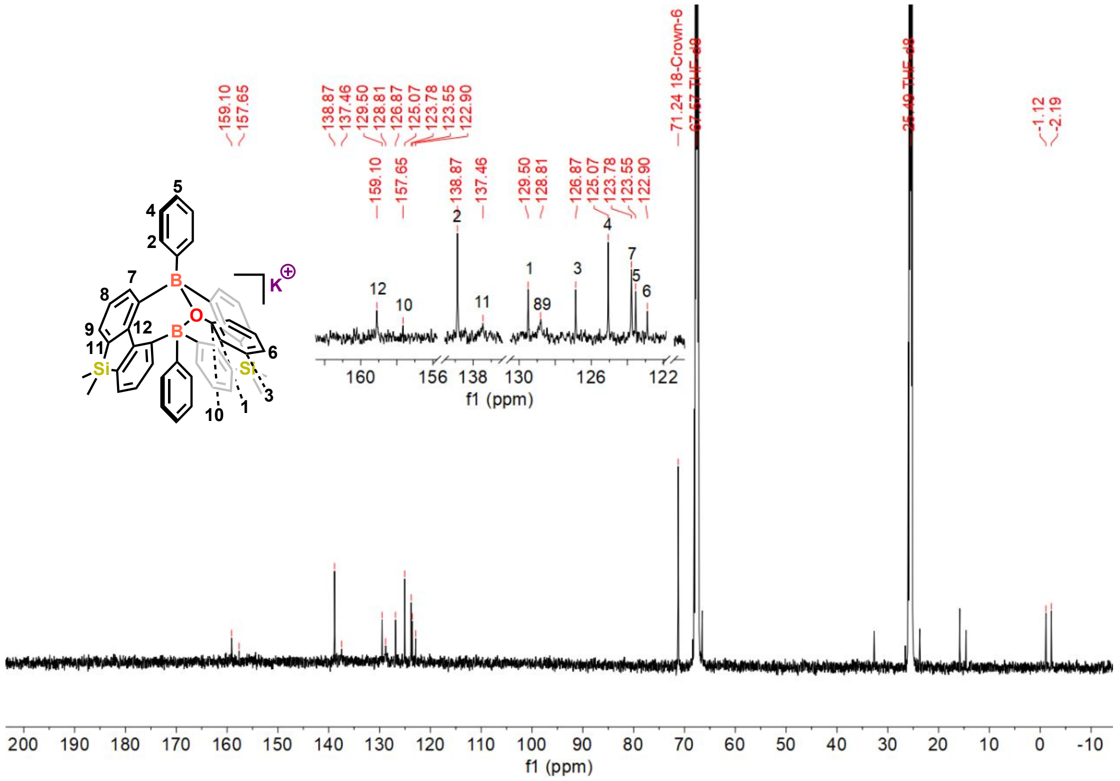

Figure S20. $^{13}C\{^{1}H\}$ NMR (150.93 MHz) spectrum of **6•18-crown-6** in THF-d_8.

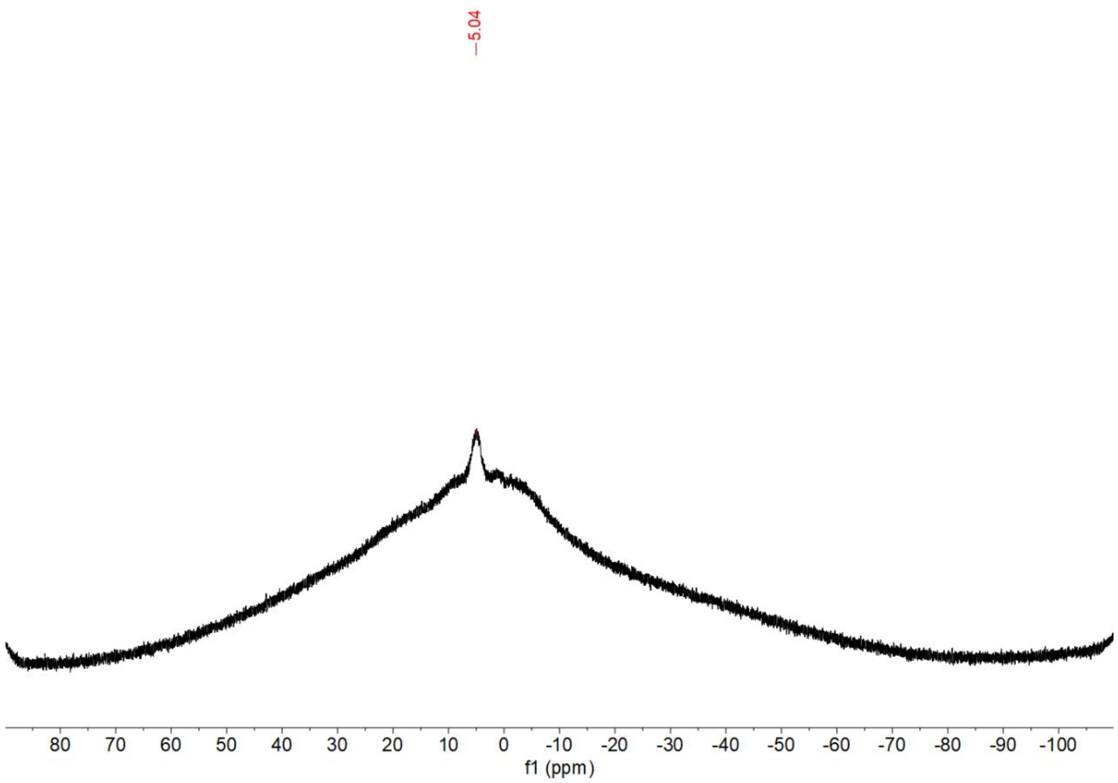

Figure S21. ^{11}B NMR (192.56 MHz) spectrum of **6•18-crown-6** in THF-d_8.

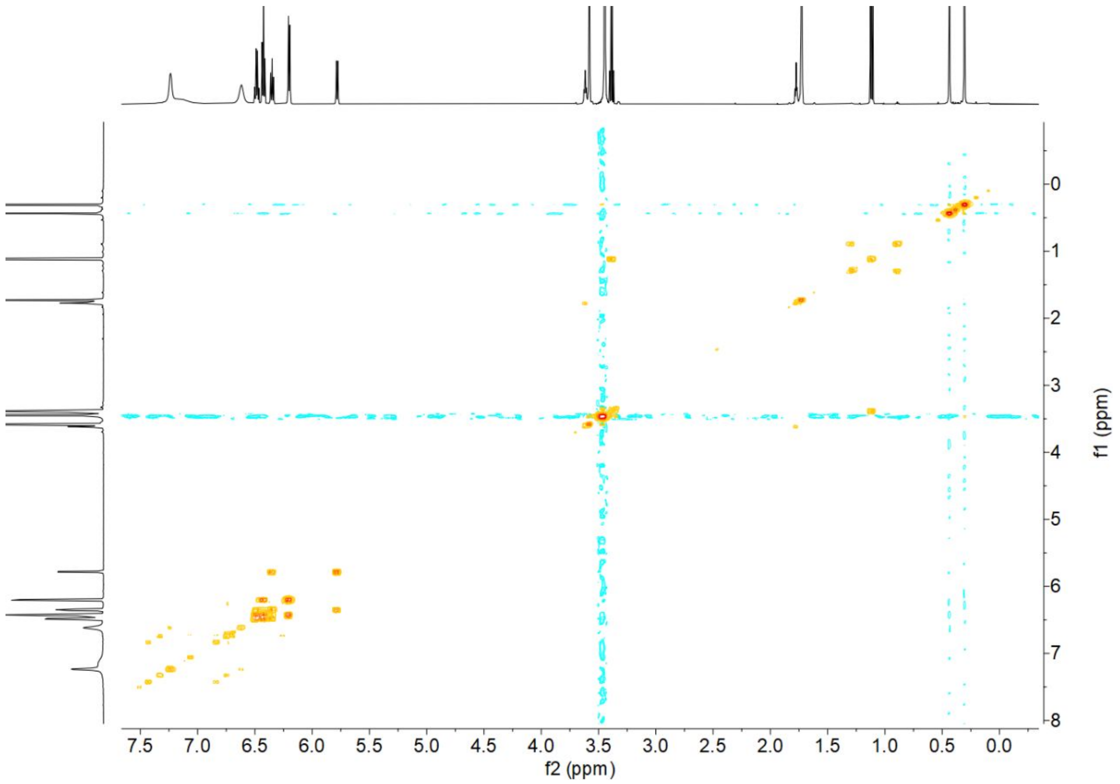

Figure S22. ^{1}H-^{1}H COSY NMR (600.17, 600.17 MHz) spectrum of **6•18-crown-6** in THF-d_8.

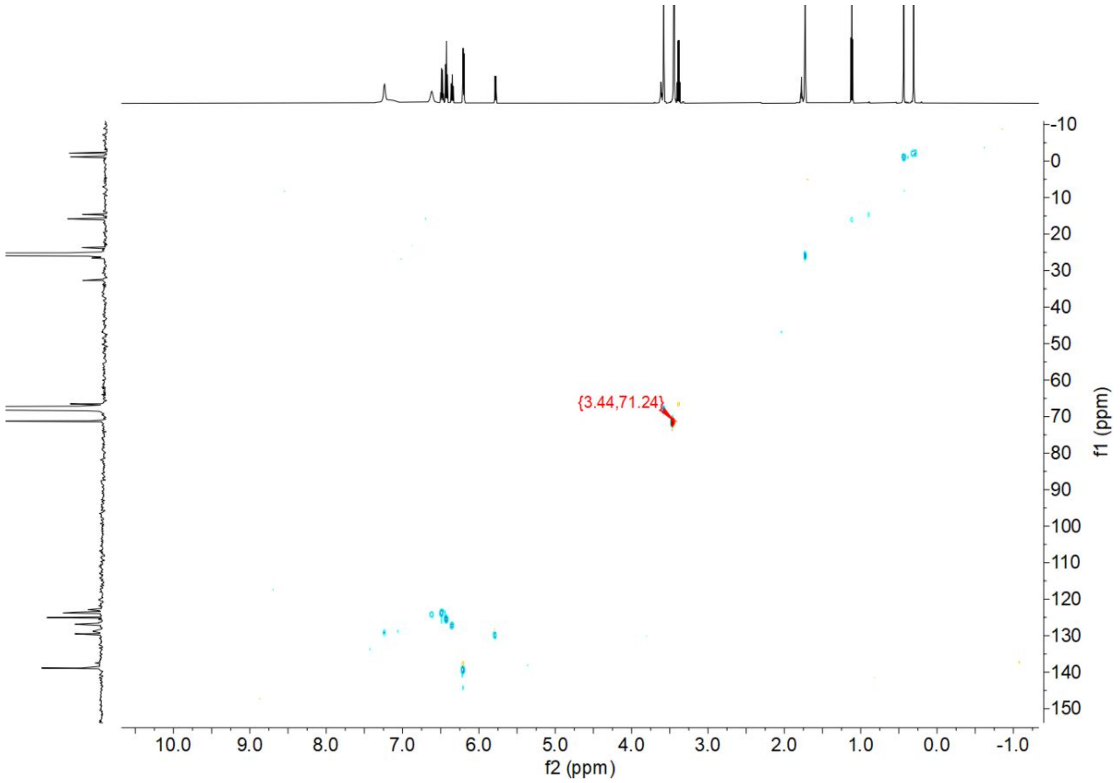

Figure S23. ^{1}H-^{13}C HSQC NMR (600.17, 150.91 MHz) spectrum of **6•18-crown-6** in THF-d_8.

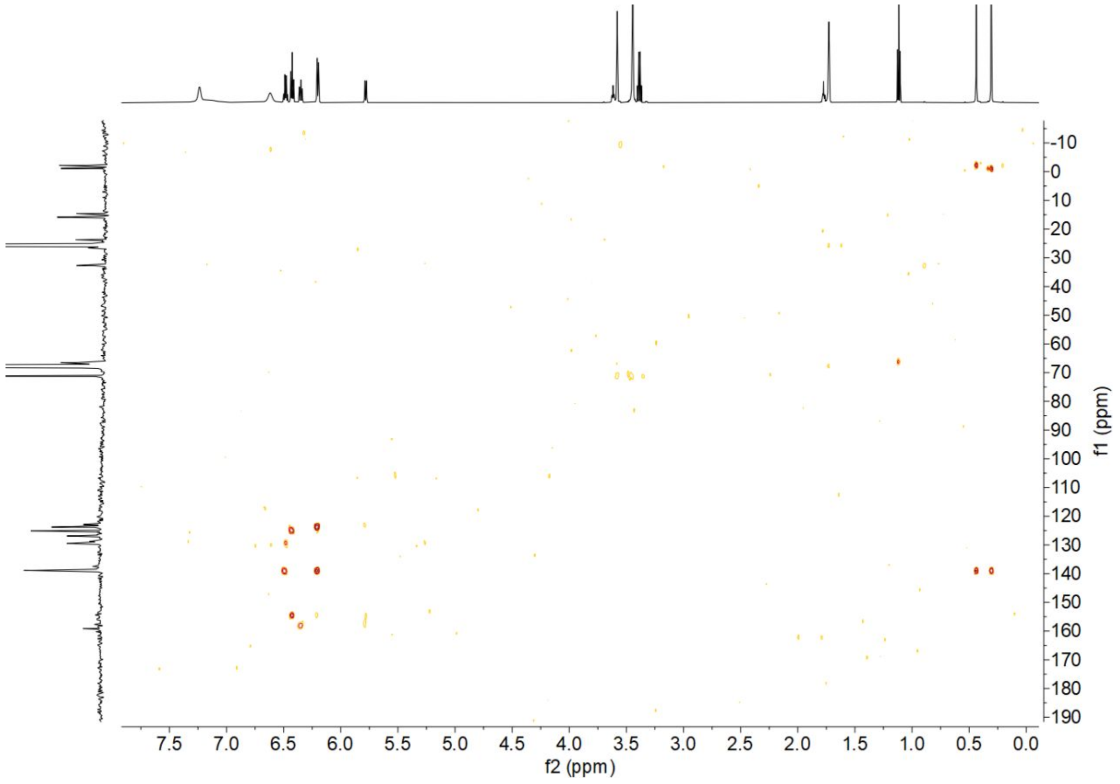

Figure S24. ^{1}H-^{13}C HMBC NMR (600.17, 150.91 MHz) spectrum of **6•18-crown-6** in THF-d_8.

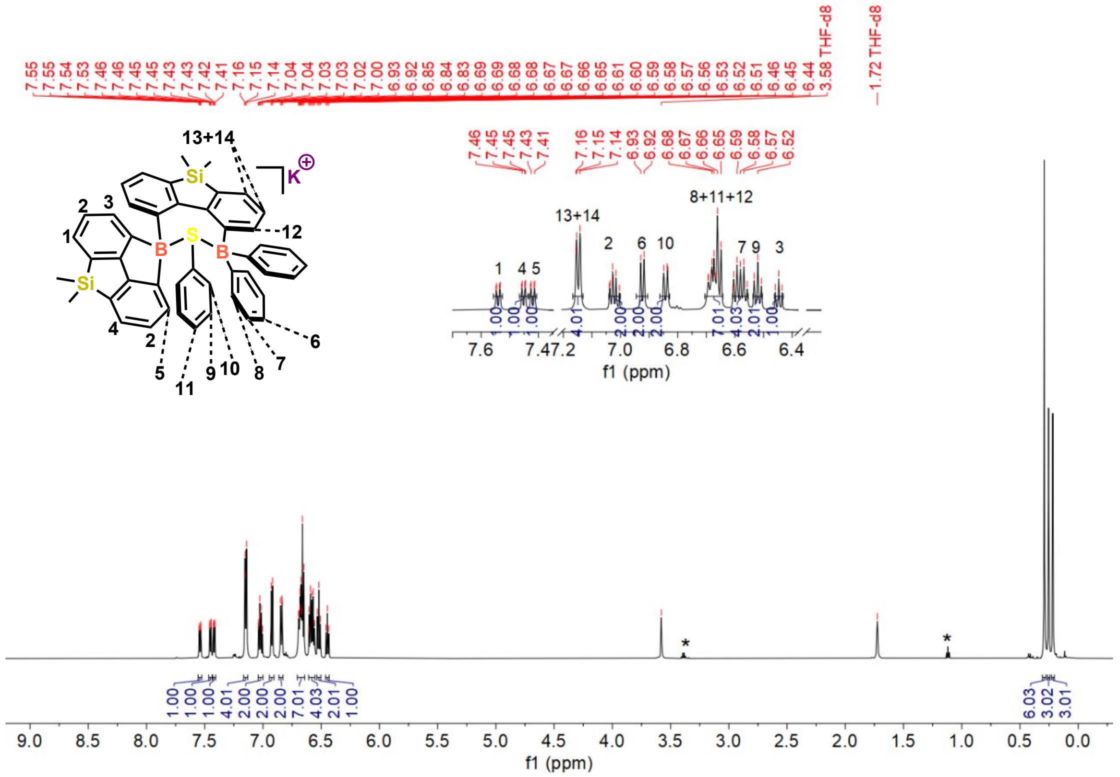

Figure S25. ^{1}H NMR (600.17 MHz) spectrum of compound **7** in THF-d_8.

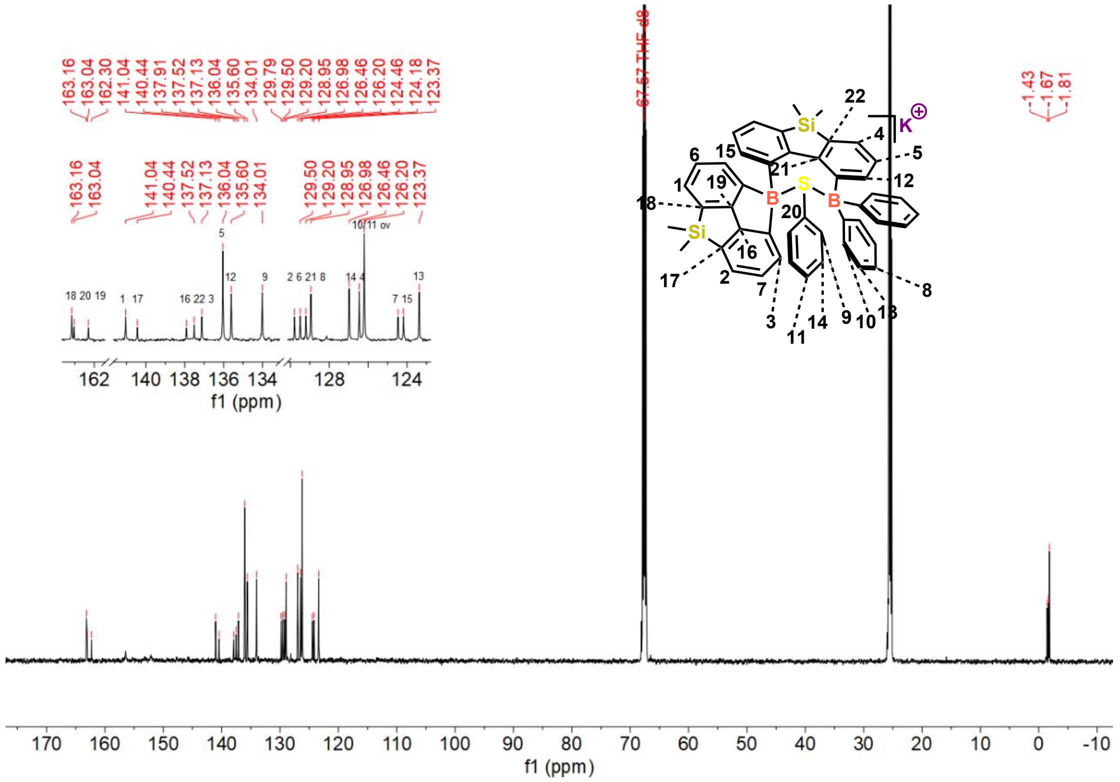

Figure S26. $^{13}C\{^{1}H\}$ NMR (150.93 MHz) spectrum of compound **7** in THF-d_8.

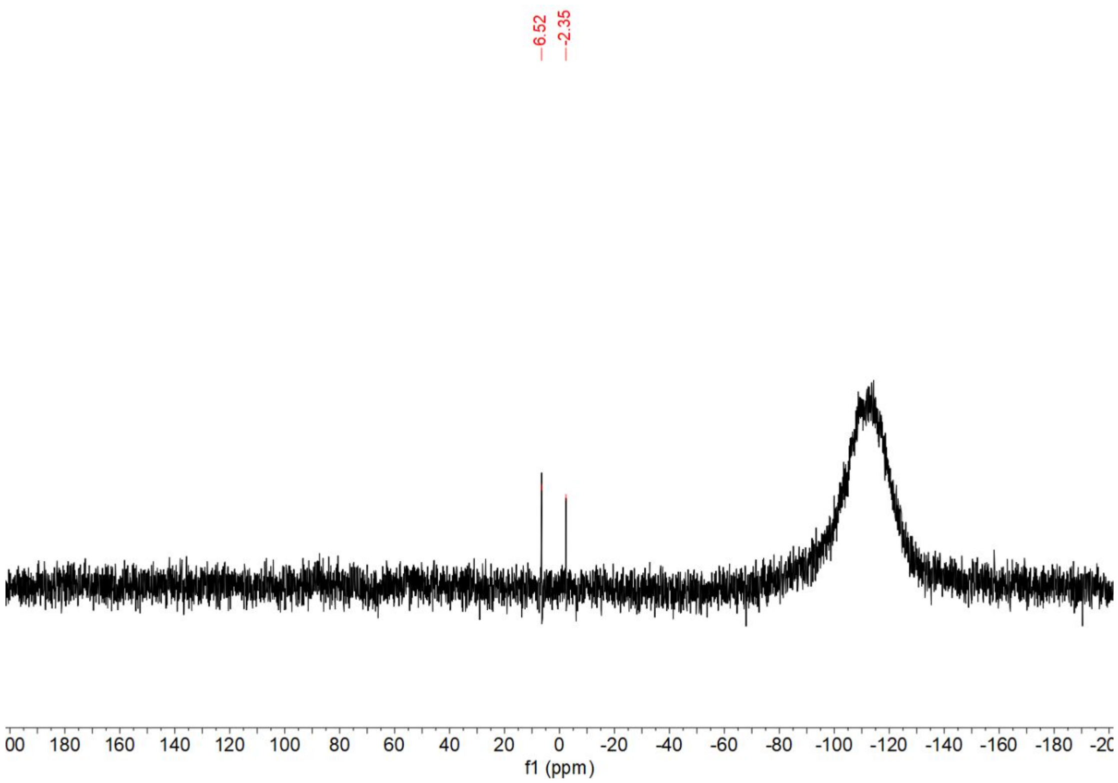

Figure S27. $^{29}Si\{^1H\}$ NMR (119.24 MHz) spectrum of compound **7** in THF-d_8.

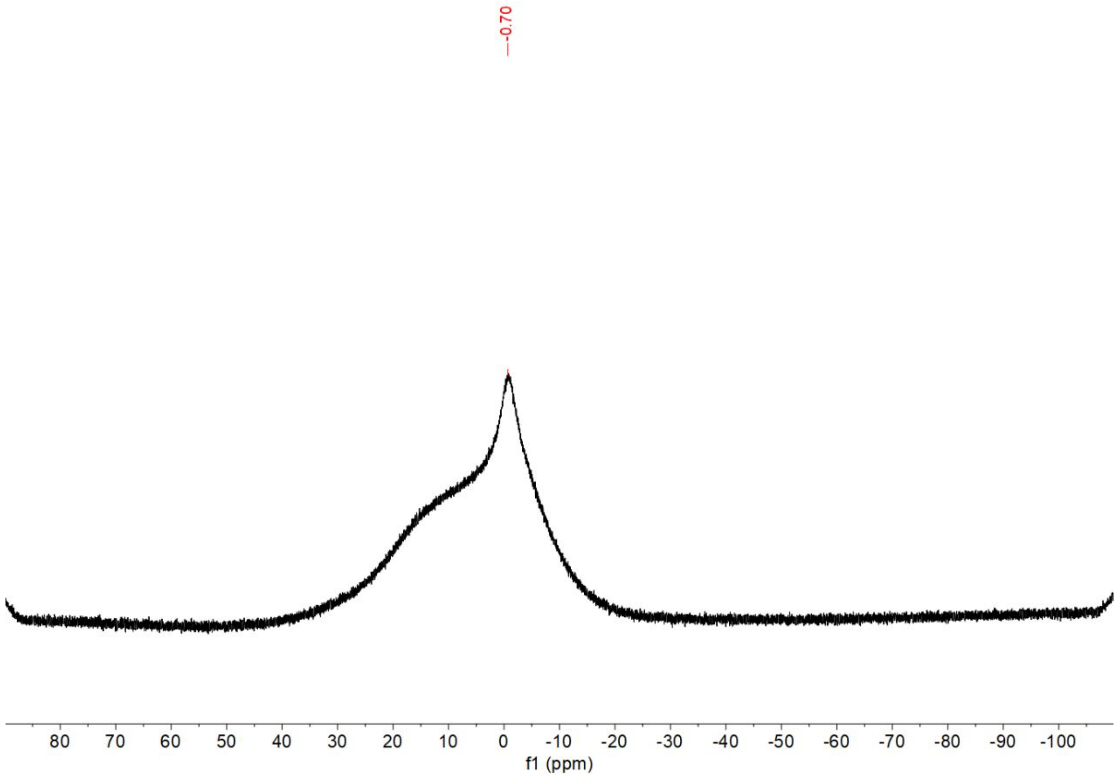

Figure S28. ^{11}B NMR (192.56 MHz) spectrum of compound **7** in THF-d_8.

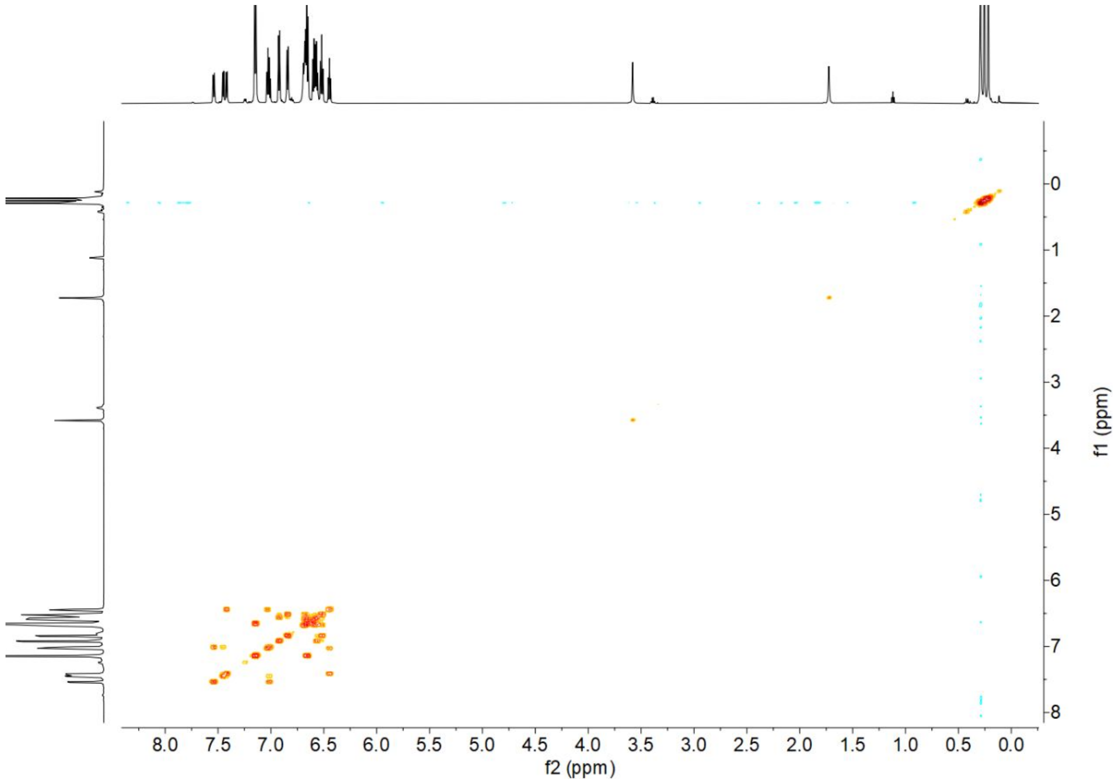

Figure S29. ^{1}H-^{1}H COSY NMR (600.17, 600.17 MHz) spectrum of **7** in THF-d_8.

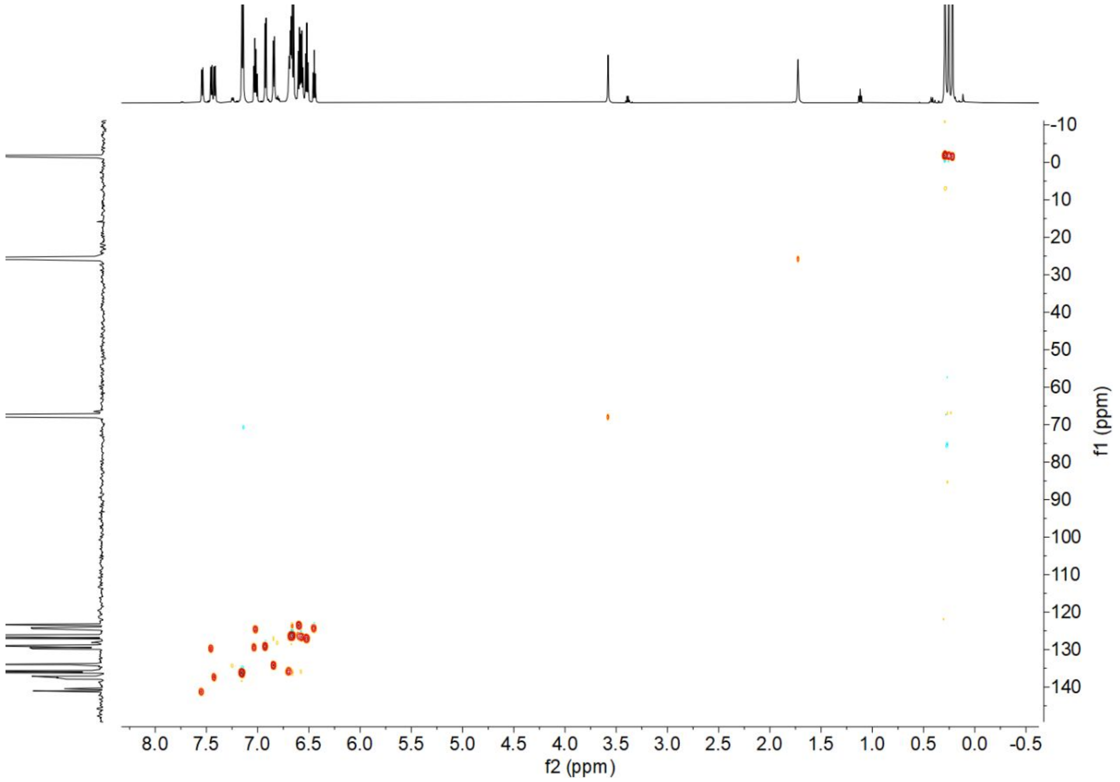

Figure S30. ^{1}H-^{13}C HSQC NMR (600.17, 150.91 MHz) spectrum of **7** in THF-d_8.

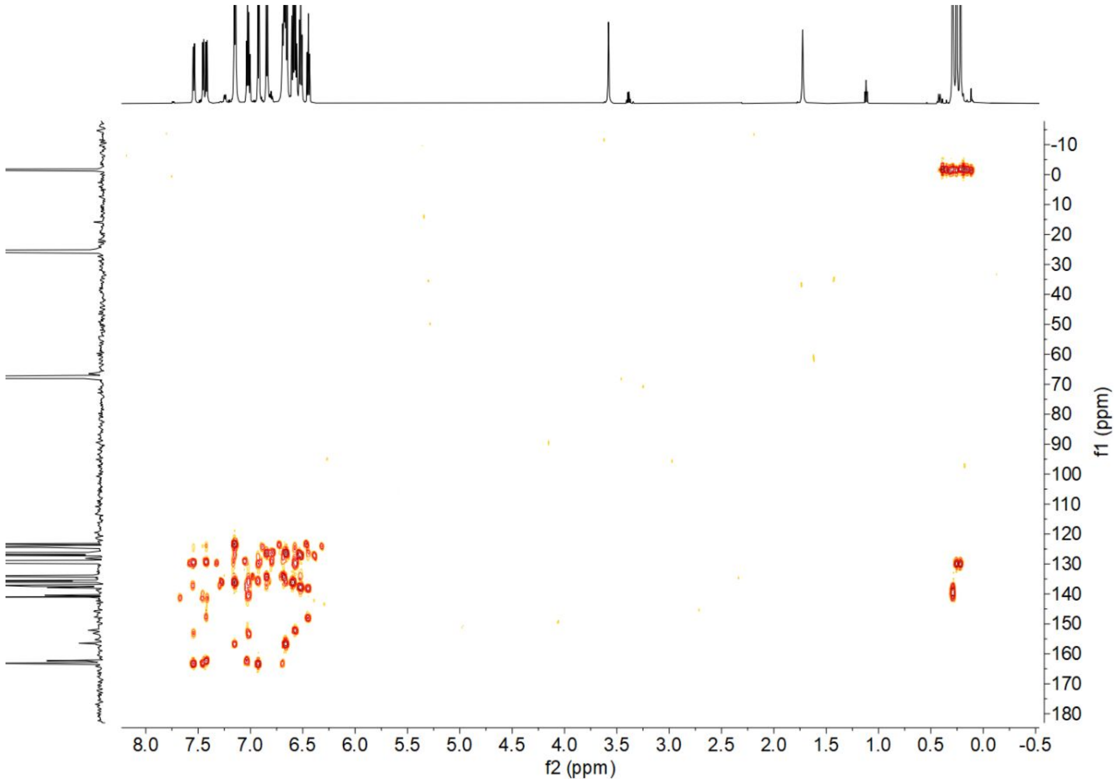

Figure S31. ^{1}H-^{13}C HMBC NMR (600.17, 150.91 MHz) spectrum of **7** in THF-d_8.

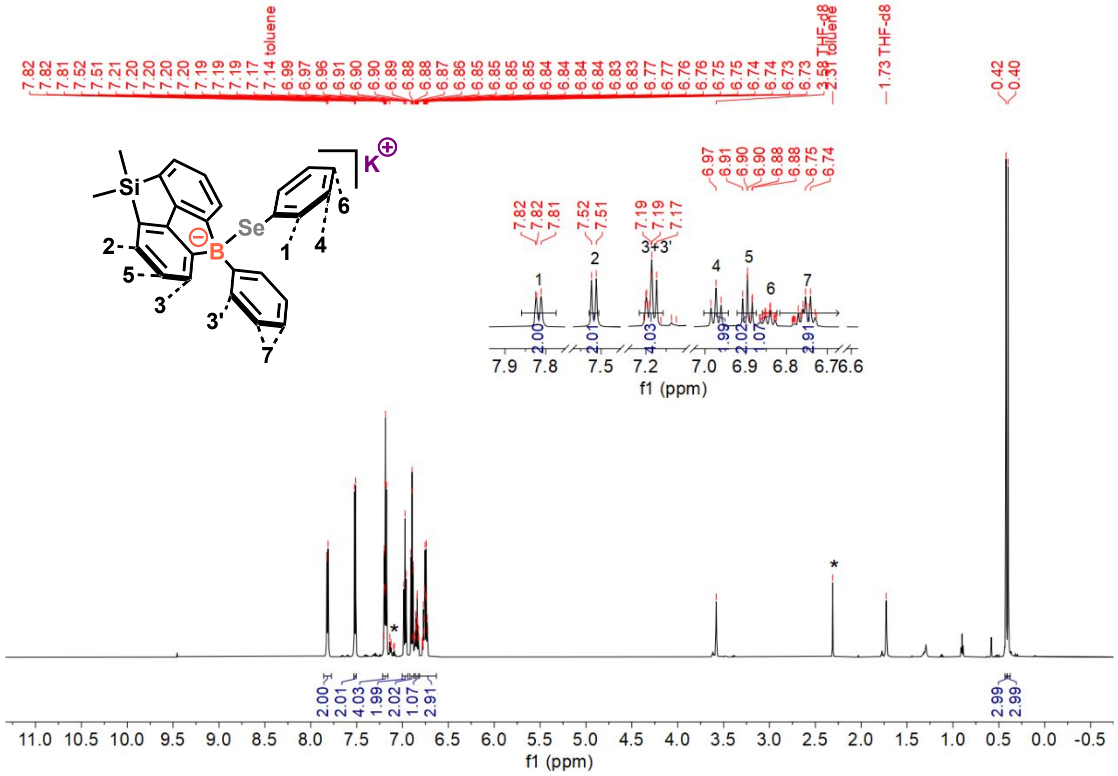

Figure S32. ^{1}H NMR (600.17 MHz) spectrum of compound **8** in THF-d_8.

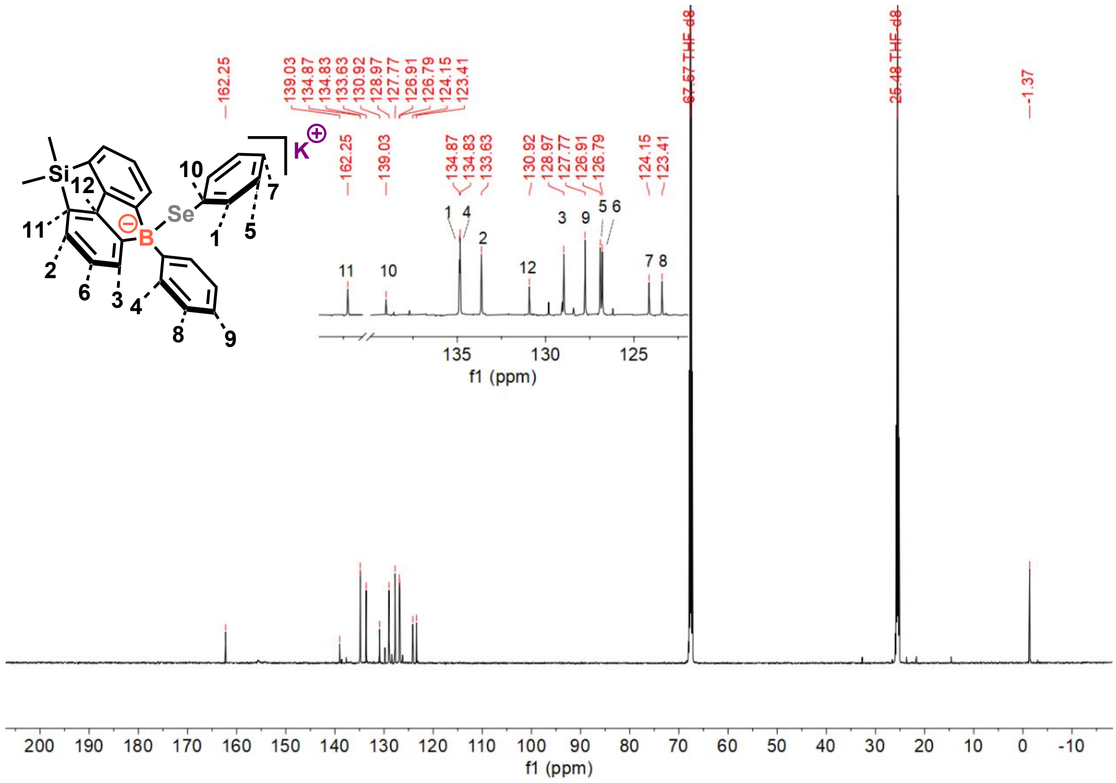

Figure S33. $^{13}C\{^{1}H\}$ NMR (150.93 MHz) spectrum of **8** in THF-d_8.

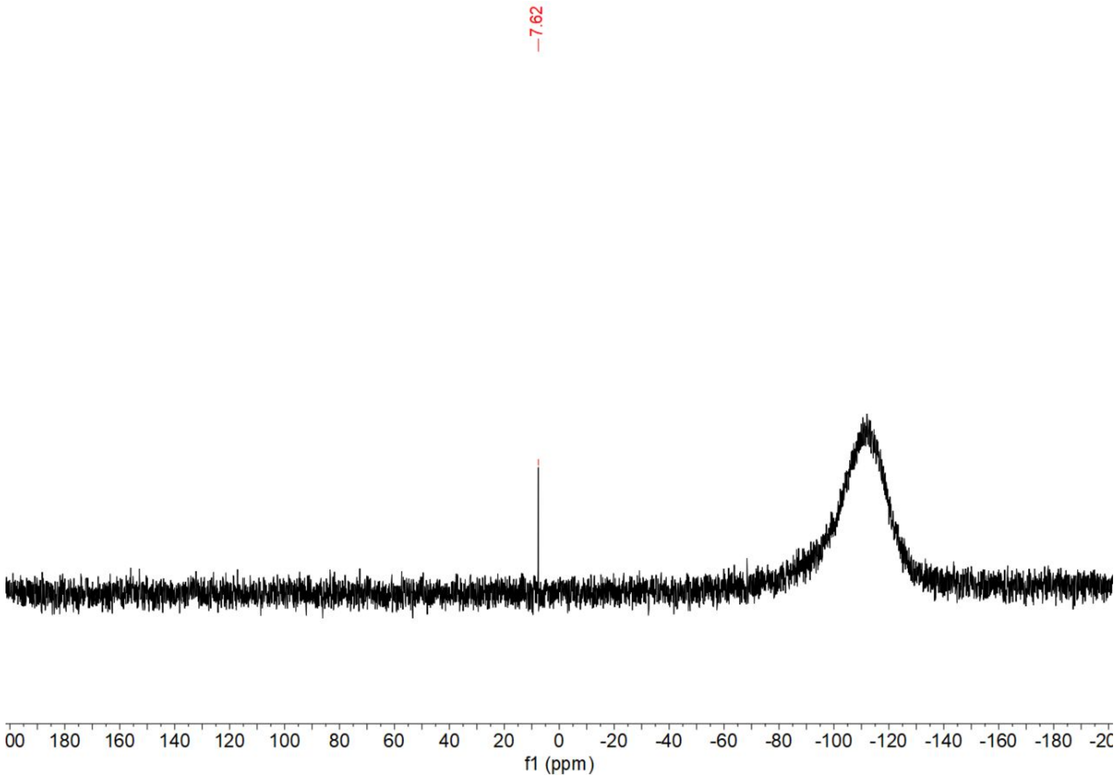

Figure S34. ^{29}Si NMR (119.24 MHz) spectrum of **8** in THF-d_8.

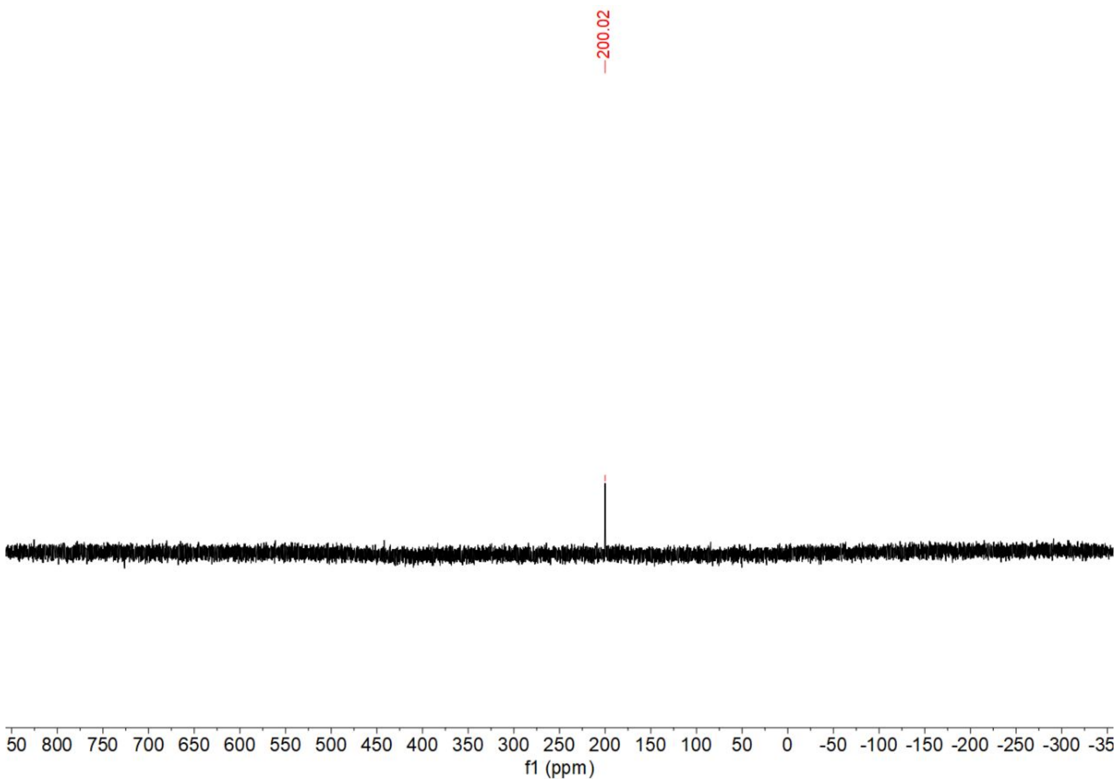

Figure S35. ^{77}Se NMR (114.49 MHz) spectrum of **8** in THF-d_8.

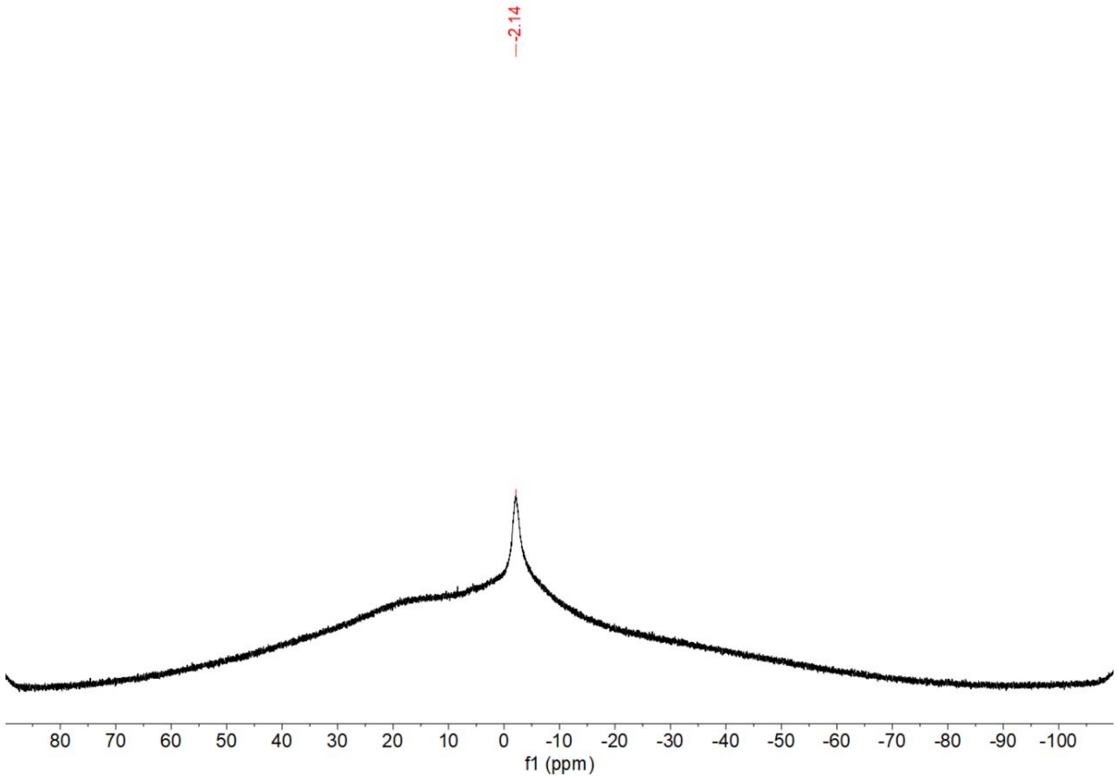

Figure S36. ^{11}B NMR (192.56 MHz) spectrum of **8** in THF-d_8.

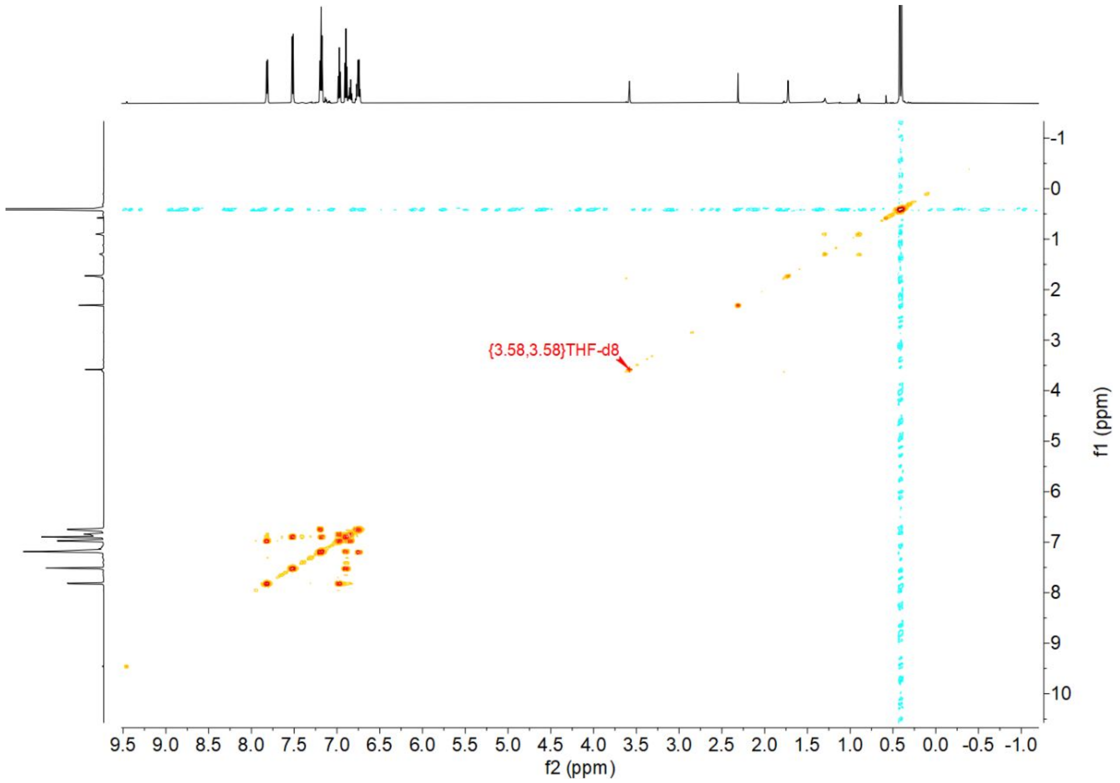

Figure S37. ^{1}H-^{1}H COSY NMR (600.17, 600.17 MHz) spectrum of **8** in THF-d_8.

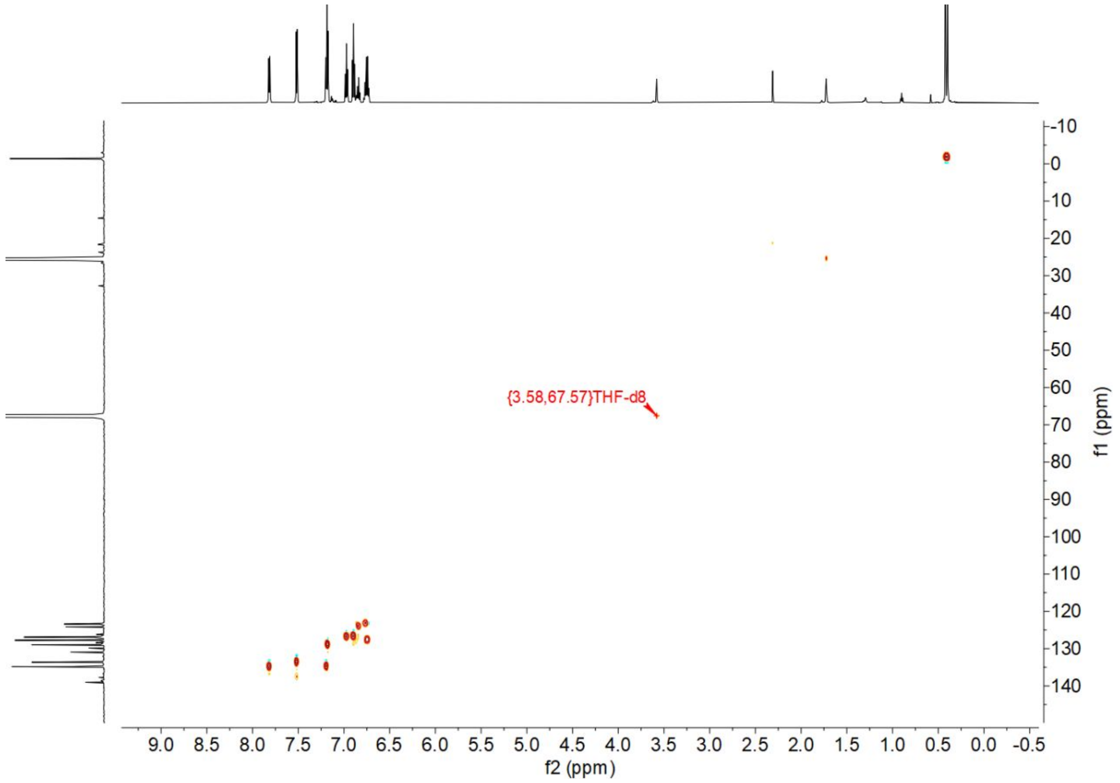

Figure S38. ^{1}H-^{13}C HSQC NMR (600.17, 150.91 MHz) spectrum of **8** in THF-d_8.

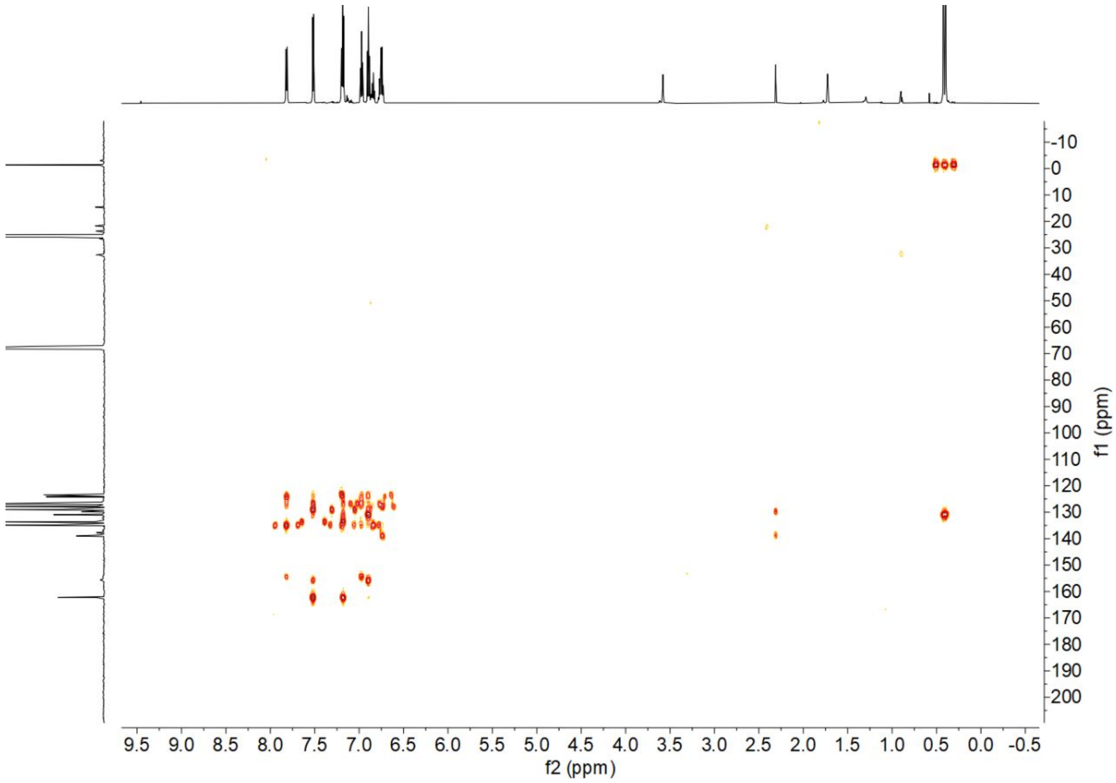

Figure S39. ^{1}H-^{13}C HMBC NMR (600.17, 150.91 MHz) spectrum of **8** in THF-d_8.

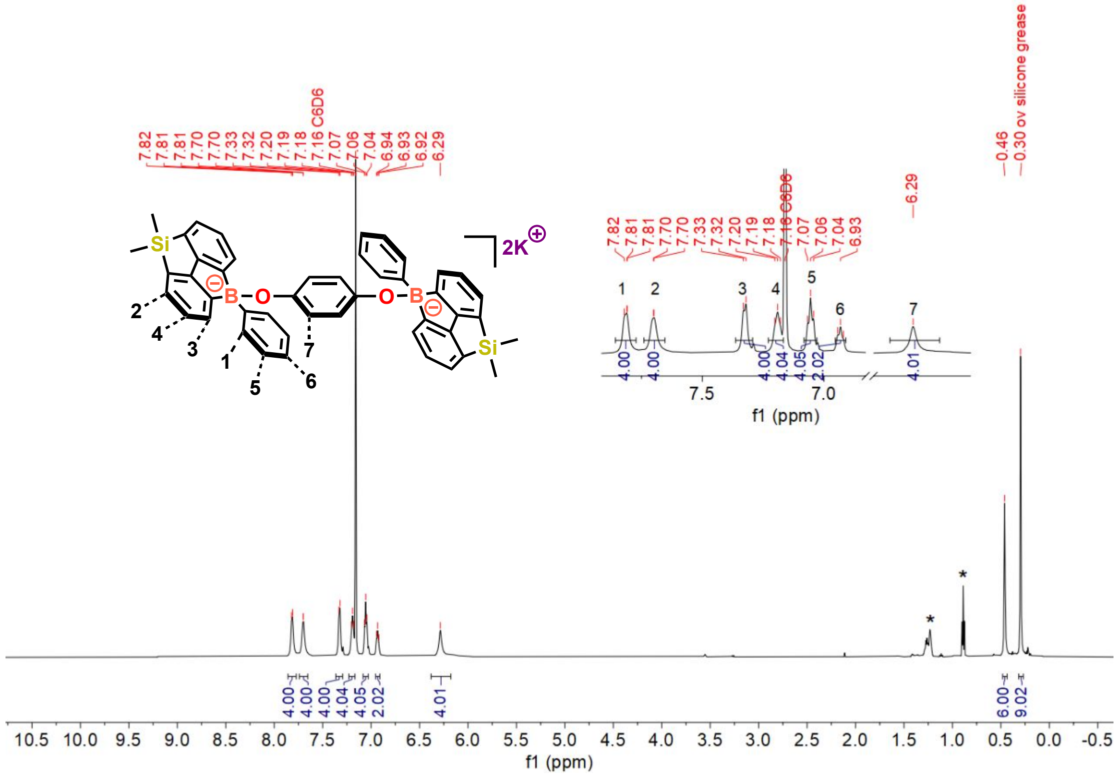

Figure S40. ^{1}H NMR (600.17 MHz) spectrum of compound **9** in C_6D_6.

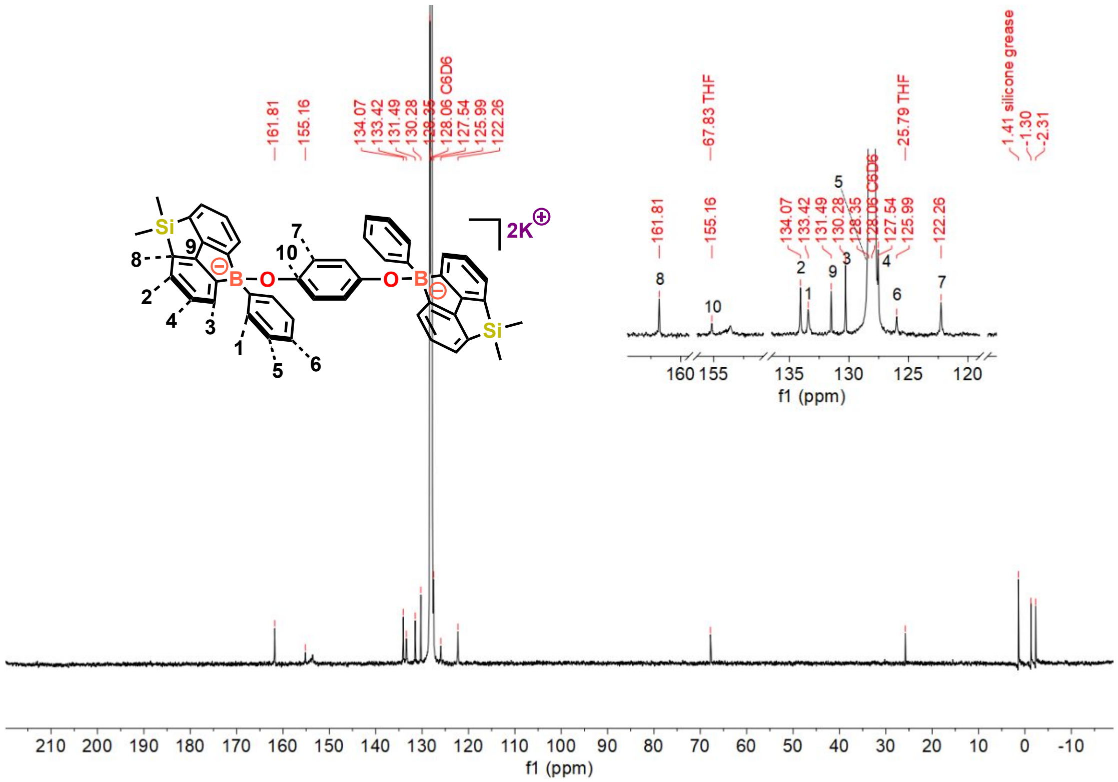

Figure S41. $^{13}C\{^{1}H\}$ NMR (150.93 MHz) spectrum of **9** in C_6D_6.

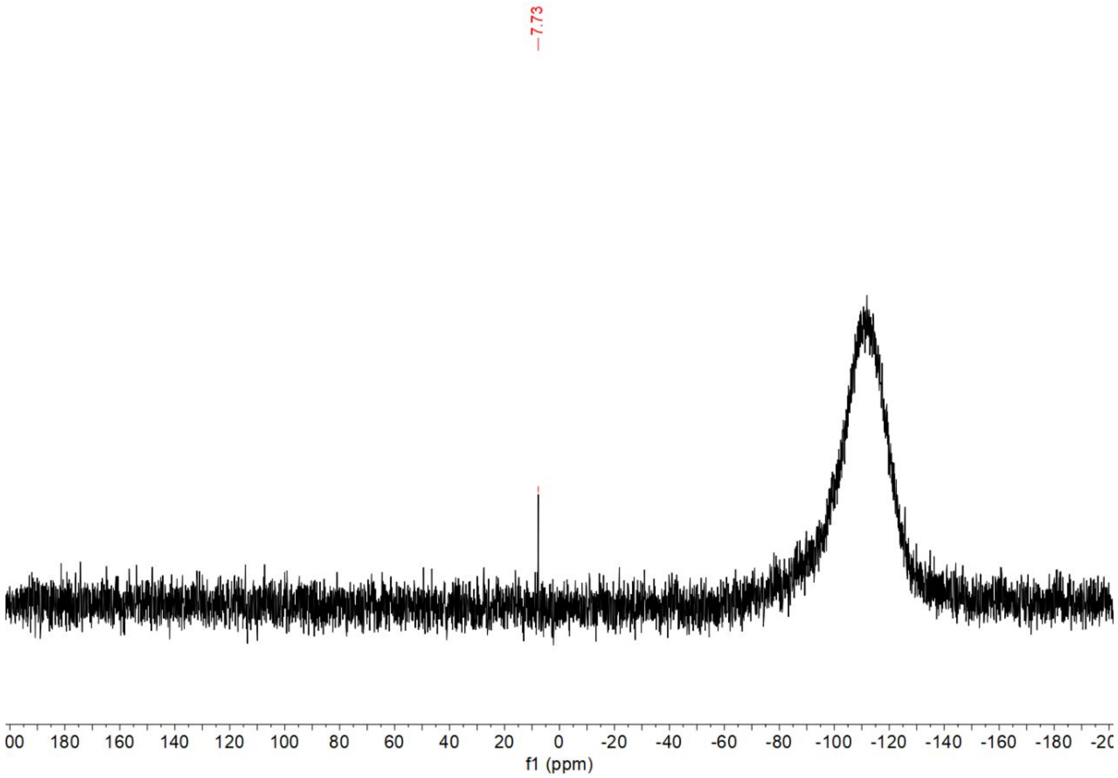

Figure S42. ^{29}Si NMR (119.24 MHz) spectrum of **9** in C_6D_6.

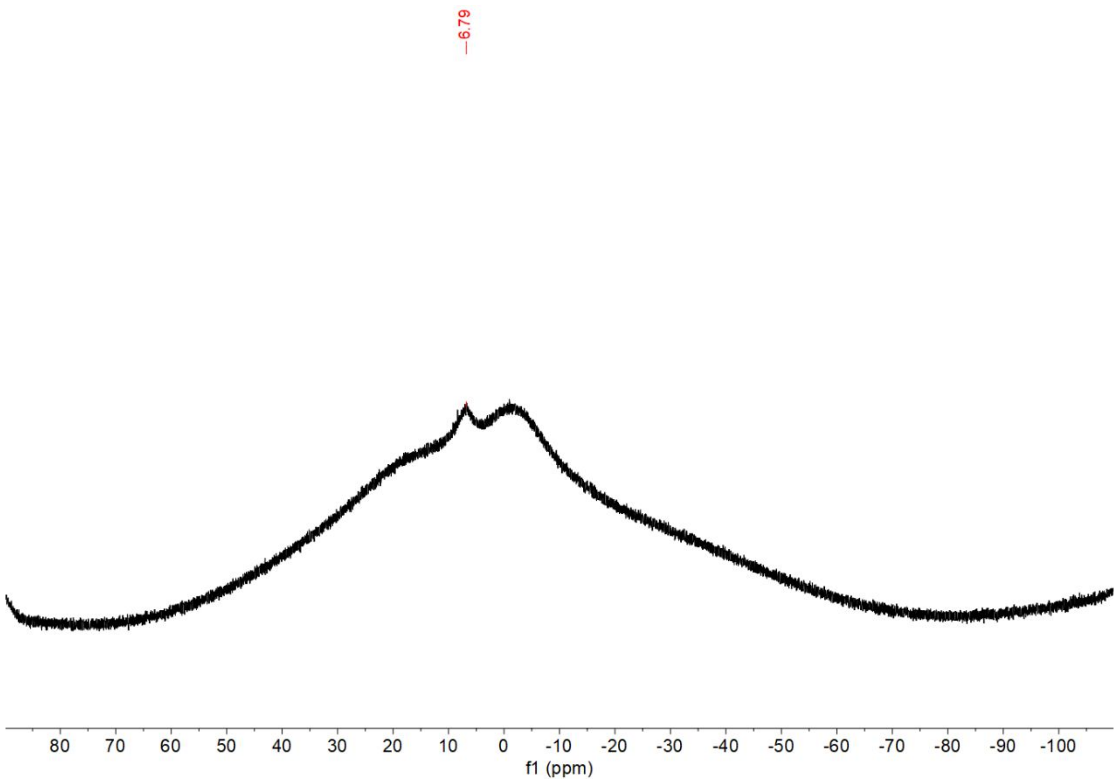

Figure S43. ^{11}B NMR (192.56 MHz) spectrum of **9** in C_6D_6.

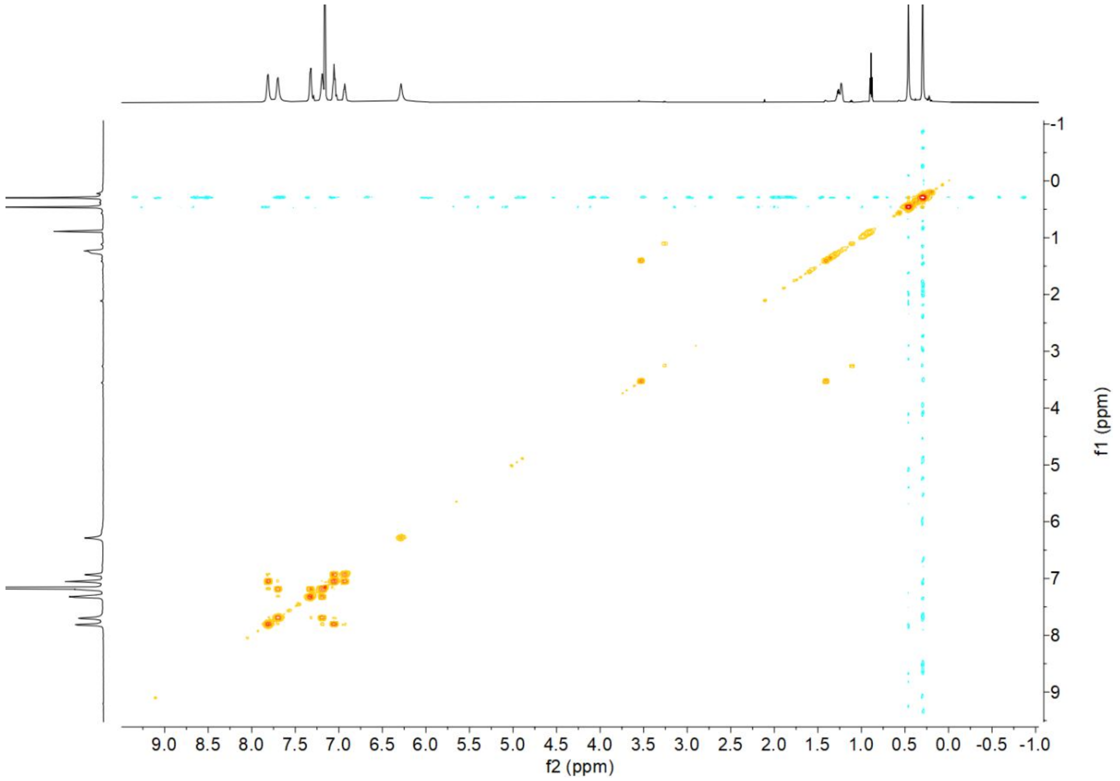

Figure S44. ^{1}H-^{1}H COSY NMR (600.17, 600.17 MHz) spectrum of **9** in C_6D_6.

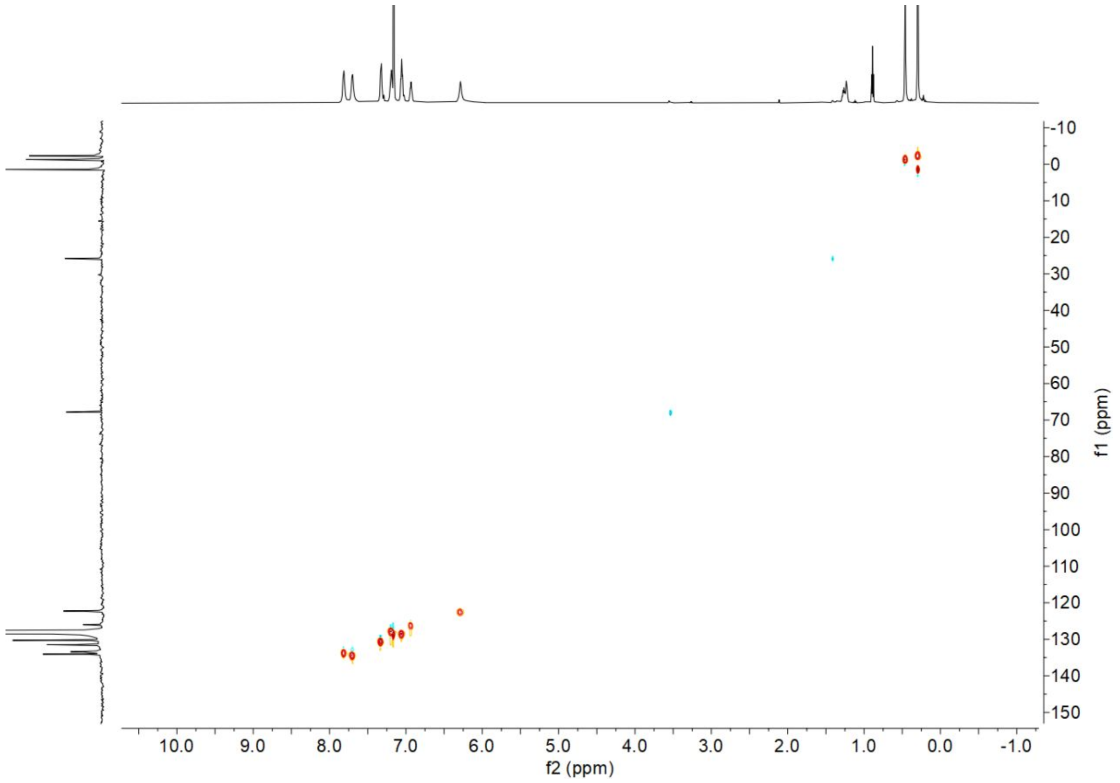

Figure S45. ^{1}H-^{13}C HSQC NMR (600.17, 150.91 MHz) spectrum of **9** in C_6D_6.

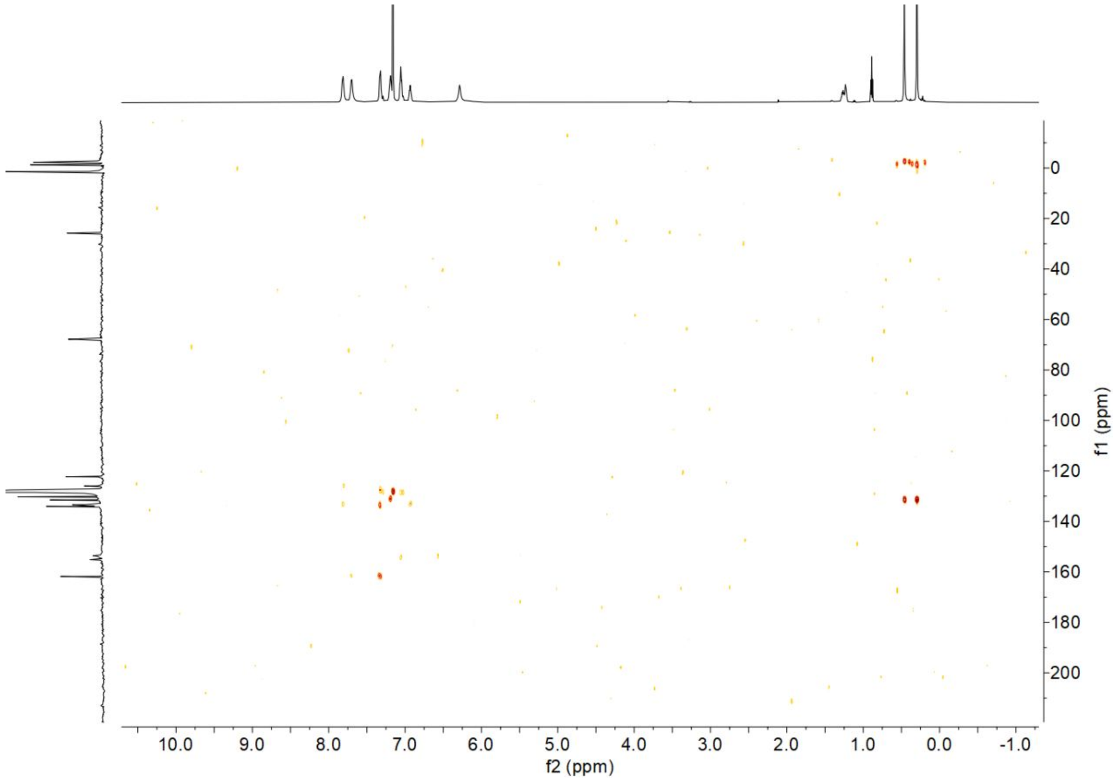

Figure S46. ^{1}H-^{13}C HMBC NMR (600.17, 150.91 MHz) spectrum of **9** in C_6D_6.

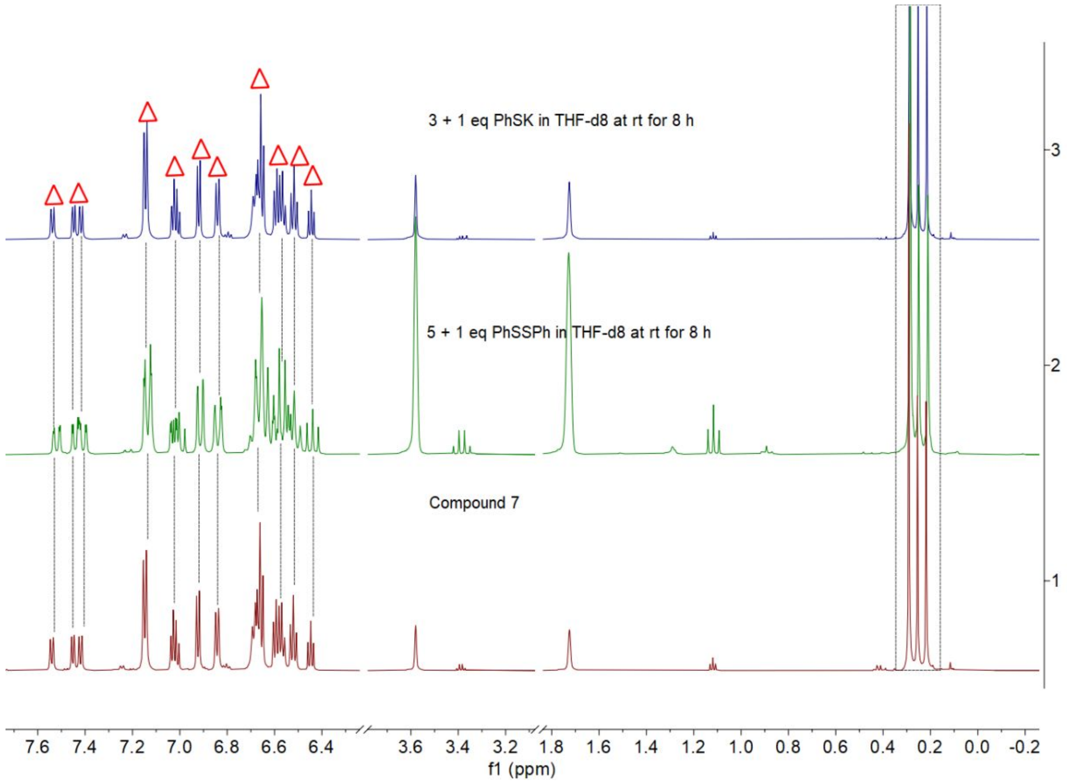

Figure S47. ^{1}H NMR Spectra of Control Experiments. Top: ^{1}H NMR spectrum of the reaction between compound **3** and PhSK in THF-d_8 at room temperature after 8 hours. Middle: ^{1}H NMR spectrum of the reaction between compound **5** and PhSSPh in THF-d_8 at room temperature after 8 hours. Bottom: ^{1}H NMR spectrum of compound **7** in THF-d_8.

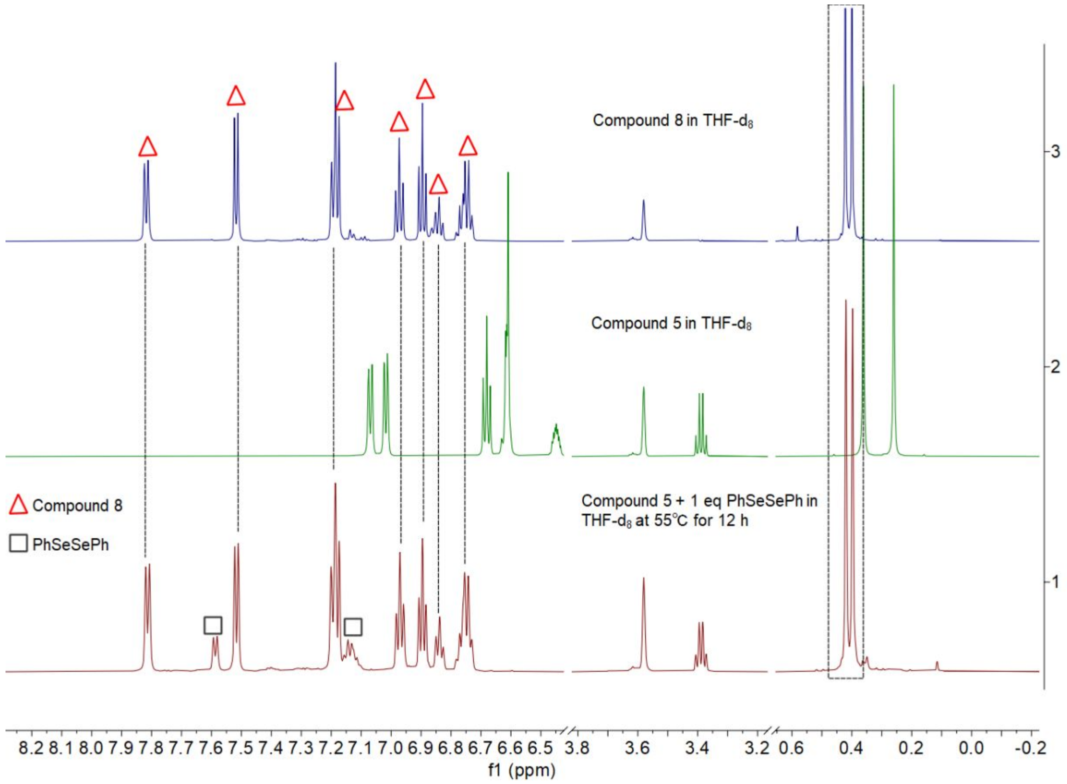

Figure S48. ¹H NMR Spectra of Control Experiments. Top: ¹H NMR spectrum of compound **8** in THF-d_8. Middle: ¹H NMR spectrum of compound **5** in THF-d_8. Bottom: ¹H NMR spectrum of the reaction between compound **5** and PhSeSePh in THF-d_8 at 55 ^{o}C after 12 hours.

3. X-Ray Crystallography Details

3.1. X-ray Crystallography Collection and Refinement Details

All single crystals were mounted on a Hampton loop using polybutene. The X-ray intensity data were measured on a Bruker D8 Venture diffractometer with a PHOTON II CMOS detector at low temperature using Cu-Kα radiation (λ = 1.54178 Å) or Mo-Kα radiation (λ = 0.71073Å). Data was collected using multi-scan (φ and ω scans) and was integrated by SANIT and scaled with either a numerical or multi-scan absorption correction using SADABS. [5] Structures were solved by SHELXT[6] and refined with SHELXL[7] using the OLEX2 program. [8] All non-hydrogen atoms were refined anisotropically, and all hydrogen atoms were added at idealized positions and refined using the riding model. Crystallographic data have been deposited with the Cambridge Crystallographic Data Center as supplementary publication (CCDC Deposition Number 2475521-2475528). For 2475524, the highly disordered THF was modeled in two parts, using the SIMU and SADI restraints. The B alert arises from the highly disordered THF molecules around the central potassium (K). However, this disorder does not affect the main structure. The alert is associated with the Ueq of the main molecule compared to those of potassium's neighboring atoms. These data can be obtained free of charge from The Cambridge Crystallographic Data Centre via http://www.ccdc.cam.ac.uk/data_request/cif.

3.2.X-ray Data

Identification code (CCDC)	**2** (2475522)	**3** (2475523)	**4** (2475524)	**5** (2475525)
Empirical formula	$C_{16}H_{18}SiSn$	$C_{43.5}H_{38}B_2Si_2$	$C_{64}H_{81.03}B_2KO_6Si_2$	$C_{69.2}H_{89.8}B_2K_2N_{2.6}O_{12}Si_2$
Formula weight	357.08	638.54	1063.21	1306.03
Temperature/K	193.00	193.00	233	232.99
Crystal system	orthorhombic	monoclinic	monoclinic	triclinic
Space group	$Pna2_1$	$P2_1/n$	$P2_1/c$	P-1
a/Å	17.0101(11)	12.7866(9)	11.3212(18)	12.9427(16)
b/Å	8.3207(5)	11.6914(8)	17.259(4)	14.4064(19)
c/Å	10.7683(7)	25.028(2)	31.919(5)	20.330(2)
α/°	90	90	90	85.338(4)
β/°	90	104.226(4)	91.303(5)	77.262(4)
γ/°	90	90	90	77.130(4)
Volume/Å^3	1524.10(17)	3626.7(5)	6235(2)	3602.5(8)
Z	4	4	4	2
ρ_{calc}g/cm^3	1.556	1.169	1.133	1.204
μ/mm^{-1}	1.736	1.098	0.171	0.224
F(000)	712.0	1348.0	2280.0	1390.0
Crystal size/mm^3	0.22 × 0.15 × 0.14	0.24 × 0.21 × 0.18	0.22 × 0.14 × 0.07	0.15 × 0.14 × 0.04
Radiation	MoKα (λ = 0.71073)	CuKα (λ = 1.54178)	MoKα (λ = 0.71073)	MoKα (λ = 0.71073)
2θ range for data collection/°	4.79 to 56.68	7.168 to 136.53	4.466 to 52.126	4.706 to 55.094
Index ranges	-22 ≤ h ≤ 22, -11 ≤ k ≤ 11, -14 ≤ l ≤ 14	-15 ≤ h ≤ 15, -14 ≤ k ≤ 14, -30 ≤ l ≤ 30	-13 ≤ h ≤ 13, -21 ≤ k ≤ 17, -39 ≤ l ≤ 38	-16 ≤ h ≤ 16, -18 ≤ k ≤ 18, -26 ≤ l ≤ 26
Reflections collected	26816	40525	46167	52434
Independent reflections	3686 [R_{int} = 0.0427, R_{sigma} = 0.0283]	6634 [R_{int} = 0.1057, R_{sigma} = 0.0682]	12101 [R_{int} = 0.0969, R_{sigma} = 0.0887]	16493 [R_{int} = 0.0940, R_{sigma} = 0.1064]
Data/restraints/parameters	3686/1/169	6634/0/401	12101/377/817	16493/575/906
Goodness-of-fit on F^2	1.141	1.062	1.037	1.023
Final R indexes [I>=2σ (I)]	R_1 = 0.0268, wR_2 = 0.0578	R_1 = 0.0618, wR_2 = 0.1661	R_1 = 0.0810, wR_2 = 0.1721	R_1 = 0.0647, wR_2 = 0.1524
Final R indexes [all data]	R_1 = 0.0369, wR_2 = 0.0655	R_1 = 0.0855, wR_2 = 0.1812	R_1 = 0.1655, wR_2 = 0.2198	R_1 = 0.1187, wR_2 = 0.1791
Largest diff. peak/hole / e Å^{-3}	0.74/-0.55	0.37/-0.40	0.23/-0.38	0.43/-0.30
Flack parameter	0.015(18)	-	-	-

Identification code (CCDC)	**6** (2475521)	**7** (2475527)	**8** (2475526)	**9** (2475528)
Empirical formula	$C_{54}H_{59}B_2KO_3Si_2$	$C_{98}H_{93}B_4K_2O_{1.5}S_2Si_4$	$C_{38}H_{46}BKO_3SeSi$	$C_{88}H_{122}B_2K_2N_4O_{15.5}Si_2$
Formula weight	872.91	1592.64	707.71	1639.89
Temperature/K	223.01	222.99	213.0	213.00
Crystal system	monoclinic	monoclinic	monoclinic	triclinic
Space group	C2/c	$P2_1/n$	$P2_1/c$	P-1
a/Å	14.5212(16)	23.8067(6)	20.7077(5)	10.1557(3)
b/Å	21.219(2)	18.7874(4)	9.2865(2)	12.5846(4)
c/Å	17.8248(18)	23.8242(6)	19.5417(4)	18.5991(6)
α/°	90	90	90	93.812(2)
β/°	110.358(4)	119.474(2)	99.599(2)	92.955(2)
γ/°	90	90	90	102.9160(10)
Volume/Å^3	5149.2(10)	9276.7(4)	3705.29(14)	2306.40(13)
Z	4	4	4	1
ρ_{calc}g/cm^3	1.126	1.140	1.269	1.181
μ/mm^{-1}	0.189	2.161	2.944	1.657
F(000)	1856.0	3356.0	1480.0	878.0
Crystal size/mm^3	0.19 × 0.08 × 0.06	0.21 × 0.12 × 0.06	0.29 × 0.16 × 0.09	0.18 × 0.05 × 0.04
Radiation	MoKα (λ = 0.71073)	CuKα (λ = 1.54178)	CuKα (λ = 1.54178)	CuKα (λ = 1.54178)
2θ range for data collection/°	4.548 to 55.146	6.348 to 137.046	9.18 to 141.844	7.23 to 137.566
Index ranges	-18 ≤ h ≤ 18, -22 ≤ k ≤ 27, -23 ≤ l ≤ 23	-25 ≤ h ≤ 28, -22 ≤ k ≤ 22, -28 ≤ l ≤ 26	-25 ≤ h ≤ 25, -11 ≤ k ≤ 10, -23 ≤ l ≤ 23	-12 ≤ h ≤ 12, -14 ≤ k ≤ 15, -22 ≤ l ≤ 22
Reflections collected	25279	61969	31672	23794
Independent reflections	5935 [R_{int} = 0.0919, R_{sigma} = 0.0792]	16985 [R_{int} = 0.0869, R_{sigma} = 0.0760]	7031 [R_{int} = 0.0792, R_{sigma} = 0.0564]	8465 [R_{int} = 0.0896, R_{sigma} = 0.0868]
Data/restraints/parameters	5935/30/306	16985/0/991	7031/120/445	8465/0/480
Goodness-of-fit on F^2	1.013	1.030	1.036	1.055
Final R indexes [I>=2σ (I)]	R_1 = 0.0547, wR_2 = 0.1300	R_1 = 0.0701, wR_2 = 0.1815	R_1 = 0.0441, wR_2 = 0.1073	R_1 = 0.0535, wR_2 = 0.1444
Final R indexes [all data]	R_1 = 0.1071, wR_2 = 0.1559	R_1 = 0.0925, wR_2 = 0.1988	R_1 = 0.0616, wR_2 = 0.1193	R_1 = 0.0752, wR_2 = 0.1596
Largest diff. peak/hole / e Å^{-3}	0.24/-0.36	0.42/-0.39	0.36/-0.46	0.28/-0.24
Flack parameter	-	-	-	-

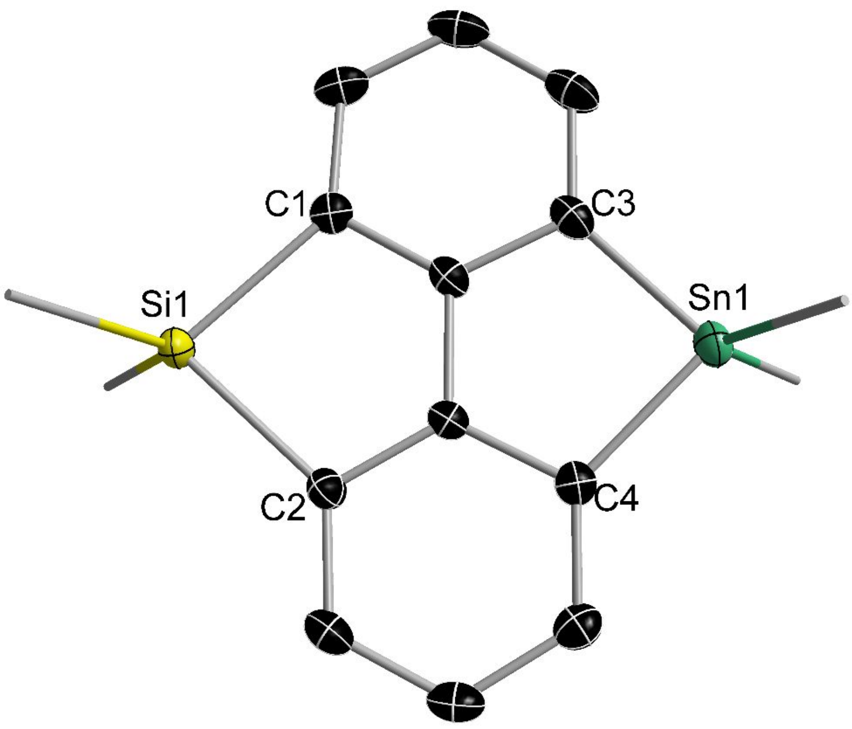

Figure S49. Molecular structure of **2**. Thermal ellipsoids are shown at 30% probability and H atoms were omitted for clarity. Selected bond lengths (in Å): Si1–C1 1.937(5), Si1–C2 1.971(5), Sn1–C3 2.107(5), Sn1–C4 2.116(4).

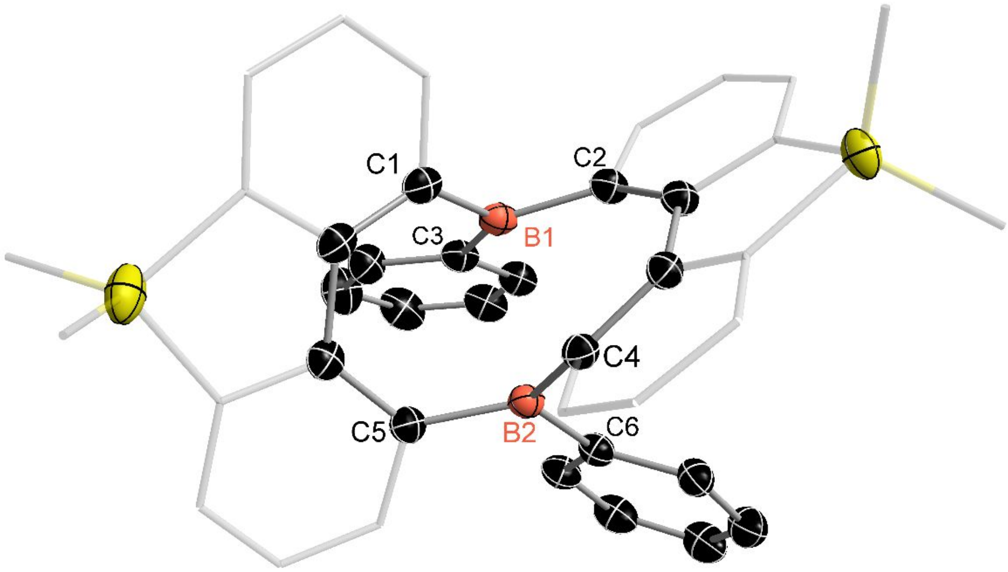

Figure S50. Molecular structure of **3**. Thermal ellipsoids are shown at 30% probability and H atoms were omitted for clarity. Selected bond lengths (in Å): B1–C1 1.571(4), B1–C2 1.569(4), B1–C3 1.578(3), B2–C4 1.578(3), B2–C5 1.566(4), B2–C6 1.575(4).

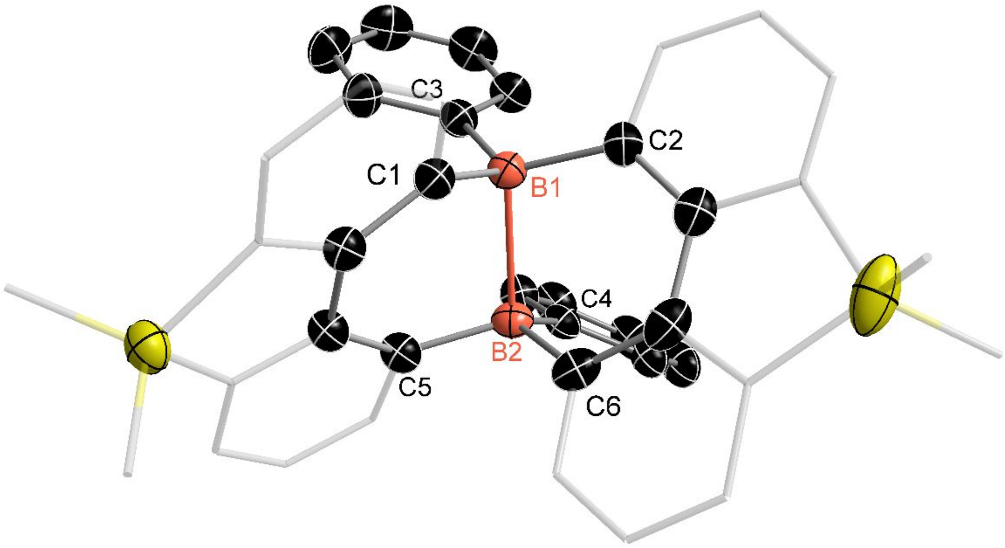

Figure S51. Molecular structure of **4**. Thermal ellipsoids are shown at 30% probability and

H atoms and counter ion $[K(THF)_6]^+$ were omitted for clarity. Selected bond lengths (in Å): B1–C1 1.606(5), B1–C2 1.597(5), B1–C3 1.584(5), B2–C4 1.587(5), B2–C5 1.592(5), B2–C6 1.595(6), B1–B2 2.114(5).

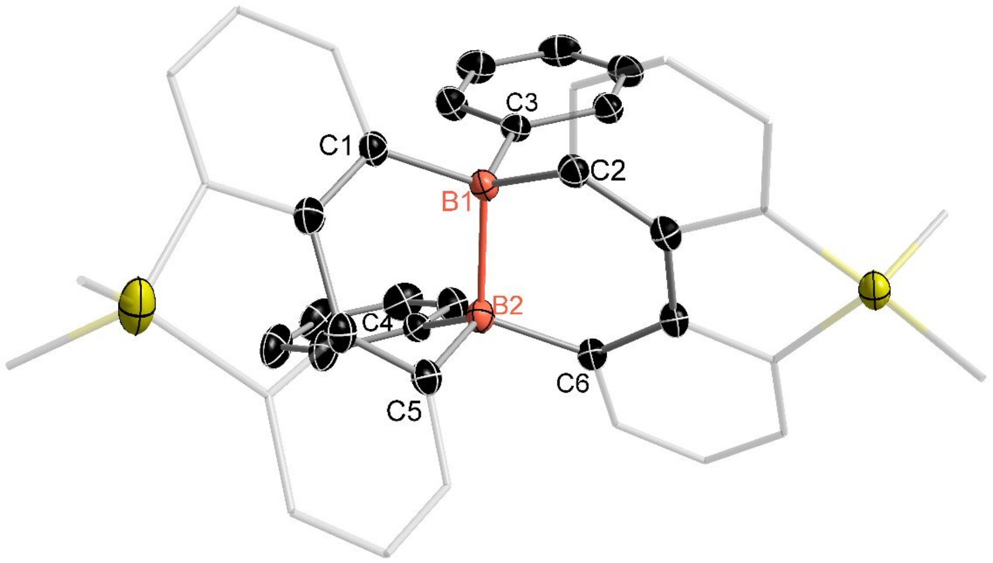

Figure S52. Molecular structure of **5**. Thermal ellipsoids are shown at 30% probability and H atoms and counter ion $[K(18\text{-crown-}6)]_2^{2+}$ were omitted for clarity. Selected bond lengths (in Å): B1–C1 1.626(4), B1–C2 1.638(3), B1–C3 1.620(3), B1–C4 1.612(4), B1–C5 1.627(4), B1–C6 1.640(3), B1–B2 1.821(4).

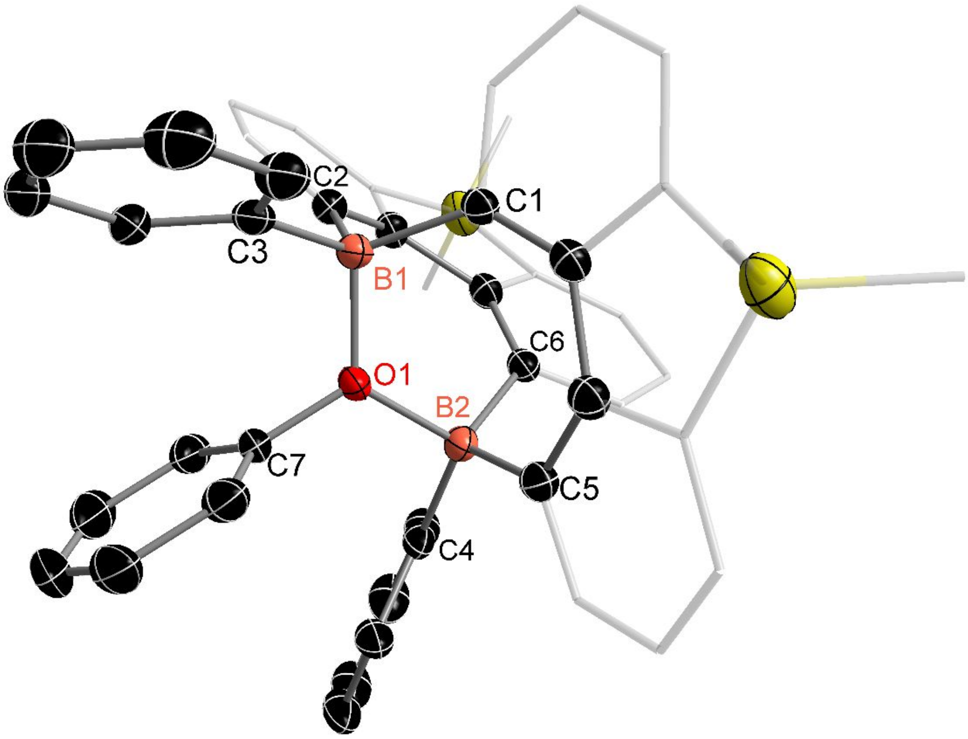

Figure S53. Molecular structure of **6**. Thermal ellipsoids are shown at 30% probability and H atoms and counter ion $[K(Et_2O)_2]^+$ were omitted for clarity. Selected bond lengths (in Å): B1–C1 1.629(3), B1–C2 1.617(3), B1–C3 1.649(3), B2–C4 1.649(3), B2–C5 1.617(3), B2–C6 1.629(3), B1–O1 1.583(2), B2–O1 1.583(2), C7–O1 1.395(3).

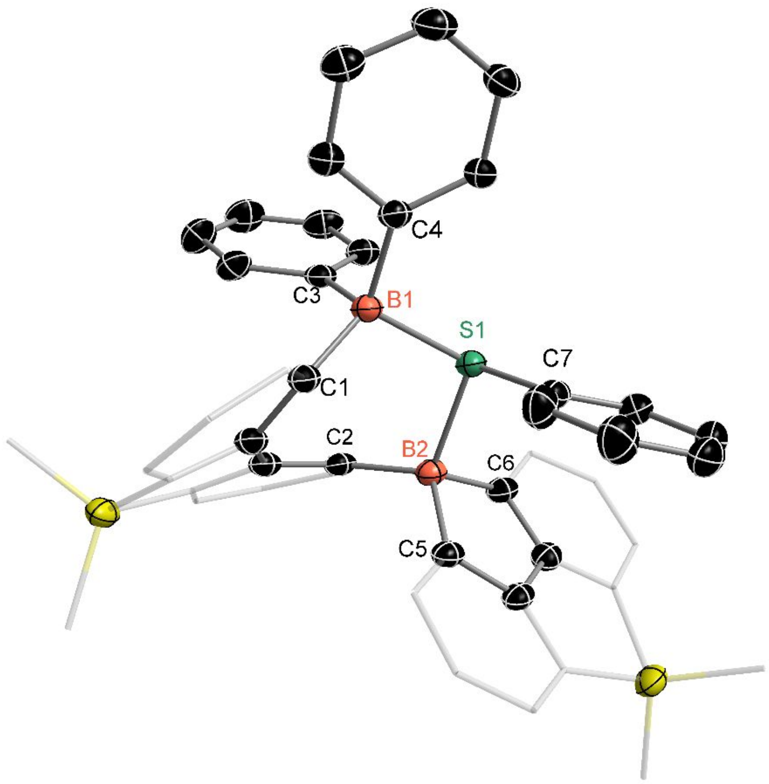

Figure S54. Molecular structure of **7**. Thermal ellipsoids are shown at 30% probability and H atoms, counter ion K^+, and free Et_2O were omitted for clarity. Selected bond lengths (in Å): B1–C1 1.627(5), B2–C2 1.611(5), B1–C3 1.627(6), B1–C4 1.641(5), B2–C5 1.655(5), B2–C6 1.664(5), B2–S1 1.977(4), S1–C7 1.778(4), B1–S1 1.975(4).

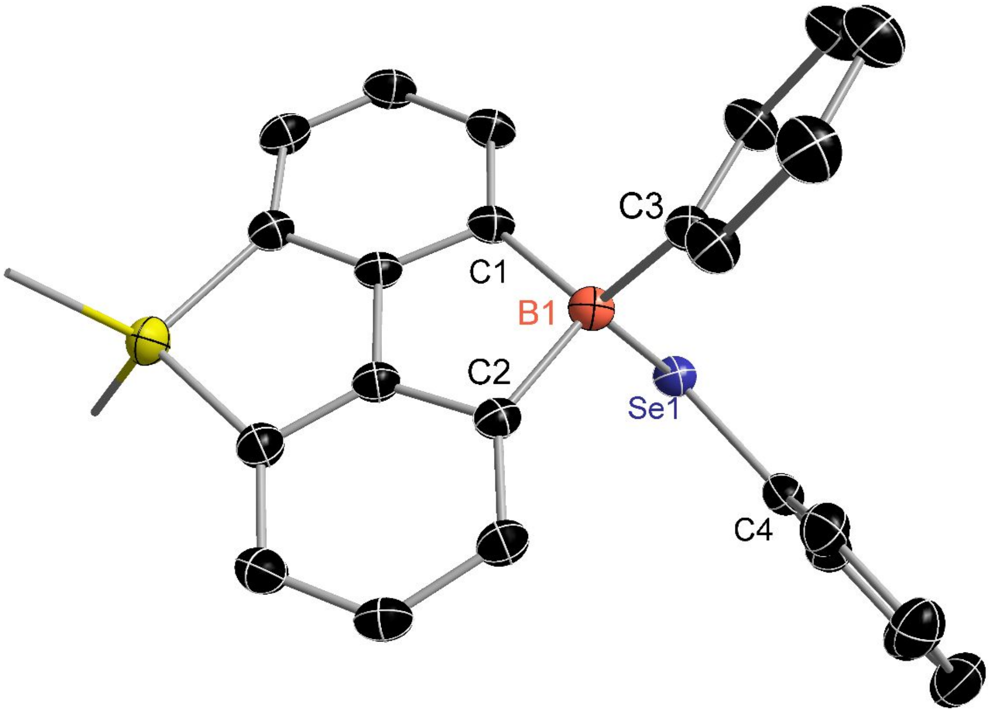

Figure S55. Molecular structure of **8**. Thermal ellipsoids are shown at 30% probability and H atoms and counter ion $[K(THF)_3]^+$ were omitted for clarity. Selected bond lengths (in Å): B1–C1 1.652(4), B1–C2 1.662(4), B1–C3 1.597(4), B1–Se1 2.126(3), Se1–C4 1.916(3).

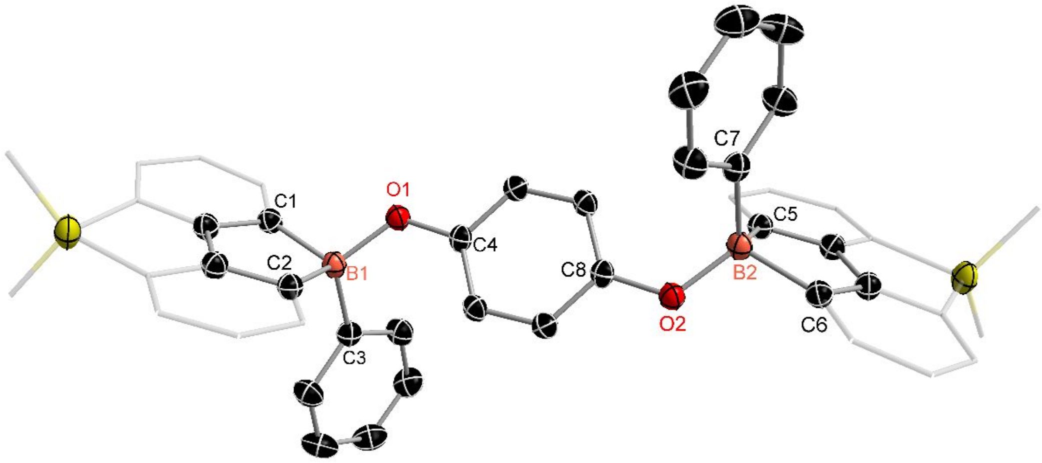

Figure S56. Molecular structure of **9**. Thermal ellipsoids are shown at 30% probability and

H atoms and counter ion $[K(2,2,2\text{-cryptand})]_2^{2+}$ were omitted for clarity. Selected bond lengths (in Å): B1–C1 1.653(3), B1–C2 1.686(3), B1–C3 1.642(3), B1–O1 1.490(3), O1–C4 1.354(2), B2–C5 1.686(3), B2–C6 1.653(3), B2–C7 1.642(3), B2–O2 1.490(3), O2–C8 1.354(2).

4. Cyclic Voltammetry Spectrum of 3

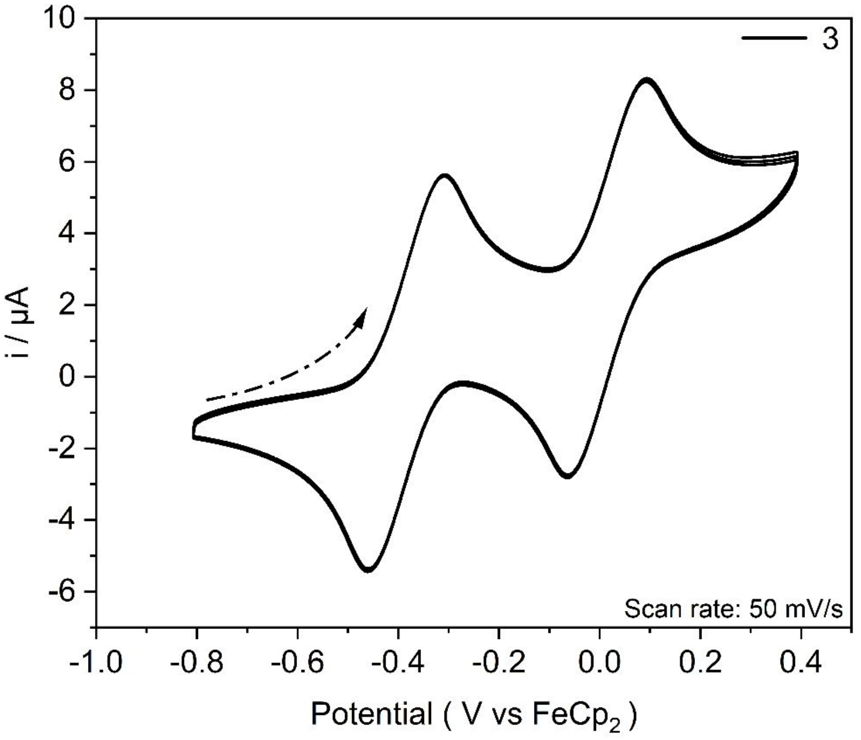

Figure S57. Cyclic Voltammograms of neutral **3** in anhydrous THF containing 0.1 M *n*Bu_4NPF_6, at sweep rate = 50 mV/s. (E_{redox} = -0.384 V, 0.014 V)

5. Computational Section

All intermediates (INTs) and transition states (TSs) were initially optimized at the (U)B3LYP-D3(BJ)/ma-SVP level.[9-11] Solvent effects of tetrahydrofuran (THF) were incorporated using the Polarizable Continuum Model (PCM).[12, 13] To obtain accurate reaction energies, single point (SP) energy calculations were subsequently performed at the (U)B3LYP-D3(BJ)/ma-TZVP (THF, PCM) level on all the optimized structures.[11] The final Gibbs free energies at 298 K (ΔG_{298}) reported in the manuscript were obtained by combining the SP energies at the ma-TZVP level with the Gibbs free energy and temperature corrections obtained at the ma-SVP level. This composite approach is denoted as (U)B3LYP-D3(BJ)/ma-TZVP//(U)B3LYP-D3(BJ)/ma-SVP (THF, PCM). The nature of all stationary points was confirmed through vibrational frequencies calculations: Minimum (INTs) showed no imaginary frequency, while transition states (TSs) exhibited exactly one imaginary frequency along the reaction coordinate. All calculations were performed using Gaussian 16 quantum chemistry software package.[16] The natural bond orbital (NBO) analysis was performed with the NBO 7.0 program.[17,18] The image of molecular orbitals (MOs) was generated by IboView program (v20211019).[19,20] The atoms in molecules (AIM) and non-covalent interaction (NCI) analyses were performed using Multiwfn (version 3.8),[21,22] with wavefunctions calculated at the ma-TZVP level. Graphic of NCI(0.3) analysis was prepared using VMD (version 1.9.3).[23]

Table S2. Comparison of the structural parameters of crystal structures and the DFT optimized structures at the (U)B3LYP-D3(BJ)/ma-SVP level in PCM(THF).

B-B bond length (Å)	Crystal	DFT [a]
Compound **3**	2.660	2.661
Compound **4**	2.114	2.147
Compound **5**	1.821	1.828
Sums of the C-B-C angles (°)		
Compound **3**	357.62	358.5
	358.30	
Compound **4**	346.20	348.5
	343.86	
Compound **5**	333.30	333.7
	331.23	

Table S3. The Mayer-Mulliken bond order and the hybridization of the B-B bond obtained at the (U)B3LYP-D3(BJ)/ma-SVP level.

	Optimized bond length (Å)	Mayer-Mulliken bond order	Bonding hybridization σ(B-B)
Compound **4**	2.147	0.60	0.71($sp^{5.9}$)B+0.71($sp^{5.9}$)B
Compound **5**	1.828	1.22	0.71($sp^{3.0}$)B+0.71($sp^{3.0}$)B
H_2BBH_2	1.643	1.29	0.71($sp^{1.6}$)B+0.71($sp^{1.6}$)B

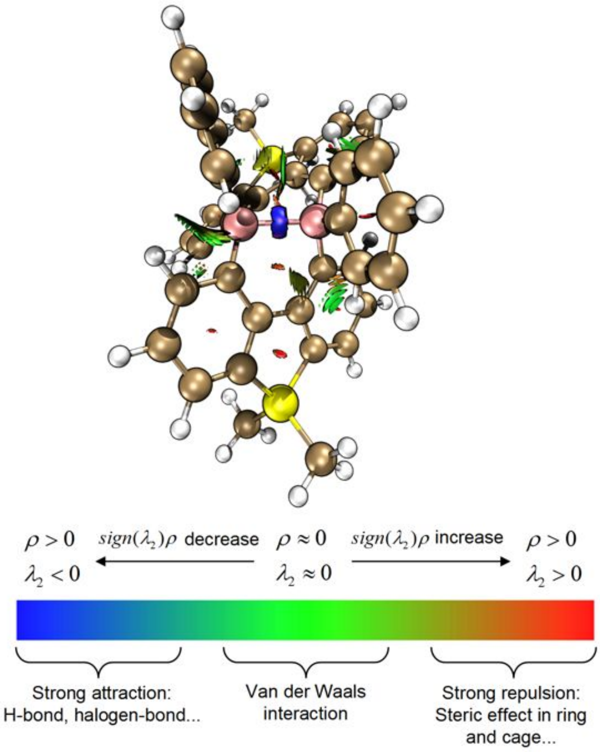

Figure S58. NCI(0.30) isosurface plot of compound **4**. The standard coloring method and chemical explanation of ρ and sign(λ_2) can be found on Ref. 22 in SI.

Table S4. Cartesian coordinates for the optimized geometries at the (U)B3LYP-D3(BJ)/ma-SVP (THF, PCM) level. (Sequence: compound **3**, **4**, **5**, **7**, PhSSPh, PhSSPh⁻, PhS•, PhS⁻, **TS-1**, **TS-2**, **INT-2**, **TS-3**, **INT-3**, **TS-4**, **INT-4**.)

compound **3** (neutral, singlet)

C	-0.80684800	1.96330600	1.60995600
C	-1.56844800	0.61703500	-1.39899400
C	0.80685000	1.96329600	-1.60996200
C	0.75682800	-0.72519300	-1.30010600
C	-2.37554600	-0.27642900	-0.64601900
C	2.37554500	-0.27642900	0.64601900
C	1.56844800	0.61703900	1.39899100
C	1.91328600	-1.03535100	-0.54611700
C	-0.75683100	-0.72518400	1.30010900
C	-2.19621000	1.31200100	-2.45963000
H	-1.60466500	2.00032900	-3.06571700
C	3.71107700	-0.55772900	1.04081300
C	2.74388300	-2.13002800	-0.90998400
C	-2.16989300	1.91729800	1.97902900
H	-2.66861300	0.95051200	2.06339000
C	0.20797900	3.23845000	-1.52460400
H	-0.83582700	3.31815400	-1.22010500
C	2.16989500	1.91728600	-1.97903500
H	2.66861400	0.95049900	-2.06339400
C	-1.91328900	-1.03534500	0.54612100
C	2.19621200	1.31200900	2.45962300
H	1.60466800	2.00033900	3.06570800
C	0.36382600	-1.65220300	-2.29098000
H	-0.54875800	-1.46180800	-2.86060200
C	-0.20797400	3.23845800	1.52459600
H	0.83583100	3.31816000	1.22009600
C	2.34802100	-2.99092000	-1.93588900
H	2.98111800	-3.83503200	-2.22437400
C	3.53107100	1.09516600	2.80603000
H	3.97375300	1.64164400	3.64236300
C	1.13331800	-2.77589600	-2.60118900
H	0.80477800	-3.46152800	-3.38618300
C	4.27971800	0.13602800	2.11111800
H	5.30684800	-0.07597700	2.42236900
C	-3.71107700	-0.55773000	-1.04081300
C	-3.53106900	1.09515800	-2.80603700
H	-3.97375000	1.64163400	-3.64237200
C	-0.92553600	4.40676500	1.78521900
H	-0.43562300	5.37961100	1.69829400

B	0.00455100	0.65109700	1.33062000
B	-0.00455100	0.65109000	-1.33062200
C	-4.27971700	0.13602300	-2.11112200
H	-5.30684700	-0.07598200	-2.42237300
C	0.92554200	4.40675500	-1.78522800
H	0.43563100	5.37960200	-1.69830400
C	-0.36383200	-1.65218800	2.29099100
H	0.54875100	-1.46179100	2.86061300
C	-2.27597200	4.32979100	2.14484300
H	-2.84305500	5.24184800	2.34820600
C	-2.74388700	-2.13001900	0.90999300
C	2.89776400	3.07865600	-2.24294000
H	3.95095300	3.01152400	-2.52617800
C	-2.89775900	3.07867000	2.24293400
H	-3.95094800	3.01153900	2.52617200
C	2.27597900	4.32977900	-2.14485100
H	2.84306300	5.24183500	-2.34821400
C	-1.13332500	-2.77587800	2.60120500
H	-0.80478800	-3.46150600	3.38620300
C	-2.34802700	-2.99090600	1.93590300
H	-2.98112500	-3.83501600	2.22439100
Si	4.35586900	-2.03282600	0.06037800
Si	-4.35587000	-2.03282400	-0.06037500
C	-5.82244700	-1.65510900	1.05675300
H	-6.72440600	-1.44397200	0.45914600
H	-6.04263600	-2.51250500	1.71377300
H	-5.61262100	-0.77899900	1.68965800
C	-4.71487100	-3.51368200	-1.16326900
H	-3.84902600	-3.73979900	-1.80445600
H	-4.93953000	-4.40579400	-0.55587600
H	-5.58484400	-3.31601300	-1.81093700
C	4.71487800	-3.51367900	1.16327700
H	4.93953600	-4.40579300	0.55588600
H	5.58485300	-3.31600600	1.81094000
H	3.84903600	-3.73979600	1.80446800
C	5.82244000	-1.65511300	-1.05675800
H	6.72440100	-1.44396900	-0.45915700
H	6.04262900	-2.51251300	-1.71377300
H	5.61260900	-0.77900800	-1.68966800

compound **4** (anion, doublet)

C	-0.70971600	2.03841800	1.47112100
C	-1.59133800	0.55185800	-1.36518900
C	0.70971500	2.03841900	-1.47112000

C	0.78976300	-0.66085100	-1.32297400
C	-2.40398700	-0.34673000	-0.63336100
C	2.40398800	-0.34672800	0.63336000
C	1.59133800	0.55185900	1.36518800
C	1.94652800	-1.03638500	-0.60295000
C	-0.78976100	-0.66085200	1.32297300
C	-2.18428100	1.15189500	-2.49640500
H	-1.59076500	1.84515200	-3.09686400
C	3.70804300	-0.70138700	1.07092200
C	2.78735900	-2.09467700	-1.04479200
C	-2.08684900	2.07416800	1.79154600
H	-2.65947700	1.14505500	1.77760100
C	0.03159800	3.27881000	-1.51110100
H	-1.02598300	3.31231400	-1.24380500
C	2.08684700	2.07417100	-1.79154500
H	2.65947700	1.14505800	-1.77760200
C	-1.94652700	-1.03638600	0.60295000
C	2.18428200	1.15189800	2.49640300
H	1.59076600	1.84515500	3.09686100
C	0.44161900	-1.47454600	-2.42219800
H	-0.46122000	-1.23179100	-2.98866700
C	-0.03160100	3.27881000	1.51110200
H	1.02598000	3.31231500	1.24380700
C	2.41841300	-2.85289500	-2.15946500
H	3.05459600	-3.67134600	-2.51084700
C	3.48956400	0.85820900	2.90870200
H	3.90244700	1.34346400	3.79788400
C	1.22454600	-2.55915600	-2.83304800
H	0.91786000	-3.15758800	-3.69578700
C	4.24760300	-0.08799500	2.20614300
H	5.25080600	-0.35060600	2.55657000
C	-3.70804200	-0.70139000	-1.07092300
C	-3.48956300	0.85820500	-2.90870400
H	-3.90244600	1.34345800	-3.79788800
C	-0.67297600	4.47044500	1.85549700
H	-0.10853200	5.40738000	1.86820800
B	0.03200800	0.69421900	1.07323700
B	-0.03200700	0.69421900	-1.07323800
C	-4.24760100	-0.08799900	-2.20614400
H	-5.25080500	-0.35061100	-2.55657200
C	0.67297100	4.47044600	-1.85549500
H	0.10852600	5.40738100	-1.86820600
C	-0.44161700	-1.47454700	2.42219700
H	0.46122300	-1.23179100	2.98866500

C	-2.03890700	4.47116900	2.16630800
H	-2.54754000	5.40191600	2.43214500
C	-2.78735700	-2.09467800	1.04479200
C	2.74385100	3.26097200	-2.12886800
H	3.81118800	3.24252300	-2.36780900
C	-2.74385400	3.26096800	2.12886800
H	-3.81119100	3.24251700	2.36780800
C	2.03890200	4.47117200	-2.16630700
H	2.54753400	5.40192000	-2.43214400
C	-1.22454300	-2.55915600	2.83304700
H	-0.91785700	-3.15758900	3.69578600
C	-2.41841100	-2.85289600	2.15946400
H	-3.05459300	-3.67134700	2.51084700
Si	4.35082100	-2.11037400	0.00193600
Si	-4.35082000	-2.11037500	-0.00193500
C	-5.89347800	-1.69661200	1.00166000
H	-6.76655600	-1.56139100	0.34178500
H	-6.12701800	-2.50677400	1.71242700
H	-5.74547400	-0.76758800	1.57397000
C	-4.63276400	-3.69565600	-0.98321000
H	-3.73532600	-3.95448100	-1.56627000
H	-4.86325400	-4.53801400	-0.30998000
H	-5.47783600	-3.57836300	-1.68197400
C	4.63276100	-3.69565500	0.98321400
H	4.86324800	-4.53801400	0.30998500
H	5.47783400	-3.57836300	1.68197700
H	3.73532200	-3.95447600	1.56627500
C	5.89348100	-1.69661500	-1.00165700
H	6.76655700	-1.56139200	-0.34178000
H	6.12702200	-2.50677800	-1.71242100
H	5.74547900	-0.76759100	-1.57396900

compound **5** (dianion, singlet)

C	-2.00987400	-1.02748900	-0.62601900
C	0.86599400	-0.62367200	1.35739500
C	-1.54981800	0.45960100	1.40021400
C	-2.40568400	-0.40534100	0.67244400
C	-2.89840300	-2.02989600	-1.10752700
C	-0.86599600	-0.62367300	-1.35739500
C	-0.66755400	2.03415100	-1.44980500
C	1.54981700	0.45959900	-1.40021500
C	0.66755400	2.03415100	1.44980500
C	2.40568400	-0.40534100	-0.67244300
C	-3.68458500	-0.79242700	1.15876800

C	0.05959300	3.24902700	-1.54297900
H	1.12470300	3.23934500	-1.29906500
C	-2.04805500	2.14497200	-1.75451700
H	-2.67177500	1.24877600	-1.70385000
C	-4.16249300	-0.25965500	2.36286400
H	-5.14728700	-0.54480900	2.74832200
C	-2.08161300	0.96493200	2.60606400
H	-1.45946700	1.63243800	3.20998200
C	-0.60884900	-1.35091300	-2.53915000
H	0.27500500	-1.08771100	-3.12858700
C	-2.60442000	-2.70886700	-2.29575500
C	-3.35983600	0.63676300	3.08095600
H	-3.71916500	1.06137400	4.02442400
C	2.08161100	0.96492800	-2.60606600
H	1.45946400	1.63243300	-3.20998500
C	2.04805300	2.14496600	1.75452500
H	2.67176900	1.24876600	1.70386500
C	3.68458400	-0.79242900	-1.15876800
C	-1.43824200	-2.38235000	-3.00169200
H	-1.18668800	-2.91631200	-3.92419200
C	-0.52599500	4.46306400	-1.90990200
H	0.08849400	5.36870200	-1.95686300
C	-0.05958700	3.24903200	1.54297200
H	-1.12469600	3.23935600	1.29905400
C	-2.65410300	3.35560500	-2.11141700
H	-3.72730900	3.38033600	-2.32981200
C	2.65410600	3.35559700	2.11142400
H	3.72731100	3.38032200	2.32982400
C	0.52600600	4.46306600	1.90989700
H	-0.08847900	5.36870700	1.95685400
C	-1.89738400	4.53285800	-2.19814900
H	-2.36441100	5.48093400	-2.48108800
C	-5.99467900	-1.59298200	-0.85938000
H	-5.86206200	-0.62327300	-1.36468400
H	-6.82783500	-1.49746300	-0.14277800
H	-6.28328900	-2.34009000	-1.61796600
B	-0.01724200	0.68086800	0.91376400
C	1.89739400	4.53285400	2.19815000
H	2.36442400	5.48092800	2.48108900
B	0.01724100	0.68086700	-0.91376400
Si	-4.39867200	-2.10152400	0.01869700
C	-4.67722800	-3.76359300	0.87590500
H	-5.48503000	-3.68920900	1.62349000
H	-4.96049100	-4.54182400	0.14725900

H	-3.76027900	-4.09051100	1.39073000
C	2.00987300	-1.02748800	0.62602000
C	0.60884700	-1.35091300	2.53915000
H	-0.27500800	-1.08771100	3.12858600
C	1.43824000	-2.38235100	3.00169200
H	1.18668400	-2.91631300	3.92419100
C	2.60441800	-2.70886700	2.29575500
H	3.27604000	-3.48628400	2.67581800
C	2.89840200	-2.02989500	1.10752900
C	3.35983300	0.63675700	-3.08096000
H	3.71916100	1.06136500	-4.02442800
C	4.16249100	-0.25966000	-2.36286600
H	5.14728300	-0.54481600	-2.74832600
Si	4.39867100	-2.10152500	-0.01869700
C	5.99467900	-1.59298500	0.85937800
H	6.28329500	-2.34009900	1.61795600
H	6.82783200	-1.49745900	0.14277300
H	5.86206300	-0.62328100	1.36469100
C	4.67722400	-3.76359600	-0.87590200
H	5.48502600	-3.68921400	-1.62348800
H	4.96048600	-4.54182500	-0.14725400
H	3.76027500	-4.09051300	-1.39072700
H	-3.27604300	-3.48628400	-2.67581700

compound **7** (anion, singlet)

C	2.18967600	1.23578300	1.21716100
C	-1.44598100	-1.41975500	-0.84458100
C	2.27948100	2.76225100	-1.00487100
C	2.35526800	0.02927500	-1.19386700
C	-2.82951700	-1.35927800	-0.62925300
C	1.61405600	-1.78712600	0.53797200
C	0.42021800	-1.26281200	1.10456000
C	2.31314400	-1.30691000	-0.70538800
C	-2.07618400	0.00122300	1.17055500
C	-1.05208700	-2.20132500	-1.93829400
H	0.00547400	-2.32188500	-2.19336600
C	2.22253100	-2.93900900	1.11879900
C	3.11633100	-2.31167900	-1.32699000
C	1.31502000	1.55598000	2.26820100
H	0.28913700	1.83916500	2.04014200
C	1.72226000	3.26721600	-2.19902200
H	0.94634400	2.69128000	-2.70959500
C	3.26512400	3.56162800	-0.39485000
H	3.72239200	3.22423800	0.53743200

C	-3.18726900	-0.54991600	0.51549800
C	0.05936900	-1.76273400	2.37535900
H	-0.82598900	-1.34916300	2.86030700
C	3.03579600	0.23187500	-2.41460300
H	3.07495500	1.24049600	-2.82766700
C	3.48809100	0.83175500	1.58619700
H	4.19859800	0.55147800	0.80362500
C	3.78516800	-2.05549100	-2.52458200
H	4.39364200	-2.83400800	-2.99601300
C	0.74763200	-2.79140100	3.02238800
H	0.42410300	-3.13046100	4.01059500
C	3.70335500	-0.78386800	-3.10221200
H	4.20779500	-0.56643300	-4.04788400
C	1.79871100	-3.42901200	2.35624000
H	2.27610800	-4.31013800	2.79665200
C	-3.85140900	-1.95469400	-1.38138600
C	-2.03055500	-2.84168900	-2.73268500
H	-1.70515400	-3.44758000	-3.58358500
C	3.89270300	0.75166000	2.92262800
H	4.90736200	0.42408700	3.16672300
B	-0.74715600	-0.47583900	0.32152000
B	1.82505900	1.33502100	-0.35743600
C	-3.41251900	-2.72347100	-2.47401800
H	-4.12073000	-3.23629300	-3.13260100
C	2.11787000	4.48850400	-2.75166300
H	1.65352000	4.84309300	-3.67627900
C	-2.39546800	0.80859600	2.27134000
H	-1.61616900	1.29882500	2.86068100
C	2.99663300	1.07812600	3.94767700
H	3.30222500	1.00875700	4.99509000
C	-4.54667800	-0.38206600	0.81545600
C	3.67655300	4.78611200	-0.93789800
H	4.44539200	5.37440100	-0.42876400
C	1.70000000	1.48015100	3.61018400
H	0.97855900	1.72074000	4.39631700
C	3.10299000	5.25931000	-2.12191100
H	3.41519200	6.21691100	-2.54715400
C	-3.74320700	1.01665700	2.63462400
H	-3.96465700	1.65552500	3.49444300
C	-4.81496900	0.43810800	1.92376300
H	-5.83990900	0.64387100	2.24824100
Si	3.35322900	-3.74361300	-0.13986100
Si	-5.47234400	-1.38380800	-0.53217400
C	-6.53075200	-0.28931300	-1.64470800

H	-6.93253300	-0.86579500	-2.49434500
H	-7.38171100	0.13209700	-1.08419500
H	-5.93451700	0.54529200	-2.04572700
C	-6.50482300	-2.80122600	0.15808800
H	-5.89421200	-3.44608800	0.80926700
H	-7.35219500	-2.41571300	0.74903900
H	-6.91166200	-3.42057700	-0.65847000
C	2.67887000	-5.40949700	-0.71278300
H	3.25911600	-5.79543400	-1.56740000
H	2.73281200	-6.15441700	0.09858300
H	1.62688200	-5.31254300	-1.02294500
C	5.16106300	-3.92617400	0.36613700
H	5.26812700	-4.66150200	1.18092200
H	5.77423200	-4.26987700	-0.48365300
H	5.56297300	-2.96222900	0.71484600
C	-3.26472200	3.65976900	-0.26737000
C	-2.72766500	4.75973800	0.41304300
C	-1.36970800	4.76754300	0.74637200
C	-0.55092700	3.68057500	0.42012300
C	-1.09416300	2.57459500	-0.24443700
C	-2.45116400	2.57792300	-0.60484100
H	-4.32306600	3.64227600	-0.53769800
H	-3.36367400	5.60825500	0.67632800
H	-0.93746300	5.62494000	1.26818400
H	0.50526200	3.70002100	0.67996300
H	-2.87179700	1.72275200	-1.13503000
S	-0.12409600	1.14901100	-0.73299000

PhSSPh (neutral, singlet)

C	-3.47158800	0.62676600	0.72187600
C	-3.13691900	1.84485300	0.11541200
C	-2.06166300	1.91152500	-0.77568900
C	-1.31597400	0.76343200	-1.06163300
C	-1.65347100	-0.45612600	-0.45570400
C	-2.73397400	-0.52425200	0.43814700
H	-4.31298300	0.57381500	1.41674100
H	-3.71718500	2.74291100	0.33971400
H	-1.79662800	2.86055100	-1.24728200
H	-0.46823000	0.80947500	-1.74725700
H	-2.98692300	-1.47586800	0.90967000
S	-0.67804400	-1.90693900	-0.82271900
C	2.06123500	1.91171600	0.77544200
C	3.13678500	1.84506800	-0.11532300
C	3.47183200	0.62694300	-0.72147700

C	2.73428000	-0.52414200	-0.43780500
C	1.65349600	-0.45603300	0.45569800
C	1.31564300	0.76356700	1.06135700
H	1.79592600	2.86078800	1.24679100
H	3.71696800	2.74319300	-0.33956800
H	4.31344700	0.57398800	-1.41607500
H	2.98749900	-1.47578400	-0.90912900
H	0.46769200	0.80957900	1.74673100
S	0.67819000	-1.90697100	0.82258600

$PhSSPh^{-}$ (anion, doublet)

C	-2.50125000	1.14883900	1.05843400
C	-2.10064000	2.13312100	0.14595600
C	-1.43127900	1.75099800	-1.02337000
C	-1.17078200	0.40583700	-1.28200100
C	-1.57626800	-0.60214900	-0.37773500
C	-2.23881500	-0.19828300	0.80474900
H	-3.01639600	1.43413600	1.98000200
H	-2.29861600	3.18835000	0.34962700
H	-1.09584000	2.51047100	-1.73409900
H	-0.63527500	0.11636300	-2.18772900
H	-2.54347300	-0.96215100	1.52334500
S	-1.24357400	-2.29526700	-0.70047800
C	1.32359600	1.82307100	1.02625600
C	1.96693400	2.24749000	-0.14292900
C	2.42795600	1.29088200	-1.05627900
C	2.24993600	-0.07052900	-0.80460100
C	1.61254400	-0.51637900	0.37690200
C	1.14735600	0.46391900	1.28295000
H	0.94158800	2.55919500	1.73805800
H	2.09780200	3.31325800	-0.34603100
H	2.92381300	1.60904200	-1.97769200
H	2.60049100	-0.81198200	-1.52593400
H	0.63201000	0.14079300	2.18894100
S	1.37720900	-2.22713100	0.69682300

PhS• (neutral, doublet)

C	-1.53771400	1.21791100	-0.00007000
C	-2.23676000	0.00009800	0.00019800
C	-1.53775000	-1.21783400	0.00005000
C	-0.14788700	-1.22399900	-0.00024500
C	0.57926900	-0.00008400	-0.00007000
C	-0.14773200	1.22381700	-0.00012700
H	-2.08657600	2.16216200	-0.00013500

H	-3.32915400	0.00011600	0.00042600
H	-2.08684200	-2.16196300	0.00018800
H	0.40517900	-2.16496400	-0.00052500
H	0.40527000	2.16482900	-0.00019600
S	2.30397300	0.00002300	0.00011400

PhS^{-} (anion, singlet)

C	-1.55924000	1.20429700	0.00000600
C	-2.27643400	0.00000400	-0.00001400
C	-1.55924500	-1.20429300	-0.00000500
C	-0.16206000	-1.20497500	0.00001800
C	0.58473200	-0.00000500	0.00000900
C	-0.16205500	1.20496900	0.00000900
H	-2.09508500	2.15866700	0.00001400
H	-3.36930100	0.00000700	-0.00003600
H	-2.09509500	-2.15866100	-0.00001700
H	0.37893100	-2.15476400	0.00004600
H	0.37894800	2.15475100	0.00001000
S	2.35046400	0.00000100	-0.00000900

TS-1 (anion, doublet)

C	0.52829600	1.72546400	1.03565000
C	3.28601400	-0.04322600	0.84892200
C	1.67296700	-0.90135000	2.89177200
C	1.77983600	-2.24373100	0.53796700
C	3.36500300	0.37074100	-0.50429400
C	-0.77701900	-2.05405500	0.63982300
C	-0.87574000	-0.64704500	0.79958200
C	0.48653300	-2.78050000	0.33740900
C	0.89258100	0.04212500	-1.11015600
C	4.42404400	0.18956100	1.65148700
H	4.40725700	-0.12322700	2.69752100
C	-1.92654400	-2.88807900	0.72038700
C	0.31469500	-4.11112900	-0.13185300
C	1.37526900	2.71538300	0.47687100
H	1.79979600	2.55265500	-0.51379300
C	1.94043400	0.21929300	3.70934500
H	2.43922700	1.08596500	3.27614300
C	0.99319300	-1.98190700	3.50149900
H	0.76697700	-2.87031000	2.90980100
C	2.24617800	0.23364100	-1.47360200
C	-2.13212300	-0.15773100	1.22354100
H	-2.25405700	0.91340100	1.36463400
C	2.87131700	-3.01741900	0.08913500

H	3.88125400	-2.61374000	0.19625700
C	-0.01208600	2.02348500	2.30872900
H	-0.64686100	1.28862100	2.80280100
C	1.42701600	-4.85881900	-0.52812300
H	1.30458000	-5.88327900	-0.89305300
C	-3.24518000	-0.97923200	1.39854300
H	-4.20167600	-0.54182800	1.69598000
C	2.70812700	-4.29631800	-0.45243100
H	3.58175200	-4.86545400	-0.78149300
C	-3.15425200	-2.34587800	1.10522400
H	-4.04733900	-2.97481400	1.15972300
C	4.57552600	0.86906900	-1.05304400
C	5.59740500	0.75446700	1.14095300
H	6.45822000	0.90929800	1.79699600
C	0.26920100	3.21473300	2.97919700
H	-0.17339000	3.39875900	3.96186800
B	0.24380700	0.35596300	0.31024300
B	2.11814400	-0.95067700	1.38716500
C	5.68181700	1.06892600	-0.22179000
H	6.62092400	1.45295600	-0.63204200
C	1.55589200	0.26496200	5.05089200
H	1.76986400	1.15552900	5.64775100
C	0.01467300	-0.32687900	-2.15404200
H	-1.02365400	-0.54554700	-1.90409600
C	1.12935000	4.15881300	2.41028700
H	1.35855300	5.08996600	2.93502700
C	2.64085400	0.30272100	-2.83901700
C	0.60098900	-1.94691700	4.84061000
H	0.07510500	-2.80001400	5.27756000
C	1.68039400	3.89837800	1.14651800
H	2.34541500	4.62971200	0.67864100
C	0.88199300	-0.81933100	5.62466900
H	0.57570000	-0.78683300	6.67374400
C	0.40875900	-0.35944200	-3.49200400
H	-0.31528400	-0.62679100	-4.26666800
C	1.71539400	0.01197300	-3.84336300
H	2.00292900	0.06389500	-4.89793600
Si	-1.49018100	-4.59996100	0.07849600
Si	4.40684900	0.94632700	-2.92759100
C	4.48812900	2.68487800	-3.65130700
H	5.50122400	3.10715800	-3.54623500
H	4.23185600	2.67566700	-4.72363200
H	3.77955500	3.34979500	-3.13344000
C	5.63603900	-0.18302000	-3.80160300

H	5.58747800	-1.20229200	-3.38802200
H	5.41789000	-0.23646700	-4.88108600
H	6.66629000	0.19138900	-3.68180000
C	-2.38109700	-5.02408500	-1.52795100
H	-2.02195600	-5.98615500	-1.92914200
H	-3.46853300	-5.10790200	-1.36720000
H	-2.19915700	-4.24657400	-2.28526600
C	-1.70963000	-5.99728900	1.32497000
H	-2.77786400	-6.16086100	1.54419500
H	-1.29434500	-6.94122800	0.93476100
H	-1.19622400	-5.75718400	2.26903900
C	-2.11806700	5.37861600	1.20491600
C	-3.26802800	5.06868300	1.93906100
C	-3.96563600	3.88496400	1.66258200
C	-3.51075400	3.01457500	0.67094800
C	-2.35556400	3.32341300	-0.07355700
C	-1.67078700	4.52038900	0.19806400
H	-1.56165200	6.29443700	1.41867700
H	-3.61871900	5.74290100	2.72424600
H	-4.86485700	3.63384200	2.23118500
H	-4.04789800	2.08869500	0.46579800
H	-0.77122700	4.75986800	-0.36959700
S	-1.71388400	2.20247600	-1.28392800
C	-6.10897700	-0.35499500	-1.02921700
C	-5.59315600	-1.62303700	-1.32175700
C	-4.43939600	-1.73512800	-2.10591500
C	-3.80444600	-0.59295400	-2.59314600
C	-4.30974900	0.68911700	-2.29687000
C	-5.47199900	0.79276300	-1.50803100
H	-7.00899900	-0.25748000	-0.41661700
H	-6.08350000	-2.51912100	-0.93484200
H	-4.02514800	-2.71948800	-2.32993300
H	-2.90693000	-0.68268000	-3.20663100
H	-5.86912800	1.78177600	-1.27154100
S	-3.47253300	2.14112400	-2.86900200

TS-2 (dianion, open-shell singlet)

C	-0.70626000	-1.55186100	1.47141300
C	-3.55578200	-0.14298700	0.53763400
C	-2.26872000	1.23202200	2.53103200
C	-2.15564300	2.06405500	-0.04876500
C	-3.44435200	-0.84267700	-0.69004800
C	0.37361600	2.14037100	0.34143000
C	0.52655700	0.80978500	0.80546500

C	-0.89335800	2.67756300	-0.22306000
C	-0.92826400	-0.50457500	-1.02699700
C	-4.78761000	-0.25981400	1.21892200
H	-4.92064800	0.27079200	2.16421800
C	1.46199800	3.05218100	0.33088800
C	-0.75921500	3.90012700	-0.93710800
C	-1.25031200	-2.78244300	1.03106200
H	-1.54164100	-2.89043000	-0.01451000
C	-2.62430200	0.30644900	3.54016200
H	-3.02462700	-0.66540600	3.24966900
C	-1.71724100	2.45802600	2.97558700
H	-1.42824000	3.20892900	2.23850100
C	-2.20501100	-0.86813300	-1.51187100
C	1.78094100	0.47771600	1.36397500
H	1.95692400	-0.54142100	1.70646800
C	-3.23808200	2.63127500	-0.75599700
H	-4.22192100	2.16193800	-0.67448700
C	-0.32456400	-1.48877100	2.82962100
H	0.08931900	-0.55888200	3.22022500
C	-1.86777500	4.44781700	-1.58850100
H	-1.77781600	5.38677400	-2.14371300
C	2.83846300	1.38866900	1.42101000
H	3.79823300	1.07011000	1.83291500
C	-3.10468200	3.79047400	-1.52725600
H	-3.97265700	4.19913200	-2.05239600
C	2.68691100	2.67260900	0.88615800
H	3.53646000	3.36138200	0.88056900
C	-4.55457200	-1.51990600	-1.26079800
C	-5.86770300	-0.98282400	0.70142000
H	-6.80585600	-1.03628700	1.26111800
C	-0.48069900	-2.56985200	3.69858200
H	-0.17329700	-2.47396200	4.74343300
B	-0.53342400	-0.32159900	0.50447600
B	-2.47589000	0.91312900	1.00360800
C	-5.75951400	-1.59349600	-0.55531400
H	-6.62314200	-2.11178400	-0.98379700
C	-2.44799600	0.58002100	4.89755800
H	-2.72796600	-0.16922300	5.64344800
C	0.11866400	-0.45812800	-1.97120000
H	1.11316600	-0.16893000	-1.63220100
C	-1.02581900	-3.77116400	3.23463900
H	-1.14547300	-4.62369400	3.90879900
C	-2.38552400	-1.32292300	-2.84730400
C	-1.52823200	2.74047900	4.33004400

H	-1.09446400	3.69848500	4.63030900
C	-1.40884400	-3.87112700	1.88835300
H	-1.82517200	-4.80757800	1.50699700
C	-1.89485300	1.80112500	5.30381900
H	-1.74867600	2.01794500	6.36549300
C	-0.06154800	-0.82681000	-3.30672100
H	0.78321800	-0.79158800	-3.99987900
C	-1.31005000	-1.29482800	-3.73920200
H	-1.43296000	-1.64212900	-4.76978600
Si	0.97713100	4.57359100	-0.65840100
Si	-4.12136200	-2.02340600	-3.02292300
C	-4.11552400	-3.88241900	-3.33658300
H	-5.13949500	-4.29077500	-3.31054500
H	-3.68258300	-4.11016900	-4.32479500
H	-3.51566900	-4.39965000	-2.57175100
C	-5.21251600	-1.15798500	-4.29493100
H	-5.19167000	-0.06754700	-4.14312800
H	-4.86665600	-1.37168800	-5.31992300
H	-6.25771300	-1.49975500	-4.21106200
C	2.01301500	4.79709900	-2.21777300
H	1.59328700	5.59464900	-2.85300200
H	3.04842100	5.07750700	-1.96321900
H	2.04146100	3.86723900	-2.80515100
C	0.95781500	6.18478900	0.32394300
H	1.97787600	6.46332900	0.63694800
H	0.55048400	7.01110100	-0.28220400
H	0.33646100	6.07920300	1.22692700
C	3.00516200	-3.05543200	3.39809600
C	4.09762800	-2.20969500	3.62773500
C	4.75485500	-1.64515900	2.52570700
C	4.33151900	-1.91165700	1.22271700
C	3.23983400	-2.77919700	0.97451600
C	2.58908200	-3.34429800	2.09751900
H	2.45923700	-3.48822700	4.24040100
H	4.42430600	-1.98503600	4.64620600
H	5.59874400	-0.96663300	2.68282200
H	4.83265000	-1.41012000	0.39479600
H	1.72122000	-3.98698500	1.93730500
S	2.66275700	-3.09804500	-0.65506300
C	5.93983900	1.22955800	-1.44495900
C	4.71947100	1.86128500	-1.71085200
C	3.67004800	1.10779500	-2.25267200
C	3.84268400	-0.24377000	-2.54283800
C	5.06660800	-0.90315700	-2.27847400

C	6.10919700	-0.13255700	-1.71417300
H	6.76761500	1.79872000	-1.01158300
H	4.57811500	2.91902700	-1.48337100
H	2.70088900	1.57901100	-2.43280400
H	3.01799800	-0.82725100	-2.95015600
H	7.05985900	-0.61791800	-1.48075300
S	5.25987800	-2.62222400	-2.59861900

INT-2 (anion, singlet)

C	-1.20140200	0.33543700	2.18537200
C	-2.29795800	1.42662500	-0.52786500
C	-0.23970700	3.21413000	-0.12633100
C	0.10237400	1.07111300	-1.71042200
C	-2.72465500	0.07619400	-0.64675500
C	2.05803800	0.71801300	-0.11221300
C	1.36562100	0.51820900	1.10810200
C	1.44566600	0.71172500	-1.46534900
C	-0.53080800	-1.16573800	-0.03827000
C	-3.30352600	2.42104700	-0.53527000
H	-3.01248200	3.46995800	-0.46548400
C	3.47248300	0.91418000	-0.13891200
C	2.33361900	0.43547500	-2.53710600
C	-2.43106500	-0.33621400	2.36803000
H	-2.60165800	-1.27933500	1.84667400
C	-0.86816700	3.92633400	0.91712400
H	-1.76036000	3.51154600	1.38210400
C	0.92369000	3.79533900	-0.67892000
H	1.43920100	3.28501000	-1.49346200
C	-1.80846600	-1.09858400	-0.64828300
C	2.09723700	0.86144000	2.27200500
H	1.61345000	0.73837500	3.24131800
C	-0.37247500	0.93457800	-3.03059500
H	-1.41948500	1.16628400	-3.24340900
C	-1.03627500	1.52661800	2.91184300
H	-0.10731300	2.08710300	2.81120700
C	1.83449400	0.34754800	-3.84015200
H	2.50538200	0.13292100	-4.67768500
C	3.43820200	1.24241400	2.25076300
H	3.94860600	1.49293700	3.18488200
C	0.46806700	0.55250400	-4.08135200
H	0.06802600	0.46220200	-5.09473600
C	4.15531900	1.18880800	1.04328700
H	5.23743300	1.35218400	1.04091000
C	-4.08378400	-0.22594100	-0.93115900

C	-4.65725700	2.11822100	-0.69750600
H	-5.40146300	2.91880800	-0.69928300
C	-2.03885700	2.04427400	3.74225900
H	-1.86930600	2.98452800	4.27517500
B	-0.02887700	-0.31487300	1.26789000
B	-0.80080100	1.84862900	-0.68209300
C	-5.04056900	0.79132800	-0.93237200
H	-6.08923800	0.56158600	-1.14608300
C	-0.35990600	5.13464400	1.39909600
H	-0.86651600	5.65048200	2.21897800
C	0.30307800	-2.20040100	-0.52204800
H	1.33810200	-2.23132100	-0.18926500
C	-3.25619100	1.37230700	3.87677800
H	-4.04682200	1.77503700	4.51586800
C	-2.33039600	-2.20880500	-1.38157400
C	1.43947300	5.00562800	-0.21035900
H	2.34403400	5.42351000	-0.65943900
C	-3.44482800	0.16867500	3.18338000
H	-4.38761600	-0.37720600	3.28044300
C	0.79994400	5.67956900	0.83667700
H	1.20310000	6.62407500	1.21174100
C	-0.13941200	-3.20913200	-1.37540300
H	0.55632900	-3.98579700	-1.70515800
C	-1.49477000	-3.26530800	-1.73759500
H	-1.88013800	-4.11852800	-2.30437300
Si	4.09375300	0.52022200	-1.87279600
Si	-4.18929800	-1.99928400	-1.55061800
C	-5.12805500	-3.18311700	-0.42254100
H	-6.20258300	-2.93696900	-0.39934400
H	-5.02359500	-4.22354100	-0.77239000
H	-4.73608400	-3.12475600	0.60478200
C	-4.87575400	-2.09325200	-3.30323700
H	-4.33174800	-1.40670500	-3.97037100
H	-4.77807800	-3.11557400	-3.70488500
H	-5.94459200	-1.82258600	-3.32309200
C	5.03372300	-1.11345800	-1.90051800
H	5.26544600	-1.41826700	-2.93439200
H	5.98392300	-1.03031500	-1.34717900
H	4.43008700	-1.90479000	-1.43050000
C	5.10084400	1.87419900	-2.71183200
H	6.06855900	2.01399700	-2.20206100
H	5.30603500	1.61616000	-3.76407100
H	4.55714900	2.83155600	-2.69002900
C	4.00322800	-3.26702300	1.31659800

C	3.78656000	-4.64996700	1.27090300
C	2.54529800	-5.15621200	1.67433600
C	1.53353000	-4.29051900	2.10106000
C	1.73973000	-2.89754300	2.14796700
C	3.00025400	-2.40096100	1.75880500
H	4.96746100	-2.85416300	1.00862100
H	4.57436500	-5.32387900	0.92531500
H	2.35620300	-6.23288300	1.64398300
H	0.55766200	-4.69057300	2.38538900
H	3.19179000	-1.33120700	1.78956700
S	0.40847500	-1.84488700	2.69536600

TS-3 (anion, singlet)

C	0.80795600	1.00926400	1.76784000
C	-0.39823300	2.42756500	-0.19906600
C	2.31659200	2.50956500	0.27459000
C	1.26892100	0.53819300	-1.13586400
C	-1.63980400	1.84849600	-0.57661600
C	1.89466300	-1.72670600	-0.02753800
C	0.99737100	-1.71507900	1.06707200
C	1.86591800	-0.74807700	-1.15748700
C	-1.11684800	-0.67029100	-0.22257900
C	-0.35857900	3.83801500	-0.18091700
H	0.57763800	4.33117900	0.07788000
C	2.83015800	-2.79109700	-0.17395300
C	2.52320100	-1.22836100	-2.33233600
C	-0.07447100	1.74451900	2.59808400
H	-0.98582300	2.15025400	2.16123100
C	2.43129800	3.46459000	1.31000600
H	1.60890800	3.58149400	2.01943400
C	3.44381500	2.38977700	-0.56278800
H	3.43704300	1.66098400	-1.37349400
C	-1.88426500	0.38302700	-0.76915900
C	1.16984300	-2.69967900	2.05789300
H	0.50261800	-2.68000400	2.92113100
C	1.16912400	1.18029900	-2.39333900
H	0.68241300	2.15701200	-2.43341600
C	1.98359800	0.53151600	2.41500900
H	2.71998200	-0.01064200	1.82257400
C	2.43764100	-0.52593000	-3.53416100
H	2.93584900	-0.89847700	-4.43471900
C	2.14552400	-3.69420200	1.96613400
H	2.24627200	-4.43890900	2.76049200
C	1.70156900	0.66540700	-3.57491700

H	1.58565700	1.21589900	-4.51300000
C	2.95827600	-3.75752900	0.82620500
H	3.67714800	-4.57559000	0.71514700
C	-2.73638600	2.66988100	-0.98052000
C	-1.44994500	4.65057700	-0.49939100
H	-1.35058700	5.73918900	-0.45799100
C	2.25054000	0.75041100	3.76017400
H	3.17306800	0.36965800	4.20505200
B	-0.22537100	-0.70367600	1.09723400
B	0.95274700	1.55028800	0.12404500
C	-2.64726900	4.06115200	-0.91693800
H	-3.49084500	4.68703200	-1.22545100
C	3.56448100	4.26160000	1.48584800
H	3.59578100	4.98914400	2.30249200
C	-1.36739900	-1.96695700	-0.73165400
H	-0.76249000	-2.79622100	-0.36356200
C	1.33452100	1.47113900	4.54306800
H	1.53451600	1.64355400	5.60405000
C	-2.99663500	0.08443500	-1.61015200
C	4.59642900	3.17224300	-0.39383600
H	5.44581700	3.03344200	-1.06940700
C	0.16893600	1.96815100	3.95625500
H	-0.55303100	2.52946500	4.55425400
C	4.66438400	4.12096600	0.62885600
H	5.55911500	4.73492000	0.76381000
C	-2.38446700	-2.24723600	-1.64259500
H	-2.53696900	-3.27166800	-1.99181600
C	-3.24395000	-1.21940400	-2.04026500
H	-4.10449300	-1.43811200	-2.68010600
Si	3.53540000	-2.75126800	-1.91325500
Si	-4.04387100	1.62155200	-1.82039700
C	-5.68408400	1.47080700	-0.89731500
H	-6.23233700	2.42737000	-0.90442500
H	-6.32577800	0.70708500	-1.36765600
H	-5.51375100	1.17857600	0.15030400
C	-4.36448100	2.15801600	-3.59931700
H	-3.41782900	2.22929000	-4.15715700
H	-5.01744800	1.43495300	-4.11584600
H	-4.85938600	3.14309600	-3.62958500
C	3.09552700	-4.30381700	-2.88934300
H	3.38012500	-4.19429100	-3.94904000
H	3.62214500	-5.18473800	-2.48586400
H	2.01230800	-4.49526200	-2.84072000
C	5.39089000	-2.43003600	-2.02323200

H	5.96300700	-3.28413800	-1.62433000
H	5.69855600	-2.27432200	-3.07074600
H	5.66172800	-1.53164600	-1.44705100
C	-3.54204400	-4.21465000	1.61309100
C	-4.71128600	-3.68401900	1.05584300
C	-4.87760600	-2.29612400	0.99208500
C	-3.88275200	-1.44606200	1.47881000
C	-2.70385100	-1.97103200	2.02984200
C	-2.54383400	-3.36384200	2.09777700
H	-3.40275300	-5.29765600	1.66572400
H	-5.48736300	-4.34963300	0.66994500
H	-5.78086600	-1.87047100	0.54795000
H	-3.99903800	-0.36435300	1.40875500
H	-1.62649300	-3.77927400	2.51738900
S	-1.44428900	-0.85263200	2.64233100

INT-3 (anion, singlet)

C	1.39386900	1.16108000	1.85763800
C	-1.14959700	1.23958500	0.71658100
C	0.63024100	3.09592200	0.18341800
C	1.10873700	0.73447800	-0.87423000
C	-2.00856500	0.51267700	-0.14360900
C	2.50735500	-1.25042900	0.05197200
C	1.62018200	-1.76771000	1.02855200
C	2.12895300	-0.24781500	-0.98344800
C	-0.61580300	-1.59037800	-0.51488600
C	-1.81382300	2.15346500	1.57168200
H	-1.21511200	2.73124900	2.27975600
C	3.82122400	-1.77994000	-0.06686200
C	2.93123500	-0.35628600	-2.16347300
C	0.84034100	0.99874500	3.14096500
H	-0.24310900	0.97656100	3.25299200
C	-0.38182100	3.80075200	-0.50933100
H	-1.30152400	3.28059200	-0.78445600
C	1.76602300	3.85837500	0.51910600
H	2.58042500	3.39101800	1.07366800
C	-1.62884100	-0.69641400	-0.91656800
C	2.15461300	-2.64925800	1.99452100
H	1.49377400	-3.05117000	2.76574200
C	0.73004200	1.31235200	-2.11013700
H	-0.11052200	2.00501800	-2.12243900
C	2.80576200	1.15166700	1.80345300
H	3.29966900	1.26263000	0.83637800
C	2.55835700	0.30826400	-3.32912200

H	3.15932800	0.22547300	-4.24008400
C	3.48686700	-3.06461700	1.96432800
H	3.86680800	-3.74538800	2.73033900
C	1.39005300	1.08529100	-3.31677400
H	1.03316500	1.56614600	-4.23233500
C	4.30556200	-2.66546300	0.89792000
H	5.31921700	-3.07031300	0.81503600
C	-3.35164100	0.93532400	-0.38610000
C	-3.17566600	2.44521600	1.48270000
H	-3.62576900	3.16645900	2.17132100
C	3.60845200	0.97819600	2.93347900
H	4.69743700	0.96336300	2.82970400
B	0.08007600	-1.58625400	0.88495400
B	0.49049500	1.44463300	0.51656000
C	-3.93293000	1.89836500	0.43474000
H	-4.96395100	2.22718600	0.27149500
C	-0.26631600	5.14947100	-0.85900100
H	-1.08124700	5.64109700	-1.39914400
C	-0.31895000	-2.67043100	-1.37095600
H	0.48849800	-3.35556700	-1.09971400
C	3.02029700	0.80988300	4.19358800
H	3.63922500	0.66524500	5.08354000
C	-2.43671600	-0.97991100	-2.04823600
C	1.89949600	5.21327000	0.18241500
H	2.80452900	5.75562300	0.47300000
C	1.62535000	0.82454600	4.28828500
H	1.14164400	0.68843300	5.26041500
C	0.88282500	5.87218900	-0.51469100
H	0.97919300	6.92883100	-0.77948600
C	-1.05145100	-2.90877200	-2.53720600
H	-0.79963200	-3.75956000	-3.17578000
C	-2.13805500	-2.08045000	-2.85531200
H	-2.75337700	-2.30872900	-3.73122100
Si	4.50034300	-1.30358000	-1.75424800
Si	-3.91344600	0.18747000	-2.01906400
C	-5.57395100	-0.69829300	-2.06439100
H	-6.39568300	0.01038200	-1.86800000
H	-5.74377000	-1.14602700	-3.05774400
H	-5.61461700	-1.49444000	-1.30844100
C	-3.87112700	1.50407900	-3.37226800
H	-2.89862200	2.02045100	-3.36942200
H	-4.02440300	1.05604400	-4.36797200
H	-4.66098200	2.25652600	-3.21029800
C	4.85225900	-2.81679400	-2.82212200

H	5.09313900	-2.51657500	-3.85550500
H	5.70866400	-3.38818400	-2.42693900
H	3.97518700	-3.48187600	-2.85142300
C	6.00981400	-0.17331200	-1.72084700
H	6.89272200	-0.70557200	-1.32966500
H	6.25077400	0.18832900	-2.73426000
H	5.81990600	0.70135200	-1.07954900
C	-4.88360300	-1.10243900	2.23547300
C	-5.35508100	-2.11574200	1.39661400
C	-4.45079300	-3.01795400	0.82226800
C	-3.08556200	-2.91773200	1.09620200
C	-2.61518400	-1.90384000	1.94331300
C	-3.51700700	-0.99366500	2.50755500
H	-5.57844800	-0.38014900	2.66960900
H	-6.42291400	-2.19692000	1.18035000
H	-4.80983200	-3.80625100	0.15613900
H	-2.38179400	-3.62016500	0.64856500
H	-3.14440000	-0.18587800	3.13823700
S	-0.89222200	-1.79592900	2.41270100

TS-4 (anion, singlet)

C	1.69135600	1.80583800	1.21890800
C	-0.96848600	0.71577300	0.78806500
C	-0.10573300	2.95404100	-0.38673200
C	1.21511200	0.68006800	-1.16487000
C	-1.88090300	0.15239800	-0.14186500
C	2.57309600	-0.96215800	0.29610500
C	1.66263000	-1.14975500	1.36236300
C	2.23173700	-0.29986600	-0.99646700
C	-0.41367600	-1.73652200	-0.25608200
C	-1.60484600	1.62825700	1.68644700
H	-1.02198600	2.10743100	2.47353200
C	3.89471200	-1.48229300	0.36731400
C	3.10260800	-0.65831200	-2.06396500
C	1.56675600	1.61669300	2.60557100
H	0.66426000	1.15550700	3.00673500
C	-1.16184500	3.03844200	-1.31892400
H	-1.56059100	2.12467200	-1.75227900
C	0.31348900	4.18503700	0.15960900
H	1.11733800	4.19557500	0.89687200
C	-1.48892700	-1.05692500	-0.83146200
C	2.19650800	-1.67456300	2.55690500
H	1.54378900	-1.79996800	3.42287600
C	1.00109300	1.09136400	-2.49732300

H	0.23630800	1.83558300	-2.70492400
C	2.91936500	2.34150500	0.77740500
H	3.07075500	2.49875300	-0.29387700
C	2.85171600	-0.20306900	-3.36032400
H	3.51215700	-0.48105300	-4.18787700
C	3.53020200	-2.07023700	2.68293600
H	3.89534200	-2.46285900	3.63582800
C	1.75717300	0.63698500	-3.58378200
H	1.52680700	0.99329500	-4.59198500
C	4.37036300	-2.02395000	1.56374300
H	5.38819000	-2.42160500	1.62779000
C	-3.19981400	0.59710400	-0.40327100
C	-2.92508800	2.03678600	1.52353500
H	-3.34610100	2.77103700	2.21539500
C	3.95955800	2.66182500	1.65398400
H	4.89906100	3.06298200	1.26235100
B	0.08831000	-1.07481600	1.10894800
B	0.55088500	1.51116400	0.09372500
C	-3.71130600	1.57557600	0.44242300
H	-4.71966800	1.97656300	0.30367600
C	-1.75126700	4.24725400	-1.69547700
H	-2.56963500	4.24720600	-2.42163200
C	-0.03671200	-2.93392200	-0.88462200
H	0.80931600	-3.51471000	-0.50607500
C	3.80099300	2.46452200	3.03132800
H	4.60998400	2.70819100	3.72543200
C	-2.29979200	-1.52853200	-1.88330200
C	-0.26818600	5.40849900	-0.20008400
H	0.09464100	6.33399600	0.25693300
C	2.59378900	1.93777400	3.50085000
H	2.45582400	1.75590200	4.57073400
C	-1.30646700	5.45079500	-1.13545400
H	-1.76427100	6.40226300	-1.41973100
C	-0.77270600	-3.41728800	-1.97897600
H	-0.47904100	-4.36292300	-2.44351700
C	-1.91075600	-2.73857500	-2.46726500
H	-2.48304900	-3.18195400	-3.28778900
Si	4.61964600	-1.51592800	-1.36444900
Si	-3.76700000	-0.32031300	-1.97699500
C	-5.47178800	-1.11256300	-1.94504200
H	-6.24985200	-0.33605100	-1.85818000
H	-5.64989000	-1.66639100	-2.88166900
H	-5.57842700	-1.80561200	-1.10056100
C	-3.65736100	0.83127500	-3.47015800

H	-2.64688700	1.25613400	-3.56668200
H	-3.88894300	0.28375300	-4.39861800
H	-4.37430400	1.66342200	-3.37473800
C	4.90948100	-3.27969900	-1.96748000
H	5.18170800	-3.28907100	-3.03603300
H	5.72758600	-3.75896200	-1.40434400
H	3.99891300	-3.88517300	-1.83721800
C	6.18074300	-0.48751600	-1.61463800
H	7.03211700	-0.92466400	-1.06685600
H	6.45206400	-0.43938600	-2.68244800
H	6.02535100	0.53993500	-1.25070600
C	-4.84368000	-1.06566800	2.68372600
C	-5.31902700	-2.01354600	1.77345300
C	-4.41290500	-2.85582300	1.11666200
C	-3.04406700	-2.75000400	1.36386400
C	-2.56142800	-1.79564600	2.27519200
C	-3.47261000	-0.95811700	2.93455400
H	-5.54131100	-0.39916000	3.19704700
H	-6.39030700	-2.09634200	1.57451400
H	-4.77334600	-3.60204800	0.40448700
H	-2.34165400	-3.40627400	0.84981900
H	-3.09942200	-0.21202500	3.63653800
S	-0.83090400	-1.69749300	2.69186100

INT-4 (anion, singlet)

C	2.22995000	2.15953900	0.94187500
C	-1.12354500	0.30793900	1.26954000
C	0.17105300	3.13782400	-0.48843200
C	1.48401500	0.87062500	-1.32582100
C	-2.04460000	0.22056100	0.21115600
C	2.47519200	-0.91428500	0.25533800
C	1.54450100	-0.92166300	1.32453100
C	2.24550100	-0.30889200	-1.08406200
C	-0.53626200	-1.49498400	-0.41714700
C	-1.45753400	1.26232300	2.24045400
H	-0.84028000	1.39563500	3.13377700
C	3.73688600	-1.56348200	0.37533100
C	2.99184700	-0.91213300	-2.13161100
C	1.81548800	2.58951600	2.21810800
H	0.75565500	2.77177900	2.39508500
C	-1.00819400	2.96853000	-1.24229100
H	-1.23933400	1.98410200	-1.64471600
C	0.42111600	4.43343300	0.01892400
H	1.32895400	4.61382200	0.59682200

C	-1.70198300	-0.79724000	-0.75540100
C	2.08422900	-1.25579100	2.58586300
H	1.43097200	-1.21383500	3.46049900
C	1.29711200	1.22844100	-2.68391800
H	0.71593300	2.12045000	-2.91982900
C	3.61081500	1.95184800	0.75159900
H	3.97270500	1.62273700	-0.22449100
C	2.79236600	-0.50477400	-3.45214800
H	3.35464100	-0.97253600	-4.26648300
C	3.39387900	-1.71566200	2.75779000
H	3.75732400	-1.96213300	3.75943900
C	1.89195300	0.53032700	-3.73703100
H	1.71519800	0.84303200	-4.76928000
C	4.19525700	-1.95883600	1.63332200
H	5.16496900	-2.45336100	1.74617000
C	-3.21581000	0.96301700	0.00912600
C	-2.61354700	2.05847000	2.09236800
H	-2.84069100	2.80669600	2.85765000
C	4.53155800	2.14797600	1.78447300
H	5.59621300	1.97823400	1.60449700
B	-0.05748700	-0.93060000	1.05530500
B	1.21025000	1.97943100	-0.24869500
C	-3.48992700	1.92172000	0.99609400
H	-4.36890700	2.57119300	0.93566500
C	-1.89697100	4.02167100	-1.46401400
H	-2.81299900	3.85123300	-2.03110400
C	-0.17054900	-2.49298700	-1.32958800
H	0.72659600	-3.09943700	-1.16942300
C	4.08811000	2.53995400	3.05239600
H	4.80303700	2.67533100	3.86824500
C	-2.54263400	-0.99011200	-1.86030200
C	-0.45225900	5.49965300	-0.21082800
H	-0.22253400	6.49054500	0.18946400
C	2.72242800	2.75980200	3.26653100
H	2.36551600	3.06774900	4.25295600
C	-1.62128000	5.29367600	-0.95122900
H	-2.31534600	6.11972400	-1.12757500
C	-0.95986600	-2.73110800	-2.47532100
H	-0.65056900	-3.51133600	-3.17760700
C	-2.13667600	-2.00026700	-2.74779900
H	-2.71273700	-2.23748100	-3.64810400
Si	4.36133300	-1.94901100	-1.35643900
Si	-3.95217100	0.28214600	-1.62590400
C	-5.66091600	-0.48225100	-1.41211200

H	-6.40717600	0.30050200	-1.19568900
H	-5.97037800	-1.00477200	-2.33258700
H	-5.66206900	-1.20238600	-0.58260600
C	-4.03602100	1.52529900	-3.04602700
H	-3.04301200	1.92468900	-3.29830400
H	-4.43796600	1.02684700	-3.94408000
H	-4.70261000	2.36825400	-2.80067200
C	4.29286900	-3.79687700	-1.72046600
H	4.49747000	-3.99498600	-2.78564200
H	5.04134400	-4.34228600	-1.12216300
H	3.29761000	-4.19990200	-1.47711200
C	6.05991400	-1.26110800	-1.79824500
H	6.84905500	-1.76804000	-1.21854600
H	6.27801800	-1.40797100	-2.86914600
H	6.10937400	-0.18290100	-1.58002500
C	-4.75552800	-1.84831300	2.52763200
C	-5.22520600	-2.71804200	1.53700800
C	-4.30963000	-3.45754000	0.77928800
C	-2.93798800	-3.33968800	1.01943500
C	-2.45770200	-2.48446800	2.02615900
C	-3.38344800	-1.73145100	2.76788000
H	-5.46045700	-1.25320500	3.11416200
H	-6.29786400	-2.81181000	1.35032400
H	-4.66458100	-4.12619500	-0.00928500
H	-2.22460200	-3.90627800	0.41947400
H	-3.01485200	-1.04485800	3.53114000
S	-0.71196700	-2.37537800	2.37801800

6. Reference

(1) Armarego, W. L. *Purification of laboratory chemicals*; Butterworth-Heinemann, 2017.

(2) Witten, M. R.; Jacobsen, E. N. A simple primary amine catalyst for enantioselective α-hydroxylations and α-fluorinations of branched aldehydes. *Org. Lett.* **2015**, *17*, 2772-2775.

(3) Greulich, T. W.; Yamaguchi, E.; Doerenkamp, C.; Lübbesmeyer, M.; Daniliuc, C. G.; Fukazawa, A.; Eckert, H.; Yamaguchi, S.; Studer, A. Synthesis and Physical Properties of Strained Doubly Phosphorus-Bridged Biaryls and Viologens. *Chem. - Eur. J.* **2017**, *23*, 6029-6033.

(4) Stoll, S.; Schweiger, A. EasySpin, a comprehensive software package for spectral simulation and analysis in EPR. *J. Magn. Reson.* **2006**, *178*, 42-55.

(5) Sheldrick, G. M. *SADABS–Bruker AXS area detector scaling and absorption*, Ver. 2008.

(6) Sheldrick, G. M. SHELXT–Integrated space-group and crystal-structure determination. *Found. Crystallogr.* **2015**, *71*, 3-8.

(7) Sheldrick, G. M. Crystal structure refinement with SHELXL. *Cryst. Struct. Commun.* **2015**, *71*, 3-8.

(8) Dolomanov, O. V.; Bourhis, L. J.; Gildea, R. J.; Howard, J. A.; Puschmann, H. OLEX2: a complete structure solution, refinement and analysis program. *Appl. Crystallogr.* **2009**, *42*, 339-341.

(9) Grimme, S.; Ehrlich, S.; Goerigk, L. Effect of the damping function in dispersion corrected density functional theory. *J. Comput. Chem.* **2011**, *32*, 1456-1465.

(10) Weigend, F.; Ahlrichs, R. Balanced basis sets of split valence, triple zeta valence and quadruple zeta valence quality for H to Rn: Design and assessment of accuracy. *Phys. Chem. Chem. Phys.* **2005**, *7*, 3297-3305.

(11) Zheng, J.; Xu, X.; Truhlar, D. G. Minimally augmented Karlsruhe basis sets. *Theor. Chem. Acc.* **2011**, *128*, 295-305.

(12) Miertuš̃, S.; Tomasi, J. Approximate evaluations of the electrostatic free energy and internal energy changes in solution processes. *Chem. Phys.* **1982**, *65*, 239-245.

(13) Miertuš, S.; Scrocco, E.; Tomasi, J. Electrostatic interaction of a solute with a continuum. A direct utilizaion of AB initio molecular potentials for the prevision of solvent effects. *Chem. Phys.* **1981**, *55*, 117-129.

(16) Frisch, M. J.; Trucks, G. W.; Schlegel, H. B.; Scuseria, G. E.; Robb, M. A.; Cheeseman, J. R.; Scalmani, G.; Barone, V.; Petersson, G. A.; Nakatsuji, H.; Li, X.; Caricato, M.; Marenich, A. V.; Bloino, J.; Janesko, B. G.; Gomperts, R.; Mennucci, B.; Hratchian, H. P.; Ortiz, J. V.; Izmaylov, A. F.; Sonnenberg, J. L.; Williams-Young, D.; Ding, F.; Lipparini, F.; Egidi, F.; Goings, J.; Peng, B.; Petrone, A.; Henderson, T.; Ranasinghe, D.; Zakrzewski, V. G.; Gao, J.; Rega, N.; Zheng, G.; Liang, W.; Hada, M.; Ehara, M.; Toyota, K.; Fukuda, R.; Hasegawa, J.; Ishida, M.; Nakajima, T.; Honda, Y.; Kitao, O.; Nakai, H.; Vreven, T.; Throssell, K.; Montgomery, J. A., Jr.; Peralta, J. E.; Ogliaro, F.; Bearpark, M. J.; Heyd, J. J.; Brothers, E. N.; Kudin, K. N.; Staroverov, V. N.; Keith, T. A.; Kobayashi, R.; Normand, J.; Raghavachari, K.; Rendell, A. P.; Burant, J. C.; Iyengar, S. S.; Tomasi, J.; Cossi, M.; Millam, J. M.; Klene, M.; Adamo, C.; Cammi, R.; Ochterski, J. W.; Martin, R. L.; Morokuma, K.; Farkas, O.; Foresman, J. B.; Fox, D. J. *Gaussian 16*, Revision C.01; Gaussian, Inc.: Wallingford CT, 2016.

(17) Glendening, E. D.; Badenhoop, J. K.; Reed, A. E.; Carpenter, J. E.; Bohmann, J. A.; Morales, C. M.; Karafiloglou, P.; Landis, C. R.; Weinhold, F. *NBO 7.0*; Theoretical Chemistry Institute, University of Wisconsin: Madison, 2018.
(18) Glendening, E. D.; Landis, C. R.; Weinhold, F. *NBO 7.0*: New vistas in localized and delocalized chemical bonding theory. *J. Comput. Chem.* **2019**, *40*, 2234-2241.
(19) Knizia G. Intrinsic atomic orbitals: An unbiased bridge between quantum theory and chemical concepts. *J. Chem. Theory Comput.* **2013**, *9*, 4834-4843.
(20) Knizia, G.; Klein, J. E. Electron flow in reaction mechanisms—revealed from first principles. *Angew. Chem., Int. Ed.* **2015**, *54*, 5518-5522.
(21) Lu, T. A comprehensive electron wavefunction analysis toolbox for chemists, Multiwfn. *J. Chem. Phys.* **2024**, *161*, 082503.
(22) Lu, T.; Chen, Q. Interaction region indicator: a simple real space function clearly revealing both chemical bonds and weak interactions. *Chem. Methods*, **2021**, *1*, 231-239.
(23) Humphrey, W.; Dalke, A.; Schulten, K. VMD: Visual Molecular Dynamics. *J. Mol. Graphics* **1996**, *14*, 33-38.
